# bootGSEA: a bootstrap and rank aggregation pipeline for multi-study and multi-omics enrichment analyses

**DOI:** 10.3389/fbinf.2024.1380928

**Published:** 2024-04-03

**Authors:** Shamini Hemandhar Kumar, Ines Tapken, Daniela Kuhn, Peter Claus, Klaus Jung

**Affiliations:** ^1^ Institute for Animal Genomics, University of Veterinary Medicine, Foundation, Hannover, Germany; ^2^ Center for Systems Neuroscience (ZSN), University of Veterinary Medicine, Foundation, Hannover, Germany; ^3^ SMATHERIA gGmbH—Non-Profit Biomedical Research Institute, Hannover, Germany; ^4^ Clinic for Conservative Dentistry, Periodontology and Preventive Dentistry, Hannover Medical School, Hannover, Germany

**Keywords:** bootstrap analysis, gene set enrichment analysis, multi-omics analysis, proteomics, rank aggregation, transcriptomics

## Abstract

**Introduction:** Gene set enrichment analysis (GSEA) subsequent to differential expression analysis is a standard step in transcriptomics and proteomics data analysis. Although many tools for this step are available, the results are often difficult to reproduce because set annotations can change in the databases, that is, new features can be added or existing features can be removed. Finally, such changes in set compositions can have an impact on biological interpretation.

**Methods:** We present bootGSEA, a novel computational pipeline, to study the robustness of GSEA. By repeating GSEA based on bootstrap samples, the variability and robustness of results can be studied. In our pipeline, not all genes or proteins are involved in the different bootstrap replicates of the analyses. Finally, we aggregate the ranks from the bootstrap replicates to obtain a score per gene set that shows whether it gains or loses evidence compared to the ranking of the standard GSEA. Rank aggregation is also used to combine GSEA results from different omics levels or from multiple independent studies at the same omics level.

**Results:** By applying our approach to six independent cancer transcriptomics datasets, we showed that bootstrap GSEA can aid in the selection of more robust enriched gene sets. Additionally, we applied our approach to paired transcriptomics and proteomics data obtained from a mouse model of spinal muscular atrophy (SMA), a neurodegenerative and neurodevelopmental disease associated with multi-system involvement. After obtaining a robust ranking at both omics levels, both ranking lists were combined to aggregate the findings from the transcriptomics and proteomics results. Furthermore, we constructed the new R-package “bootGSEA,” which implements the proposed methods and provides graphical views of the findings. Bootstrap-based GSEA was able in the example datasets to identify gene or protein sets that were less robust when the set composition changed during bootstrap analysis.

**Discussion:** The rank aggregation step was useful for combining bootstrap results and making them comparable to the original findings on the single-omics level or for combining findings from multiple different omics levels.

## 1 Introduction

Set-based enrichment methods are an integral part of the analysis of high-throughput expression data, such as those originating from transcriptomics or proteomics experiments. Enrichment methods allow the identification of molecular pathways, Gene Ontology (GO) terms, and other gene sets that might play a role in the disease of interest. Most enrichment methods are subsequently conducted for differential expression analysis; that is, they rely on the ranking of genes after comparing two groups of samples. Statistical tests are used to determine whether the genes of a particular set are disproportionately highly enriched among the differentially expressed genes (DEGs) (Beissbarth and Speed, 2004; Subramanian et al., 2005; Ackermann and Strimmer, 2009). This contrasts with self-contained gene set tests, which are based on subsets of expression data related to a particular gene set (Goeman et al., 2004; Hummel et al., 2008; Jung et al., 2011; Bayerlová et al., 2015).

Set information for enrichment analyses is usually obtained from public databases, for example, on molecular pathways or GO terms. The most commonly used databases are the “Reactome pathway knowledgebase” (Fabregat et al., 2016) (https://reactome.org/), “Kyoto Encyclopedia of Genes and Genomes” (Kanehisa and Goto, 2000) (KEGG; https://www.genome.jp/kegg/), “WikiPathways” database (Kelder et al., 2012) (https://www.wikipathways.org), “GO” knowledgebase (Consortium, 2004) (http://geneontology.org/), and the “Molecular Signatures Database” (Liberzon et al., 2011) (MSigDB; https://www.gsea-msigdb.org/gsea/msigdb). In the GO database, a particular GO term comprises a set of genes that can be assigned to a biological process (BP), molecular function (MF), or cellular component (CC).

The contents of the databases are curated either automatically by computer algorithms or manually by experts (Consortium, 2004). Specifically, WikiPathways provides community-based curation by registered contributors (Kelder et al., 2012). An example of a database where curation is done both ways, manually and computationally, is the MSigDB. Furthermore, pathway membership can be experimentally validated or predicted computationally. However, none of the modes of curation can prevent the uncertainties remaining regarding the membership of individual genes to a particular pathway (Gillis and Pavlidis, 2013). This is important because all enrichment analyses rely on the correctness of the database information, and the results of enrichment analyses would change if features of a set are removed or added. This can especially happen when the database information is retrieved at different times. For example, the GO database contained 42,442 terms classified as valid and 5,287 classified as obsolete in January 2024. Two months before, only 4,889 terms were classified as obsolete, meaning that nearly 400 terms would have to be reconsidered when a Gene-set enrichment analysis (GSEA) is performed after January 2024. In addition, the WikiPathways database reports roughly between 100 and 700 edits per month. Furthermore, in the KEGG database, complete pathways can be merged, leading to a large number of changes. For example, the KEGG pathway map00471 has been deleted and then added to the KEGG pathway map00470 (“D-amino acid metabolism”).

In this work, we present bootGSEA as a novel bootstrap approach to repeatedly sample subsets of pathways or other gene sets to study whether a result remains significant when the set composition is changed. The ranking lists of the gene sets obtained from each bootstrap replicate were aggregated using a score that can be used for a new ranking list. The analyst can then compare the original ranking with the bootstrap-based ranking list to study whether the association of a pathway or GO term with the disease gains or loses evidence. A similar approach was proposed by Schmid et al. (2016), who generated a robustness score for each gene set using random subsets of gene sets. In contrast to their approach, our method results in a new ranking of gene sets that can be helpful in aggregating findings from different independent studies or different omics levels. Thus, our approach for multi-omics follows the idea of aggregating the different omics levels after performing primary analysis on the individual levels first. This way of multi-omics analysis has also been implemented in other studies. For example, Wang et al. (2014) fused networks that were first derived on individual omics levels, Xiong et al. (2012) integrated genetic and transcriptomics results in a joint score, and Kang et al. (2022) fitted neural networks from individual omics levels and merged them into a joint model.

We also demonstrated that this method is useful for aggregating the results of enrichment analysis from different omics domains in the same experiment. We applied our new bootstrap pipeline to a single-omics scenario (transcriptomics only) comprising six independent renal cancer datasets. This example was used to show how our bootstrap pipeline can help study the robustness of GSEA when comparing results from multiple independent datasets from studies on a similar research question. In addition, we analyzed our multi-omics kidney data (transcriptomics and proteomics) from our research consortium on spinal muscular atrophy (SMA). The data were obtained from a SMA mouse model to demonstrate the usefulness of our approach when comparing GSEA results between different omics levels. SMA is a monogenic disease caused by the mutation or deletion of the survival motor neuron 1 (*SMN1*) gene (Lefebvre et al., 1995). The disease is characterized by the degeneration of motoneurons, with the subsequent atrophy of skeletal muscles to muscular atrophy since the SMN affects all tissues, which also include non-skeletal muscles. Moreover, SMA is a multi-system disorder that also affects peripheral organs, such as the kidney (Allardyce et al., 2020). Three treatment methods are available, all increasing SMN expression. The SMN is expressed ubiquitously and has several important cellular functions, including snRNP assembly, R-loop resolution, and regulation of the actin cytoskeleton and translation (Hensel et al., 2020). Therefore, SMA is a highly complex disease with expected dysregulations in pathways in several cell types and on several molecular levels.

## 2 Methods

**FIGURE 1:**
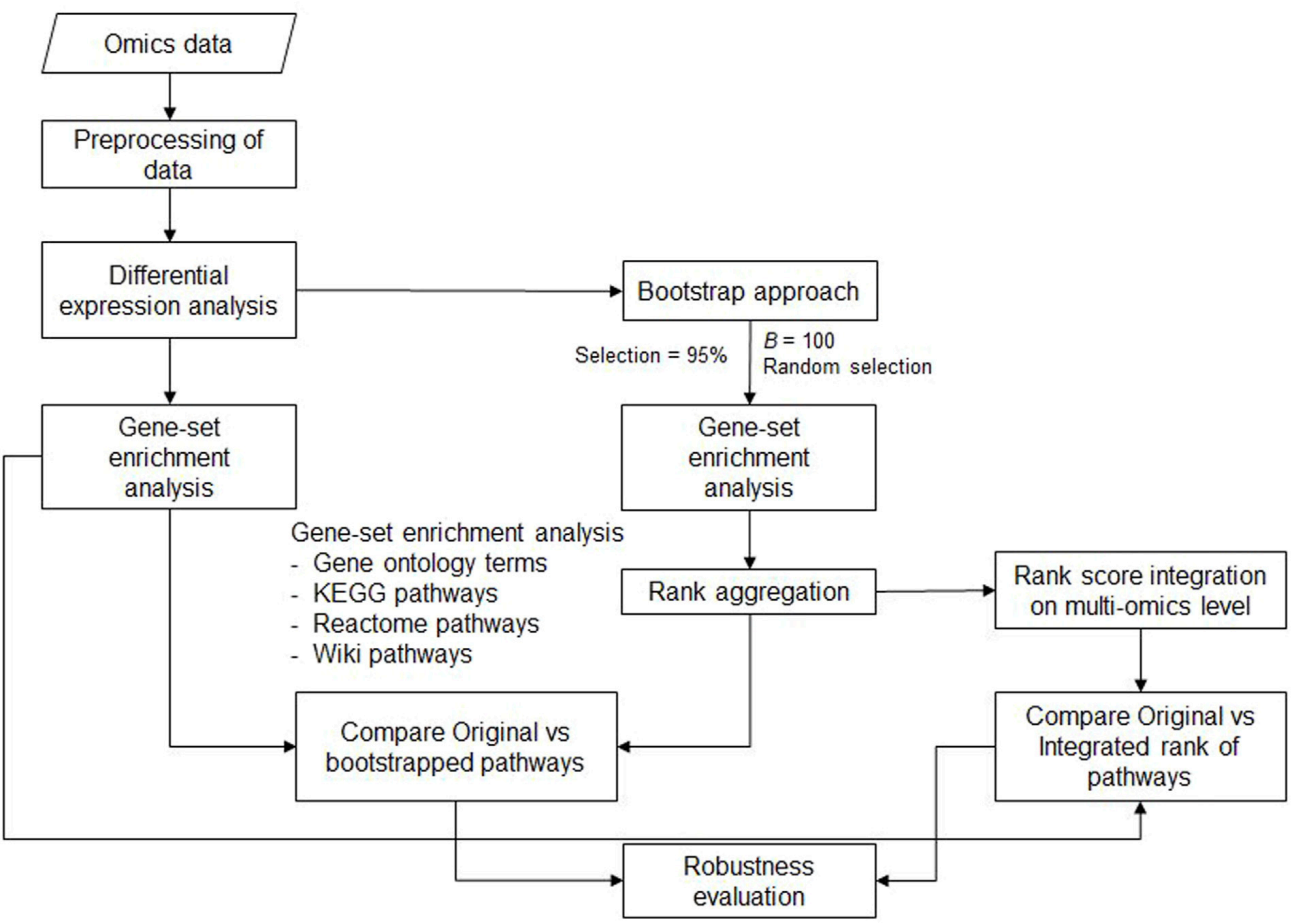
Workflow of the analysis pipeline for bootstrap enrichment analysis at the single-omics level and the steps for single- and multi-omics rank aggregation. For the data obtained from each omics domain (transcriptomics and proteomics data), Gene-set enrichment analysis (GSEA) is performed after differential expression analysis using all data (original standard analysis). Next, gene set enrichment analysis is performed on subsets of data based on bootstrap samples. Finally, the different rankings of the GO terms or pathways can be integrated not only within each omics level but also across all omics levels. The robustness of gene set enrichment analysis can be studied by comparing the original results with either single- or multi-omics results.

In this section, we describe the analysis pipeline, including the approach for the bootstrap step used to repeatedly analyze different random subsets of the data. Furthermore, the rank aggregation step and examples of transcriptomics and proteomics data are presented. The complete workflow is shown in Figure 1. All the analyses were implemented in the R programming environment [www.r-project.org, version 4.2 (R Core Team, 2022)].

### 2.1 Differential expression analysis and bootstrap method for Gene-set enrichment analysis

Prior to enrichment analysis, differential expression analysis was performed on normalized expression data from different groups of interest (for example, disease vs. control) to obtain the ranks of genes and proteins. For the microarray and proteomics data, normalization was performed using the quantile method (Bolstad et al., 2003; Välikangas et al., 2018; Zhao et al., 2020), and for the RNA-seq data, the internal normalization of the DESeq method of the R package “DESeq2” was used (Love et al., 2014). Differential analysis of the proteomics data was performed using the functionality of the R package “limma” (Ritchie et al., 2015), and differential analysis of the RNA-seq data was done using the R package “DESeq2” (spinal muscular atrophy data) and microarray data using “limma” (Ritchie et al., 2015) (renal cell carcinoma datasets).Next, enrichment analysis based on the results of the differential expression analysis was initially performed using the complete dataset, that is, using all genes or proteins that were assigned to a particular gene set according to the database information. We denote this as the original analysis. Gene sets were defined based on pathway data from the KEGG, Reactome, and WikiPathways databases, as well as GO terms. KEGG, Reactome, and WikiPathways enrichment analyses were performed using the R package “clusterProfiler” (Wu et al., 2021), and GO term enrichment analyses were performed using the R package “topGO” (Alexa and Rahnenführer, 2009). The enrichment analyses in “clusterProfiler” implement the methods described by Subramanian et al. (2005), which are independent of thresholds for differentially expressed features. In contrast, the “topGO” method uses a threshold but has the advantage of incorporating information about the hierarchy of GO terms.

To study the variability and robustness of the outcome of the enrichment analysis, *B* bootstrap samples were drawn using only 95% of all the genes in each run. The genes were randomly drawn without replacement. GSEA was repeated for these randomly selected subsets of genes *B* times, where *B* is the number of times the whole set was resampled. The composition of the defined gene sets changed when bootstrapping from the gene sets was performed. Consequently, the composition of the gene sets was different for each bootstrap run. Thus, the effect of individual genes was also reflected in this approach. From this bootstrap procedure, *B* ranking lists of the gene sets were obtained.

### 2.2 Rank aggregation for single- and multi-omics analyses

The resulting enrichment analysis from the *B* bootstrap runs with *B* lists of GO terms was aggregated using the R package “RobustRankAggreg” (Kolde et al., 2012). The aggregation score for each pathway was obtained based on the number of occurrences and the ranks from each bootstrap run. The aggregation score was further transformed into a rank for each pathway. To study the robustness of the original findings, the rank obtained from the aggregated score can be compared with the actual analysis, that is, the analysis without a bootstrap step.

For multi-omics data, original and bootstrap enrichment analyses were first performed for each omics domain, resulting in one list of aggregated ranks per domain. The aggregated scores from each omics domain were further aggregated. In one of our data examples, enrichment analysis was performed separately for the transcriptomics and proteomics data, and both ranking lists were aggregated into one final ranking list. Thus, the final multi-omics score for each pathway or GO term was obtained.

### 2.3 R package: bootGSEA

The workflow shown in Figure 1 has been compiled and implemented in the new R package “bootGSEA” available at the GitHub repository (https://github.com/klausjung-hannover/bootGSEA). The input requires the results of differential expression analysis. The package currently has eight functions. The functions boot.GO and boot.pathway are used for GO and pathway enrichment analyses, respectively, of the complete data (original analysis) and of bootstrapped data samples, and aggr.boot.GO and aggr.boot.pathway are used for the rank aggregation of pathways obtained from the former functions. In the functions boot.GO and boot.pathway, the user can specify which percentage of features should be drawn during the bootstrap runs. Furthermore, to understand the robustness of pathways at a broader level, we used a multi-omics approach by aggregating ranks from individual omics levels using the aggr.multiomics function. In addition, three functions are provided to visualize these results and study the robustness of the findings. Examples of these visualizations are presented in Section 3. The function compareRank was implemented to compare the original and bootstrapped results at a single-omics level, the function plotRank, for both single- and multi-omics levels, and the function histDiff to understand the rank difference between original and bootstrap analyses.

### 2.4 Example data 1: transcriptomics data from a renal cancer study

The gene expression profiles of renal cell carcinoma (RCC) datasets (GSE6344 (Copland, 2008), GSE14762 (Dykema and Furge, 2009), GSE11024 (Kort, 2008), GSE14994 (Signoretti and Beroukhim, 2010), GSE53757 (John Copland et al., 2014), and GSE15641 (Jones et al., 2005)) were downloaded from the Gene Expression Omnibus (GEO) database using the GEOquery (Davis and Meltzer, 2007) package in the R platform. Detailed information about the datasets, including platform and sample size, is given in Table 1. Differential expression analysis was performed using the limma (Ritchie et al., 2015) package in R. DEGs were screened based on FDR-adjusted *p*-values < 0.05 as the cut-off value for all datasets. The results from the differential analysis were further analyzed following the pipeline shown in Figure 1 at the single-omics level.

**TABLE 1:** Renal cell carcinoma (RCC) datasets with accession numbers from the GEO database, sample sizes in the normal and tumor groups, and references to the publication of the original analysis.

Accession no.	Platform	n Normal	n Tumor	Reference
GSE6344	GPL96	10	10	Copland (2008)
GSE14762	GPL4866	12	10	Dykema and Furge (2009)
GSE11024	GPL6671	12	67	Kort (2008)
GSE14994	GPL3921	11	59	Signoretti and Beroukhim (2010)
GSE53757	GPL570	72	72	John Copland et al. (2014)
GSE15641	GPL96	23	69	Jones et al. (2005)

### 2.5 Example data 2: transcriptomics and proteomics data from a study on SMA

Severe (“Taiwanese”) SMA mice [(FVB.Cg-Tg (SMN2)2Hung Smn1tm1Hung/J)] (Hsieh-Li et al., 2000) were bred by an established breeding scheme (Riessland et al., 2010), resulting in a litter of half SMA mice (tgSMN2tg/0, mSmn1−/−) and half control mice (tgSMN2tg/0, mSmn1+/−). For analysis, the animals were euthanized by decapitation on pre-symptomatic post-natal day 3 (P3), and a tail tip biopsy was taken for genotyping, as described previously (Hensel et al., 2012). The kidneys were dissected, snap-frozen in liquid nitrogen, and stored at −80°C until analysis. Tissue was lyzed either for RNA-seq or for proteomics analyses, respectively, as described previously (Allardyce et al., 2020; Hensel et al., 2021), using total organ and total RNA. All animal experiments were conducted in accordance with the German Animal Welfare law and approved by the Ministry of Food, Agriculture, and Consumer Protection of Lower Saxony (LAVES file no. 19/3309).

The datasets including 54,146 mRNA transcripts and 7,959 proteins from the kidney samples of severe SMA and heterozygous control littermates were used for analysis. These data were used to evaluate our new bootstrap and rank aggregation approach in view of multi-omics data. Transcriptomic data included two control and two SMA-pooled samples, and proteomics data included four control and four SMA samples. Differential expression analysis was performed for the transcriptomic and proteomic data based on the control and SMA groups. These differentially expressed genes and proteins were further analyzed to determine the enriched pathways and GO terms using the pipeline (Figure 1).

## 3 Results

The bootGSEA pipeline used the results from the differential expression analysis as input, and GSEA was performed separately for the following types of gene sets: GO terms (BP, MF, and CC), KEGG, Reactome, and WikiPathways databases. The enrichment analysis performed in this pipeline provided two ranking lists of gene sets as outputs: one list from the original analysis of the complete data and one aggregated list from the analysis of the bootstrap samples. The original analysis included the entire list of genes or proteins from differential expression analysis. The bootstrap analysis involved taking the subsampled lists of genes or proteins for enrichment analysis, providing *B* additional ranking lists of results for enrichment analysis. To further determine the robustness of the bootstrap analysis, we aggregated the *B* lists to make them comparable to the ranking of the original analysis. All analyses were initially performed at a single-omics level.

For multi-omics analysis, rank scores from single-omics levels (transcriptomics and proteomics) were further integrated by rank aggregation to obtain an integrated score for the pathways or GO terms retrieved.In the following sections, we describe the results of the differential expression analysis, original findings of the GSEA, bootstrap enrichment analysis, and aggregated results from the six cancer datasets and the two omics levels from the SMA data. First, the results for the renal cancer data are shown, followed by the results for the multi-omics data from SMA mouse kidney samples.To compare the ranking obtained from the original and bootstrap GSEA, we mainly described rank gains and losses of individual GO terms or pathways. We avoided using the correlation coefficient since a correlation of, for example, 0.90 sounds high but can still include large rank differences. Only for the comparison between transcriptomics and proteomics in the SMA example did we use Kendall’s *τ* to describe the advantage of the bootstrap approach.

### 3.1 Example 1: analysis of renal cancer data

The six microarray datasets were downloaded from the GEO database using the GEOquery package in R. The total number of mRNA transcripts in each dataset used for differential expression analysis was as follows: GSE6344 with 21,225 transcripts, GSE11024 with 17,637 transcripts, GSE14762 with 17,232 transcripts, GSE15641 with 21,225 transcripts, GSE14994 with 21,238 transcripts, and GSE53757 with 44,134 transcripts. We used these data as examples to demonstrate how bootstrap GSEA can help study the robustness of the results across the six datasets.

#### 3.1.1 Differential expression analysis

Differential expression analysis of mRNA transcripts was performed using the limma package in R for all datasets, and DEGs were filtered based on an adjusted *p*-value < 0.05. The number of DEGs retrieved was as follows: 6,947 for GSE6344; 4,199 for GSE11024; 7,256 for GSE14762; 9,665 for GSE15641; 10,465 for GSE14994; and 27,968 for GSE53757.

#### 3.1.2 Gene set enrichment analysis

GSEA for each of the six datasets was performed individually, with the total number of transcripts from the differential expression analysis as input. GSEA was performed using the function boot.GO for GO enrichment analysis and boot.pathway for pathway enrichment analysis, obtained from our new package bootGSEA. The analyses were performed separately for each dataset. For example, for the GSE6344 dataset, BP-GO analysis was performed using the boot.GO function, which provides two lists of results: the original analysis and bootstrap analysis. With *B* = 100, 100 ranking lists were obtained for the bootstrap analysis. Following this analysis, the rank aggregation approach using aggr.boot.GO function was used to build a score representing the bootstrap analysis. This analysis resulted in a table with GO terms, ranks from each bootstrap run, and the aggregated rank score for each GO term. Similarly, analysis was performed for MF, CC, and pathway analyses (KEGG, Reactome, and WikiPathways) using the boot.pathway function. The resulting table consists of GO terms or pathways, aggregated scores, individual ranks of GO terms or pathways from bootstrap runs, original ranks, Fisher’s *p*-value from the original analysis, and bootstrap ranks based on the aggregated score. A summary of the number of annotated GO terms and pathways is given in Table 2.

**TABLE 2:** Summary information about the gene set enrichment analysis (GSEA) on the six renal cancer datasets. Displayed numbers per dataset are the total number of annotated GO terms or pathways and the number of enriched GO terms or pathways based on Fisher’s *p*-value < 0.05 for the original analysis.

GSE ID	GSEA	No. of GO terms or pathways	
		Annotated	Significant in the original analysis
GSE11024	GO:BP	15,444	1,643
	GO:MF	4,684	525
	GO:CC	1,939	329
	KEGG	343	204
	Reactome	1,489	410
	WikiPathways	600	191
GSE11024	GO:BP	15,847	1,465
	GO:MF	4,900	424
	GO:CC	1,977	295
	KEGG	344	55
	Reactome	1,527	93
	WikiPathways	605	57
GSE14762	GO:BP	15,799	5,029
	GO:MF	4,889	831
	GO:CC	1,978	521
	KEGG	344	64
	Reactome	1,515	69
	WikiPathways	603	41
GSE15641	GO:BP	15,445	1,876
	GO:MF	4,659	537
	GO:CC	1,976	336
	KEGG	343	294
	Reactome	1,489	340
	WikiPathways	600	179
GSE14994	GO:BP	15,417	1,796
	GO:MF	4,672	204
	GO:CC	1,942	180
	KEGG	342	171
	Reactome	1,490	594
	WikiPathways	610	202
GSE53737	GO:BP	15,927	1,184
	GO:MF	4,944	157
	GO:CC	1,986	108
	KEGG	344	255
	Reactome	1,543	872
	WikiPathways	625	348

#### 3.1.3 Robustness analysis of pathways and GO terms

**FIGURE 2:**
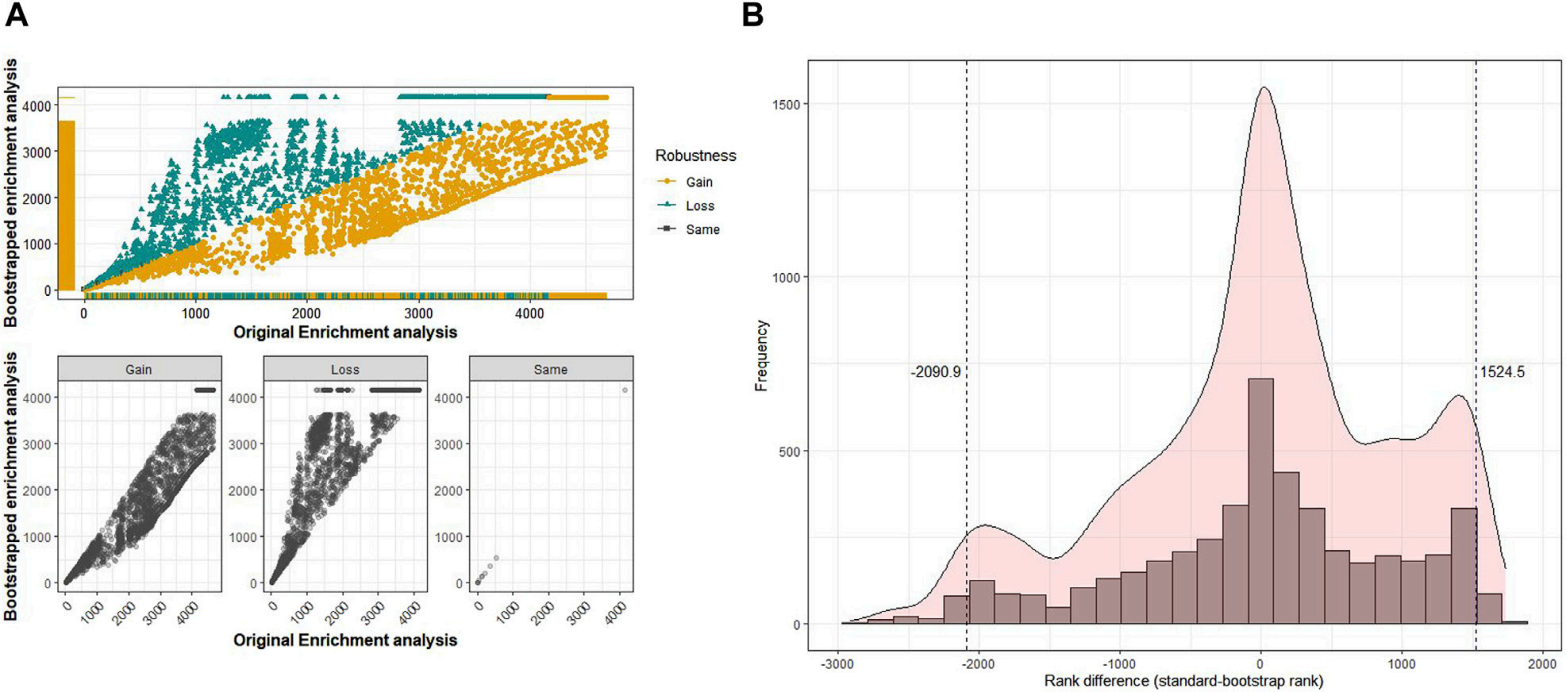
Comparison of the original analysis and bootstrap analysis for Gene Ontology (GO) terms of molecular functions in the renal cancer dataset GSE6344. **(A)** Top: along the *x*-axis are the ranks from the original GSEA, and the *y*-axis corresponds to the ranks from the bootstrap GSEA. The GO terms that have gained, lost, or retained the same rank after 100 bootstrap runs are shown separately in the plots at the bottom. **(B)** Histogram of differences in ranks between the original rank and aggregated rank from the bootstrap GSEA.

Robustness analysis of the pathways and GO terms to evaluate the stability of the identified pathways and GO terms across 100 iterations versus the original analysis was performed as follows. First, a scatter plot was used to identify pathways or GO terms with a high degree of variability in ranks between the original and bootstrapped analyses (ranks based on scores from rank aggregation). Scatter plots for each pathway analysis (KEGG, Reactome, and WikiPathways) and GO terms (BP, MF, and CC) were analyzed for the six datasets. In the following paragraphs, we describe the results exemplarily for the MF-GO enrichment analysis in dataset GSE6344. For the remaining results of this and the other cancer datasets, we refer to Supplementary Figures S1–S35. The distribution of the GO-MF terms for dataset GSE6344 is shown in Figure 2A. The *x*-axis displays the ranks from the original analysis, while the *y*-axis shows the aggregated ranks from the bootstrap analysis. The top scatter plot shows all pathways with gain, loss, or retained ranks. The bottom scatter plots show the individual distribution scales of the gain, loss, or retained ranks. Only very few gene sets had the same rank in the original and bootstrap analyses. Therefore, the gain or loss of ranks indicates a certain level of variability in comparison. The higher the variability, the less robust the findings of the original analysis. To further understand this variability of ranks in GO terms, the distribution of rank difference between the original and bootstrapped analyses was analyzed (Figure 2B). We determined the quantiles of this distribution to identify terms with extreme gains or losses. Specifically, GO terms that fall below the 2.5% or exceed the 97.5% quantile have a larger rank difference and are, therefore, less reliable and more susceptible to variation in the data. In this example, the minimum size for rank losses below the 2.5% quantile is 2,091; that is, gene sets that fall below this quantile have a difference in rank of at least 2,091. The minimum rank gain at the 97.5% quantile was 1,525. These gene sets, with such differences in rank, are considered not robust and, therefore, appear rather unsuitable for biological interpretation.

**FIGURE 3:**
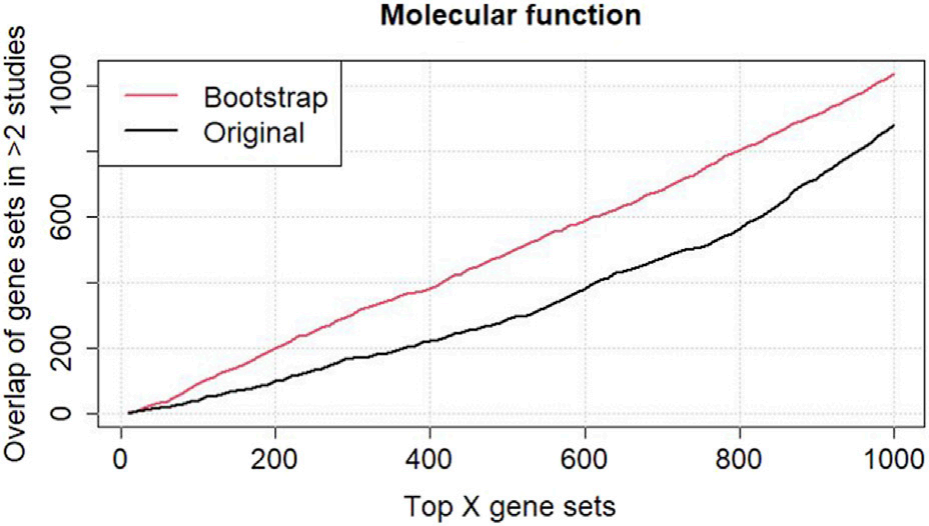
Comparison analysis between the original and bootstrapped analyses of common GO terms in molecular function among the six renal cell carcinoma (RCC) datasets. A higher agreement between the six datasets is observed when using the ranking of GO terms according to the bootstrap analysis.

To better understand and assess the robustness of the gene sets identified in the six independent datasets, the original and bootstrap analyses of the common terms/pathways in at least two datasets among the top 1,000 gene sets ordered based on their Fisher’s values and aggregated scores from the bootstrap analysis have been performed (Figure 3 Supplementary Figure S36). The plot of GO-MF terms shows a clear difference between the original and bootstrap analyses, indicating that the gene sets found by the original standard analysis might not be robust and require further investigation. In contrast, the gene sets were consistent across the original and bootstrap analyses (Supplementary Figure S37, line plots of KEGG, Reactome, and WikiPathways), indicating that the gene sets were robust and reliable when comparing the results from the six independent datasets.

**FIGURE 4:**
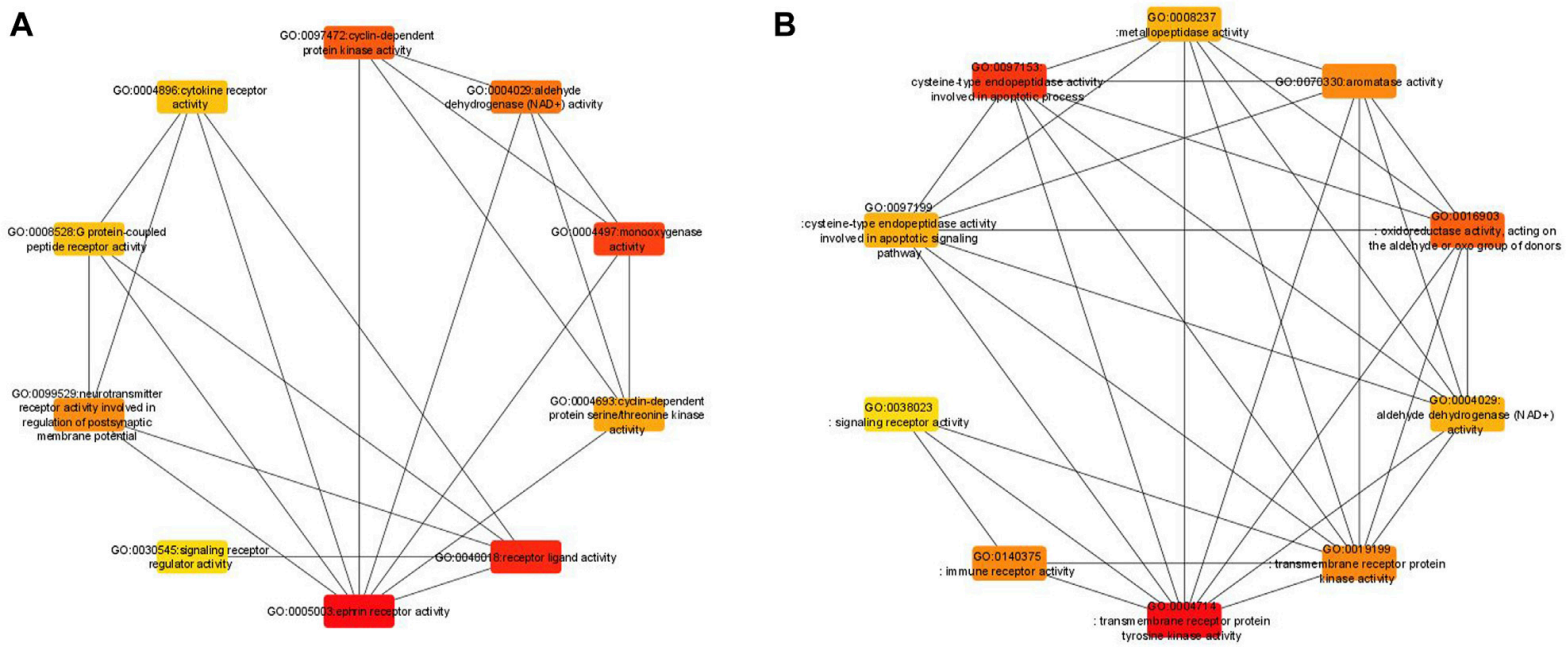
Comparative network analysis between the original (left) and bootstrapped (right) analyses, including the top 10 where each node represents the GO term among the six RCC datasets.

To further evaluate the robustness of our analysis, a network-based approach was performed for the 58 GO-MF terms obtained from the enrichment analysis of all 6 datasets from the bootstrap and original analyses. The 58 GO-MF terms (Supplementary Table S1) were selected by combining the bootstrap aggregated results of all 6 datasets. The same procedure was performed for the original enrichment analysis. A network was then constructed for these 58 GO terms in REVIGO (Supek et al., 2011), with each GO term as a node and edges between the nodes if there is a significant correlation between the corresponding gene sets. The constructed network was visualized in Cytoscape (Shannon et al., 2003) to evaluate the robustness of the network with one of the network metrics in cytoHubba available in Cytoscape. We used the radiality metric to evaluate the robustness. Bootstrap analyses have higher well-connected GO terms in the network when the top 10 GO terms of the original and bootstrap analyses are ranked based on their radiality metric (Figure 4). Comparatively, the original analysis has fewer connections, indicating that the bootstrap analyses provide more robust results.

### 3.2 Example 2: multi-omics of the kidney in a SMA mouse model

#### 3.2.1 Differential expression analysis

Transcriptomic and proteomic data were pre-processed for differential expression analysis. This processing included the imputation of missing values in the proteomics data, for which the KNN method implemented in the R package “impute” was used. Differential expression analysis using “DESeq2” and the “limma” package in R was performed for transcriptomic and proteomic data, respectively. The analysis retrieved 29,596 transcripts and 7,959 proteins. A total of 81 DEGs and 148 differentially expressed proteins (DEPs) were selected based on the criteria of *p*-value < 0.05 and |*logFC*| > 1.

#### 3.2.2 Gene set enrichment analysis

GSEA for the original and bootstrap analyses was performed separately at the transcriptomic and proteomic levels using our new R package bootGSEA with the functions boot.GO (for GO analysis) and boot.pathway (for KEGG, Reactome, and WikiPathways). The input data were the results obtained from the differential expression analyses. The analyses resulted in two outputs: original analysis and bootstrapped analysis. The bootstrapped analysis included *B* = 100 lists of enrichment analyses based on random subsets of genes and proteins. These 100 lists of enrichment results were aggregated by rank aggregation using the functions aggr.boot.GO (for GO analysis) and aggr.boot.pathway (for KEGG, Reactome, and WikiPathways analyses), where the input is the output from the functions boot.GO and boot.pathway.This analysis was performed for all GO terms (BP, MF, and CC), KEGG, Reactome, and WikiPathways at a single-omics level (transcriptomics and proteomics data individually) of the SMA kidney data (Table 3).

**TABLE 3:** Summary of the gene set enrichment analysis (GSEA) of the spinal muscular atrophy (SMA) mouse data. The total number of annotated GO terms and pathways and the number of enriched GO terms and pathways based on Fisher’s value < 0.05 for the original analysis are given.

GSEA	No. of GO terms or pathways retrieved			
	Transcriptomics		Proteomics	
	Annotated	Significant in the original analysis	Annotated	Significant in the original analysis
GO:BP	15,995	228	13,670	130
GO:MF	4,837	99	3,893	58
GO:CC	2,020	27	1,829	18
KEGG	337	5	305	22
Reactome	1,093	38	617	30
WikiPathways	150	7	89	6

#### 3.2.3 Robustness analysis of pathways and GO terms at the single-omics level

**FIGURE 5:**
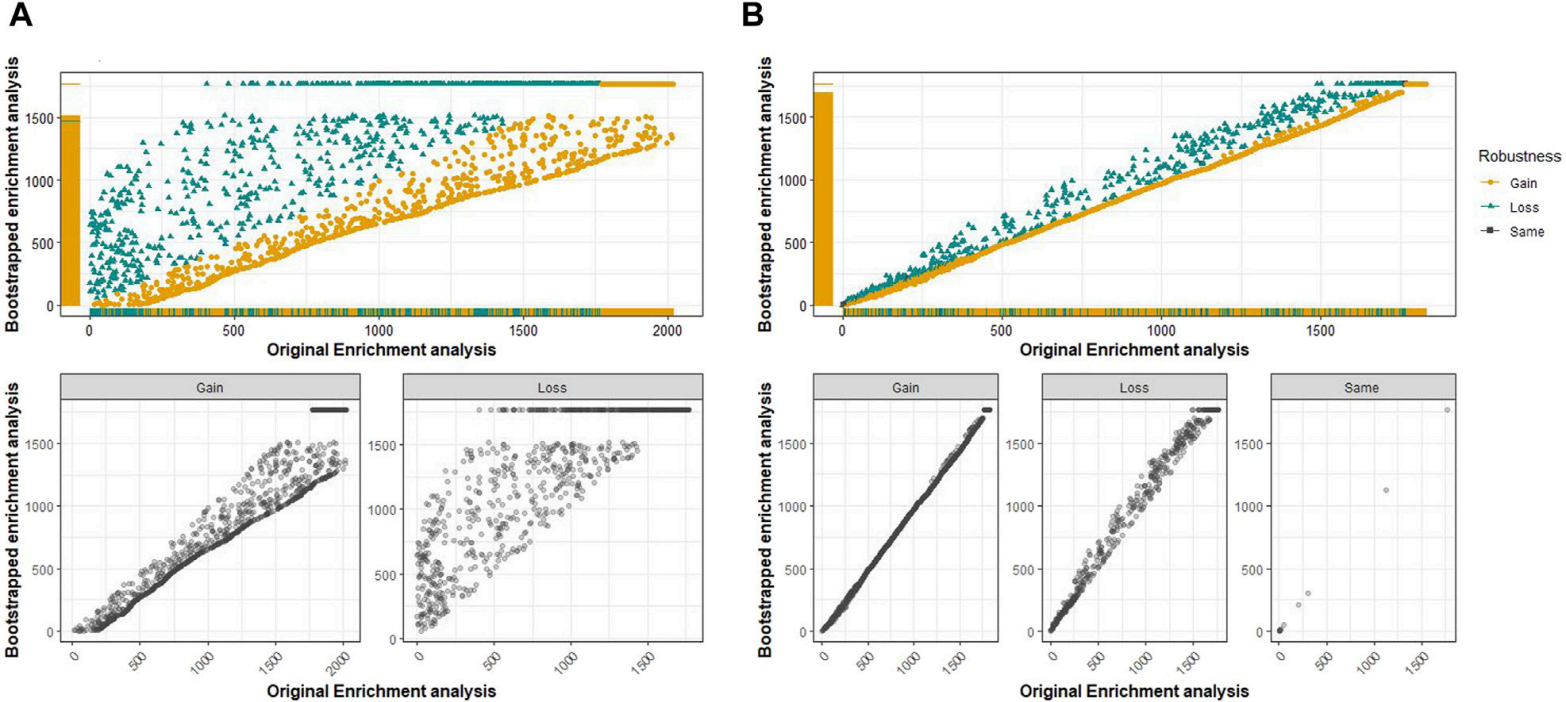
Comparison of the original and bootstrapped analyses for the GO term of cellular components in the transcriptomics **(A)** and proteomics **(B)** spinal muscular atrophy (SMA) kidney data. Top plots: along the *x*-axis is the original enrichment analysis, and the *y*-axis corresponds to the bootstrapped enrichment analysis. The GO terms that have gained, lost, or retained the same rank after *B* = 100 bootstrap runs are shown separately in the plots at the bottom.

The original and bootstrapped analyses for the GO terms and pathways of transcriptomics and proteomics data were compared using a scatter plot to analyze the distribution of ranks for their robustness (Figure 5). The GO-CC terms that show an increase in robustness from our pipeline are shown as gain of rank, the terms that lost their rank after *B* bootstrap runs show lesser robustness, and those terms that retained their ranks even after 100 runs retained their ranks. However, the comparison of transcriptomic analyses did not result in any retained ranks (Figure 5A). Therefore, all terms either gained or lost their ranks in this analysis. Proteomics analysis (Figure 5B), on the other hand, had very few retained ranks. The same comparison analysis was performed for the other GO terms and pathways (Supplementary Figures S38–S42). It should be noted that transcriptomic analysis for both BP and MF had only one term with a retained rank of 11,489 and 1,249, respectively. However, both terms (GO:0072429 and GO:0004985) were ranked very low, indicating that they were not significantly enriched.

**FIGURE 6:**
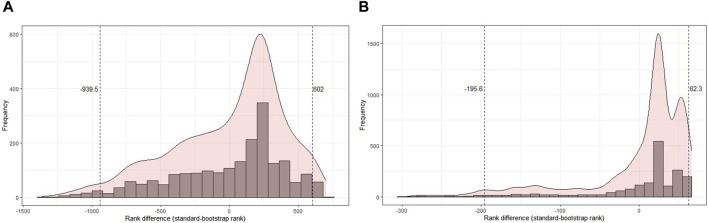
Histogram of the difference in rank of the original rank and the aggregated rank of the bootstrapped analysis in cellular components for transcriptomics and proteomics SMA kidney data. **(A)** Distribution of rank difference in the transcriptomics data of 2,020 terms, where cellular component terms beyond the 2.5% and 97.5% quantiles have a difference of 939.5 and 602, respectively. **(B)** Distribution of rank difference in the proteomics data of 1,829 terms, where cellular component terms beyond the 2.5% and 97.5% quantiles have a difference of 195.6 and 62.3, respectively.

Next, the distribution of rank differences between the original and bootstrapped analyses of the GO-CC terms and pathways was analyzed (Figure 6). The terms and pathways that fall below the 2.5% or exceed the 97.5% quantiles have a very large difference between the original and bootstrapped analyses and should not be considered for biological interpretation. Quantiles to specify terms with extreme changes between the original and bootstrap analyses were determined (Figure 6). For transcriptomics data (Figure 6A), the minimum rank difference for the lost rank below the 2.5% quantile is −939.5, that is, GO terms below this quantile have a difference of equal to or more than 939.5 between the original analysis and bootstrap analysis. The minimum rank gain at the 97.5% quantile was 602. Such huge differences in ranks mean that the terms below or above these quantiles should rather not be considered for biological interpretation. For proteomics data (Figure 6B), there was a rank difference of 195.6 and 62.3 for lost and gained ranks, respectively. Histograms for the difference between original and bootstrap rankings related to other GO terms and pathways are given in Supplementary Figures S43–S47.

#### 3.2.4 Multi-omics analysis

Transcriptomics and proteomics data analyzed individually using the proposed pipeline yielded several GO terms and pathways with increased or decreased robustness, while only few GO terms and pathways retained the same rank after *B* bootstrap runs at the single-omics level. These terms or pathways were further analyzed for common terms using a Venn diagram, where an integrated score for these common terms was obtained by aggregating the ranks between omics levels.

**FIGURE 7:**
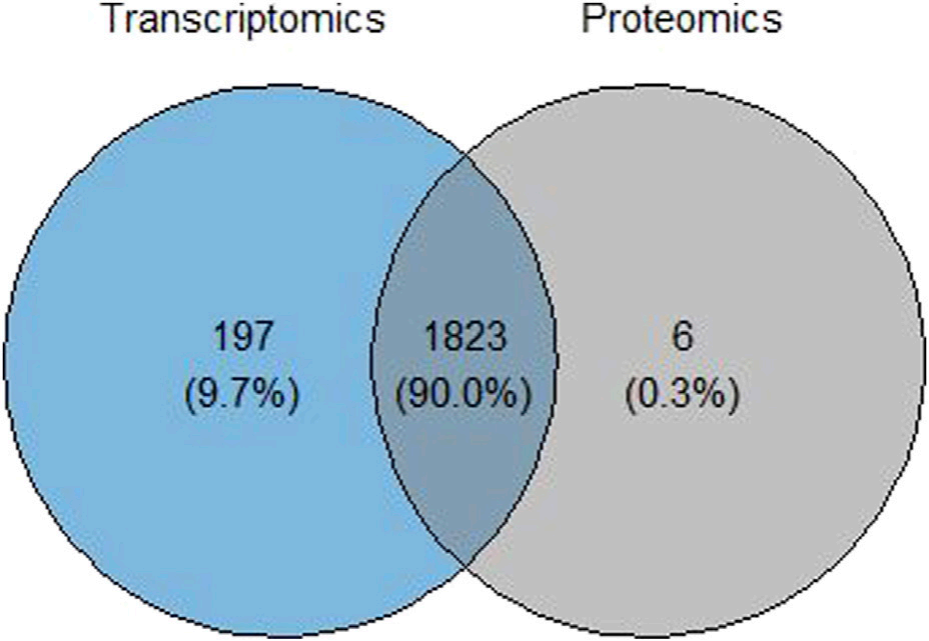
Venn diagram showing the overlap of significant GO terms for cellular components in the transcriptomics and proteomics data of the SMA study.

**FIGURE 8:**
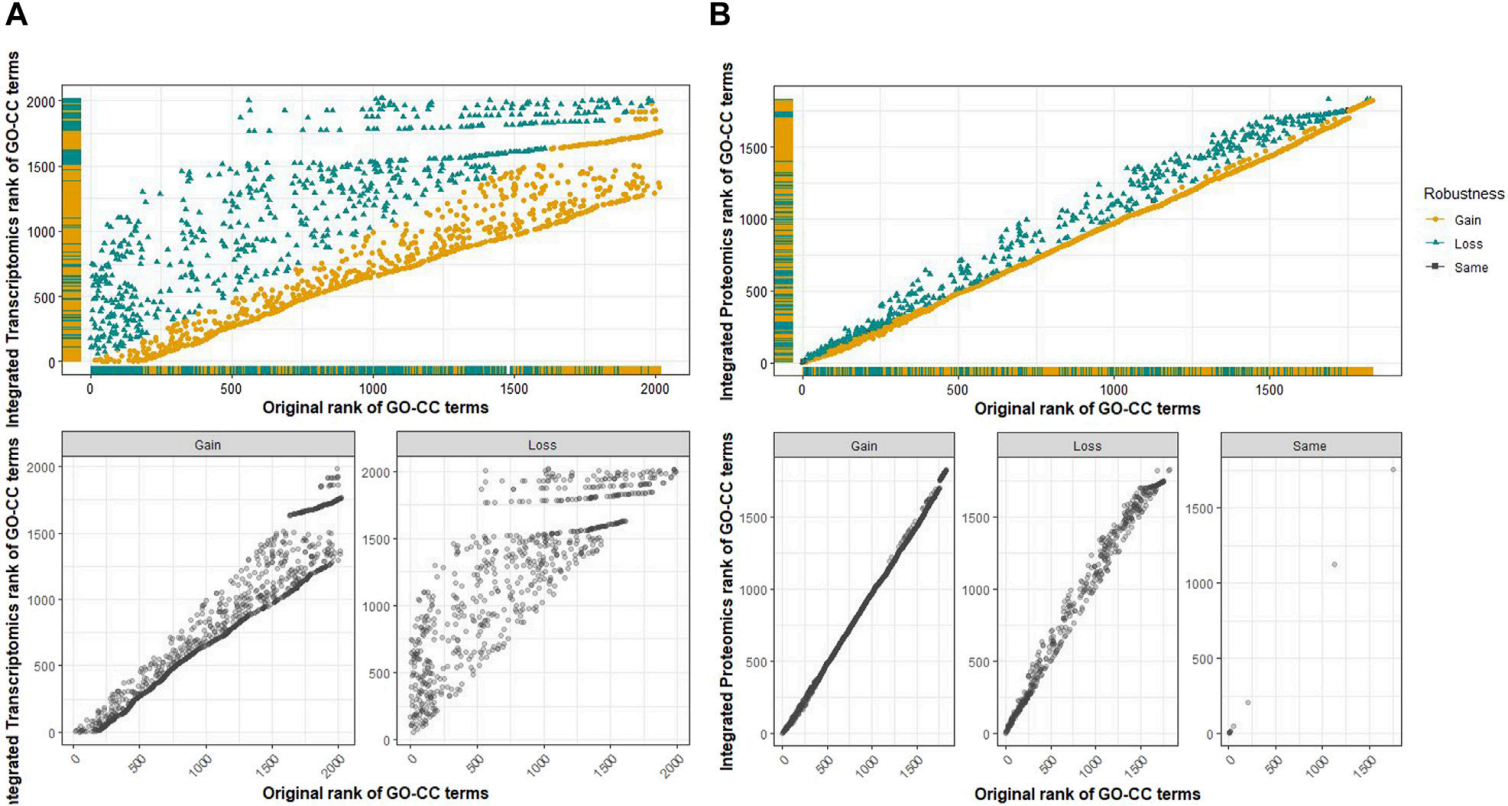
Scatter plot for the evaluation of the robustness of GO-CC terms comparing the rank of the original analysis and the rank obtained from the integrated score of common terms at the multi-omics level. **(A)** Top: along the *x*-axis is the original rank of transcriptomic analysis, and the *y*-axis corresponds to the rank of common GO-CC terms based on the integrated score. The gained and lost ranks of the GO-CC terms are shown separately in the bottom plots. No ranks were retained in this comparison. **(B)** Top: along the *x*-axis is the original rank of the proteomics analysis, and the *y*-axis corresponds to the rank of common GO-CC terms based on the integrated score. The gained, lost, and retained ranks of the GO-CC terms are presented separately in the bottom plots. Very few terms have retained ranks, meaning that most terms have either gained or lost their robustness after 100 bootstrap runs.

Common cellular component terms among the transcriptomic and proteomic omics levels were retrieved using a Venn diagram (Figure 7). Of the 2,020 terms from the transcriptomics analysis and 1,829 terms from the proteomics analysis, there were 1,823 common GO-CC terms from the analysis, for which an integrated score was obtained for these common terms. A plot comparing the integrated rank score and the original analysis was constructed at each omics level to analyze and evaluate the robustness of the common cellular component terms (Figure 8). The GO-CC terms that showed an increase in robustness from our pipeline were shown as a gain of rank, whereas the terms that lost their rank after 100 bootstrap runs were indicated as a decrease in robustness. The terms that retained their ranks even after 100 runs were deemed robust. However, the comparison of transcriptomics analyses did not result in any retained ranks (Figure 8A), indicating that all terms either gained or lost their rank in this analysis. In contrast, the proteomics analysis (Figure 8B) had very few retained ranks, suggesting that the terms with retained ranks are robust and other terms that have either gained or lost rank ranks are subject to uncertainty.

**FIGURE 9:**
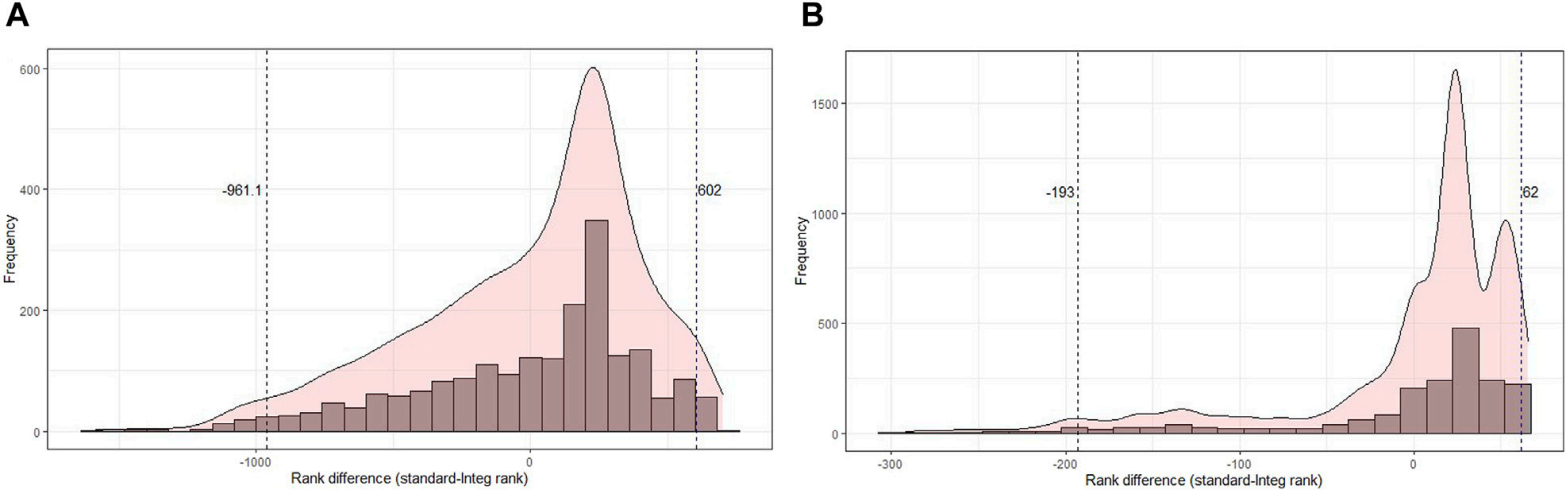
Histogram of the difference in rank of the original rank and the rank obtained from the integrated score of common terms at the multi-omics level. **(A)** Distribution of rank difference in transcriptomics data, where GO-CC terms beyond the 2.5% and 97.5% quantiles have a difference of 961 and 602, respectively. **(B)** Distribution of rank difference in proteomics data, where GO terms beyond the 2.5% and 97.5% quantiles have a difference of 193 and 62, respectively.

To further understand this variability of ranks (gains and losses) in GO terms, we analyzed the distribution of rank differences between the original and integrated analyses (Figure 9). We determined the quantiles of this distribution to identify terms with extreme gains or losses. Specifically, GO terms that fall below the 2.5% or exceed the 97.5% quantile have a larger rank difference and are, therefore, less reliable and more susceptible to variation in data. These gene sets with such large differences in rank are considered less robust and rather not suitable for biological interpretation.

The same procedure was performed for the other GO terms and pathways, where the Venn diagrams of the common terms are given in Supplementary Figure S48. Next, a plot to compare the distribution of ranks between the original analysis and the integrated rank score for these common terms and pathways are given in Supplementary Figures S49–S53. Histograms showing the difference in rank based on the integrated score and the original rank from the single-omics level are given in Supplementary Figures S54–S58. Table 4 provides a summary of the top 20 GO-CC terms ranked based on the integrated score obtained from the transcriptomics and proteomics analyses. In addition, the table also includes the robustness rank difference of the GO term on the transcriptomics (robustness T) and proteomics (robustness P) levels. A lesser robustness rank difference indicates that the GO term is more robust across the transcriptomics and proteomics data, while a higher robustness rank difference indicates that the GO term is less robust (refer to Supplementary Table S2 (CC) for complete data).

**TABLE 4:** Top 20 GO terms of cellular components ranked based on the integrated score from the bootstrap analysis. The original ranks of each omics level (original rank T and original rank P), aggregated bootstrap rank (bootstrap rank T and bootstrap rank P), integrated score (the score obtained from common GO terms between omics levels), and robustness (gain T and gain P) of the term (either gain, loss, or the same rank compared with the ranks of the original analysis and the bootstrap analysis). Negative numbers indicate rank loss, and positive numbers indicate rank gain, while zero indicates no rank change.

GO ID	Term	Orig. rank T	Bt. rank T	Orig. rank P	Bt. rank P	Integrated score	Gain T	Gain P
GO:0000109	Nucleotide excision repair complex	202	37	1	2	0.0007	165	−1
GO:0000153	Cytoplasmic ubiquitin ligase complex	217	41	68	62	0.0019	176	6
GO:0016021	Integral component of the membrane	43	1	277	252	0.0020	42	25
GO:0005914	Spot adherens junction	503	1,228	2	1	0.0020	−725	1
GO:0000124	SAGA complex	209	27	101	65	0.0021	182	36
GO:0000813	ESCRT I complex	278	82	76	64	0.0033	196	12
GO:0031224	Intrinsic component of the membrane	50	2	263	244.5	0.0039	48	18.5
GO:0001527	Microfibril	298	94	69	47	0.0043	204	22
GO:0001533	Cornified envelope	299	95	117	87	0.0044	204	30
GO:0000974	Prp19 complex	293	90	107	103	0.0052	203	4
GO:0000315	Organellar large ribosomal subunit	239	51	113	110	0.0059	188	3
GO:0012505	Endomembrane system	59	3	397	371	0.0059	56	26
GO:0097229	Sperm end piece	1,578	1,768	3	3	0.0059	−190	0
GO:0001917	Photoreceptor inner segment	315	116	36	60	0.0066	199	−24
GO:0002178	Palmitoyltransferase complex	334	125	49	49	0.0076	209	0
GO:0071944	Cell periphery	86	4	353	319.5	0.0079	82	33.5
GO:0098965	Extracellular matrix of the synaptic cleft	1,733	1,768	4	4	0.0079	−35	0
GO:0001750	Photoreceptor outer segment	312	110	167	128	0.0080	202	39
GO:0000835	ER ubiquitin ligase complex	282	130	28	23	0.0082	152	5
GO:0000836	Hrd1p ubiquitin ligase complex	283	132	29	25	0.0084	151	4

These results highlight the importance of analyzing the robustness of GSEA to ensure that the results are reliable and reproducible. By identifying the GO-CC terms that are most robust across different omics levels, researchers can gain a more comprehensive understanding of the biological processes involved in the studied system.

Finally, we compared the ranks obtained from the transcriptomics level with those from the proteomics level separately for the original results and bootstrap results. For the GO categories, we found that transcriptomics and proteomics ranks from the bootstrap analyses are more correlated than the related ranks from the original analysis (BP: Kendall’s *τ*_*orig*._ = 0.29 and *τ*_*boot*_ = 0.56; MF: *τ*_*orig*._ = 0.25 and *τ*_*boot*_ = 0.49; and CC: *τ*_*orig*._ = 0.33 and *τ*_*boot*_ = 0.53). For GSEA results with KEGG, Reactome, and WikiPathways databases, no significant correlation between the proteomics and transcriptomics levels was found.

## 4 Discussion

Set-based enrichment tests are a necessary part of omics data analyses to better understand the biological meaning of differentially expressed genes, proteins, or other molecules. However, because the set compositions provided in the databases are subject to uncertainties, incorrect pathways can emerge from the analysis and lead to biological interpretations in the wrong direction. We observed in the two data examples of this work that pathways or terms obtained from several independent datasets of the same omics domain or different omics domains can have only a moderate overlap. For example, in the SMA dataset, some pathways or GO terms were selected from either transcriptomics or proteomics data analysis. Here, cellular component terms ordered by their integrated score comprise of complex and subunits of complex terms that are indirectly related to neuromuscular diseases. The SMN is a part of the SMN complex and interferes functionally with several other complexes, such as the cytoplasmic ubiquitin ligase complex (GO:0000153) or ER ubiquitin ligase complex (GO:0000835) (Chaytow et al., 2018), which are highly ranked when ordered by their integrated score but were ranked much lower using the original ranking (Table 4). Furthermore, the top GO-BP term (Supplementary Table S2 (BP)) with the highest integrated rank (GO:0000245, spliceosomal complex assembly) has been associated with SMA (Price et al., 2018) but was ranked much lower with the standard GSEA of the single-omics analyses (transcriptomics rank: 1,596; proteomics rank: 81). Furthermore, vitamins play, in general, an important role in neurodegenerative disorders. Lack of water-soluble vitamins (GO:0006767, water-soluble vitamin metabolic process, integrated rank: 6) (Supplementary Table S2 (BP)) can lead to neurological diseases (Rai et al., 2021). In particular, vitamin B6 (GO:0042816, vitamin B6 metabolic process, integrated rank: 2) is necessary for the production of various neurotransmitters such as serotonin, dopamine, and *γ*-aminobutyric acid (GABA). Deficiencies in vitamin B6 have been linked to depression and impaired brain function, such as epilepsy (Hellmann and Mooney, 2010). This suggests that it might be beneficial to consider providing sufficient supplementation of nutrients involved in maintaining an optimal methylation state, including folic acid, vitamin B12, and vitamin B6, for individuals with SMA (Fitzgerald and McArdle, 1941; Friesen et al., 2001). Therefore, our new pipeline provides not only robust terms but also biologically relevant terms when ordered by their integrated score.

Several attempts have been made to obtain a more robust enrichment analysis, for example, by integrating information about pathway topology (Draghici et al., 2007; Glaab et al., 2010; Massa et al., 2010) or GO hierarchy (Alexa et al., 2006) into the algorithms. Other approaches use sample permutations (Subramanian et al., 2005) or comparisons with results for random gene sets (Kim and Volsky, 2005) to account for uncertainties. The GAGE method (Luo et al., 2009) improves the robustness of GSEA by treating curated gene sets as either a pathway or an experimentally derived differential expression set. A method to evaluate the contribution of individual features to the significance of a gene set was presented as well by Jung et al. (2011).

Here, we present a new approach that combines bootstrap analysis at the gene set level with rank aggregation. This approach accounts for the uncertainty in set compositions by repeatedly analyzing subsets of each gene set. The percentage of genes or proteins to be selected for bootstrap can be chosen by the user of our R package. In case the user assumes much uncertainty in the database, a lower percentage should be taken. A major advantage of this approach is that it can be easily combined with other GSEA approaches. Exemplarily, we demonstrated the combination of our approach with pathway and GO term enrichment analyses implemented using the R packages clusterProfiler and topGO. We showed that overlaps of the detected GO terms between independent datasets were larger when using the bootstrap approach instead of the ranking from the standard analysis (Figure 3; Supplementary Figure S36). Similarly, we showed a higher rank correlation for the detected GO terms from transcriptomics and proteomics in the SMA data. Although increased overlaps were not observed for all types of sets, overlaps obtained using the bootstrap analysis were in no case smaller than the overlaps from the standard analysis.

In contrast to other approaches to account for uncertainty in GSEA, the rank aggregation step of our pipeline also allows the combination of results from multiple datasets or omics domains. This could also improve the stability and reproducibility of the findings.

In contrast to the approach proposed by Schmid et al. (2016), we derived not only a measure for the robustness of the result for each set but also provided a new ranking of sets. The size of either a gain or loss can be used as a measure of robustness. While we used the 2.5% and 97.5% quantiles, the user is free to use other thresholds to identify sets with extreme gains or losses. Nevertheless, thresholds are useful to account for different numbers of sets in an analysis. When having an overall large number of sets, the values of the quantiles will be larger as well, meaning that larger gains or losses are allowed before flagging a gain or loss as extreme. A disadvantage of the current rank aggregation approach is that the new ranking is based on a score and not on a *p*-value. Therefore, it is a bit more difficult to specify a threshold for the selected sets.

To conclude, set-based analyses now have a long history of omics data analysis to facilitate the biological interpretation of selected features from differential expression analysis. However, the large number of different computational GSEA methods presented in the last two decades and the huge databases with pathway annotations provide an unmanageable number of possible results, and analysts may be conventional in their biological interpretations. Moreover, some entries in the databases may be less supported by experimental findings or by the literature than other entries. In this regard, our bootstrap approach can help separate less robust findings from more robust findings. The rank aggregation step can additionally help combine gene set results from multiple datasets of the same or different omics levels. In particular, a GO term or pathway is only highly ranked by the integrated score if there is evidence for the importance of a term or pathway from different omics levels. We demonstrated the use of our approach in an example with transcriptomics and proteomics data, but it could be extended by GSEA from other omics domains, such as metabolomics (Mahajan et al., 2024). The rank aggregation step also supports the idea of research synthesis, that is, integrating findings from different studies or data sources to obtain a higher level of scientific evidence. Our new pipeline bootGSEA is universal as it can be combined with the most common GSEA methods. However, when using “topGO” for GO analysis, which works in the sense of overrepresentation analysis, users must keep in mind that the results depend on thresholds for differentially expressed features.

As a future extension of our approach, we also consider to not only remove features of pathways but to also move features between pathways, which is also an action we observed in databases. This can, however, only be done using biological information about whether a pathway feature makes biological sense in a certain pathway.

## Data Availability

Publicly available datasets were analyzed in this study. The gene expression data of the cancer study can be publicly retrieved from the NCBI Gene Expression Omnibus database (https://www.ncbi.nlm.nih.gov/geo/) using the accession numbers GSE6344, GSE14762, GSE11024, GSE14994, GSE53757, and GSE15641. The data of the SMA study will be made available by the authors upon request.

## References

[B1] Ackermann M. Strimmer K. (2009). A general modular framework for gene set enrichment analysis. BMC Bioinforma. 10, 47–20. 10.1186/1471-2105-10-47 PMC2661051 19192285

[B2] Alexa A. Rahnenführer J. (2009). Gene set enrichment analysis with topgo. Bioconductor Improv 27, 1–26.

[B3] Alexa A. Rahnenführer J. Lengauer T. (2006). Improved scoring of functional groups from gene expression data by decorrelating go graph structure. Bioinformatics 22, 1600–1607. 10.1093/bioinformatics/btl140 16606683

[B4] Allardyce H. Kuhn D. Hernandez-Gerez E. Hensel N. Huang Y.-T. Faller K. (2020). Renal pathology in a mouse model of severe spinal muscular atrophy is associated with downregulation of glial cell-line derived neurotrophic factor (gdnf). Hum. Mol. Genet. 29, 2365–2378. 10.1093/hmg/ddaa126 32588893

[B5] Bayerlová M. Jung K. Kramer F. Klemm F. Bleckmann A. Beißbarth T. (2015). Comparative study on gene set and pathway topology-based enrichment methods. BMC Bioinforma. 16, 1–15. 10.1186/s12859-015-0751-5 PMC4618947 26489510

[B6] Beissbarth T. Speed T. P. (2004). Gostat: find statistically overrepresented gene ontologies within a group of genes. Bioinformatics 20, 1464–1465. 10.1093/bioinformatics/bth088 14962934

[B7] Bolstad B. M. Irizarry R. A. Åstrand M. Speed T. P. (2003). A comparison of normalization methods for high density oligonucleotide array data based on variance and bias. Bioinformatics 19, 185–193. 10.1093/bioinformatics/19.2.185 12538238

[B8] Chaytow H. Huang Y.-T. Gillingwater T. H. Faller K. M. (2018). The role of survival motor neuron protein (smn) in protein homeostasis. Cell. Mol. Life Sci. 75, 3877–3894. 10.1007/s00018-018-2849-1 29872871 PMC6182345

[B9] Consortium G. O. (2004). The gene ontology (go) database and informatics resource. Nucleic acids Res. 32, D258–D261. 10.1093/nar/gkh036 14681407 PMC308770

[B10] Copland J. (2008). Transcription profiling of human stage 1,2 normal and tumor kidney cancer. Available at: https://www.ebi.ac.uk/biostudies/arrayexpress/studies/E-GEOD-6344.

[B11] Davis S. Meltzer P. (2007). Geoquery: a bridge between the gene expression omnibus (geo) and bioconductor. Bioinformatics 14, 1846–1847. 10.1093/bioinformatics/btm254 17496320

[B12] Draghici S. Khatri P. Tarca A. L. Amin K. Done A. Voichita C. (2007). A systems biology approach for pathway level analysis. Genome Res. 17, 1537–1545. 10.1101/gr.6202607 17785539 PMC1987343

[B13] Dykema K. Furge K. (2009). Renal cell carcinoma: hypoxia and endocytosis. Available at: https://www.ebi.ac.uk/biostudies/arrayexpress/studies/E-GEOD-14762.

[B14] Fabregat A. Sidiropoulos K. Garapati P. Gillespie M. Hausmann K. Haw R. (2016). The reactome pathway knowledgebase. Nucleic acids Res. 44, D481–D487. 10.1093/nar/gkv1351 26656494 PMC4702931

[B15] Fitzgerald G. McArdle B. (1941). Vitamins E and B6 in the treatment of muscular dystrophy and motor neurone disease. Brain 64, 19–42. 10.1093/brain/64.1.19

[B16] Friesen W. J. Massenet S. Paushkin S. Wyce A. Dreyfuss G. (2001). Smn, the product of the spinal muscular atrophy gene, binds preferentially to dimethylarginine-containing protein targets. Mol. Cell. 7, 1111–1117. 10.1016/s1097-2765(01)00244-1 11389857

[B17] Gillis J. Pavlidis P. (2013). Assessing identity, redundancy and confounds in gene ontology annotations over time. Bioinformatics 29, 476–482. 10.1093/bioinformatics/bts727 23297035 PMC3570208

[B18] Glaab E. Baudot A. Krasnogor N. Valencia A. (2010). Topogsa: network topological gene set analysis. Bioinformatics 26, 1271–1272. 10.1093/bioinformatics/btq131 20335277 PMC2859135

[B19] Goeman J. J. Van De Geer S. A. De Kort F. Van Houwelingen H. C. (2004). A global test for groups of genes: testing association with a clinical outcome. Bioinformatics 20, 93–99. 10.1093/bioinformatics/btg382 14693814

[B20] Jones J. Otu H. Spentzos D. Kolia S. Inan M. Beecken W. M. (2005). Gene signatures of progression and metastasis in renal cell cancer. Clinical cancer research 11 (16), 5730–5739. 16115910 10.1158/1078-0432.CCR-04-2225

[B21] Hellmann H. Mooney S. (2010). Vitamin b6: a molecule for human health? Molecules 15, 442–459. 10.3390/molecules15010442 20110903 PMC6257116

[B22] Hensel N. Cieri F. Santonicola P. Tapken I. Schüning T. Taiana M. (2021). Impairment of the neurotrophic signaling hub b-raf contributes to motoneuron degeneration in spinal muscular atrophy. Proc. Natl. Acad. Sci. 118, e2007785118. 10.1073/pnas.2007785118 33931501 PMC8106332

[B23] Hensel N. Raker V. Förthmann B. Buch A. Sodeik B. Pich A. (2020). The proteome and secretome of cortical brain cells infected with herpes simplex virus. Front. Neurology 11, 844. 10.3389/fneur.2020.00844 PMC7481480 32973653

[B24] Hensel N. Ratzka A. Brinkmann H. Klimaschewski L. Grothe C. Claus P. (2012). Analysis of the fibroblast growth factor system reveals alterations in a mouse model of spinal muscular atrophy. Plos one 7, e31202. 10.1371/journal.pone.0031202 22348054 PMC3278439

[B25] Hsieh-Li H. M. Chang J.-G. Jong Y.-J. Wu M.-H. Wang N. M. Tsai C. H. (2000). A mouse model for spinal muscular atrophy. Nat. Genet. 24, 66–70. 10.1038/71709 10615130

[B26] Hummel M. Meister R. Mansmann U. (2008). Globalancova: exploration and assessment of gene group effects. Bioinformatics 24, 78–85. 10.1093/bioinformatics/btm531 18024976

[B27] John Copland C. K. Christina von Roemeling Tun H. (2014). Gene array analysis of clear cell renal cell carcinoma tissue versus matched normal kidney tissue. Available at: https://www.ebi.ac.uk/biostudies/arrayexpress/studies/E-GEOD-53757.

[B28] Jung K. Becker B. Brunner E. Beißbarth T. (2011). Comparison of global tests for functional gene sets in two-group designs and selection of potentially effect-causing genes. Bioinformatics 27, 1377–1383. 10.1093/bioinformatics/btr152 21441576

[B29] Kanehisa M. Goto S. (2000). Kegg: kyoto encyclopedia of genes and genomes. Nucleic acids Res. 28, 27–30. 10.1093/nar/28.1.27 10592173 PMC102409

[B30] Kang M. Ko E. Mersha T. B. (2022). A roadmap for multi-omics data integration using deep learning. Briefings Bioinforma. 23, bbab454. 10.1093/bib/bbab454 PMC8769688 34791014

[B31] Kelder T. Van Iersel M. P. Hanspers K. Kutmon M. Conklin B. R. Evelo C. T. (2012). Wikipathways: building research communities on biological pathways. Nucleic acids Res. 40, D1301–D1307. 10.1093/nar/gkr1074 22096230 PMC3245032

[B32] Kim S.-Y. Volsky D. J. (2005). Page: parametric analysis of gene set enrichment. BMC Bioinforma. 6, 1–12. 10.1186/1471-2105-6-144 PMC1183189 15941488

[B33] Kolde R. Laur S. Adler P. Vilo J. (2012). Robust rank aggregation for gene list integration and meta-analysis. Bioinformatics 28, 573–580. 10.1093/bioinformatics/btr709 22247279 PMC3278763

[B34] Kort E. (2008). Microarray analaysis of adult and childhood renal tumors. Available at: https://www.ebi.ac.uk/biostudies/arrayexpress/studies/E-GEOD-11024.

[B35] Lefebvre S. Bürglen L. Reboullet S. Clermont O. Burlet P. Viollet L. (1995). Identification and characterization of a spinal muscular atrophy-determining gene. Cell. 80, 155–165. 10.1016/0092-8674(95)90460-3 7813012

[B36] Liberzon A. Subramanian A. Pinchback R. Thorvaldsdóttir H. Tamayo P. Mesirov J. P. (2011). Molecular signatures database (msigdb) 3.0. Bioinformatics 27, 1739–1740. 10.1093/bioinformatics/btr260 21546393 PMC3106198

[B37] Love M. Anders S. Huber W. (2014). Differential analysis of count data–the deseq2 package. Genome Biol. 15, 10–1186.

[B38] Luo W. Friedman M. S. Shedden K. Hankenson K. D. Woolf P. J. (2009). Gage: generally applicable gene set enrichment for pathway analysis. BMC Bioinforma. 10, 1–17. 10.1186/1471-2105-10-161 PMC2696452 19473525

[B39] Mahajan P. Fiehn O. Barupal D. (2024). Idsl. Goa: gene ontology analysis for interpreting metabolomic datasets. Sci. Rep. 14, 1299. 10.1038/s41598-024-51992-x 38221536 PMC10788336

[B40] Massa M. S. Chiogna M. Romualdi C. (2010). Gene set analysis exploiting the topology of a pathway. BMC Syst. Biol. 4, 1–15. 10.1186/1752-0509-4-121 20809931 PMC2945950

[B41] Price P. L. Morderer D. Rossoll W. (2018). Rnp assembly defects in spinal muscular atrophy. RNA Metabolism Neurodegener. Dis. 20, 143–171. 10.1007/978-3-319-89689-2_6 29916019

[B42] Rai S. N. Singh P. Steinbusch H. W. Vamanu E. Ashraf G. Singh M. P. (2021). The role of vitamins in neurodegenerative disease: an update. Biomedicines 9, 1284. 10.3390/biomedicines9101284 34680401 PMC8533313

[B43] R Core Team (2022). R: a language and environment for statistical computing. Vienna, Austria: R Foundation for Statistical Computing.

[B44] Riessland M. Ackermann B. Förster A. Jakubik M. Hauke J. Garbes L. (2010). Saha ameliorates the sma phenotype in two mouse models for spinal muscular atrophy. Hum. Mol. Genet. 19, 1492–1506. 10.1093/hmg/ddq023 20097677

[B45] Ritchie M. E. Phipson B. Wu D. Hu Y. Law C. W. Shi W. (2015). Limma powers differential expression analyses for rna-sequencing and microarray studies. Nucleic acids Res. 43, e47. 10.1093/nar/gkv007 25605792 PMC4402510

[B46] Schmid F. Schmid M. Müssel C. Sträng J. E. Buske C. Bullinger L. (2016). Giant: gene set uncertainty in enrichment analysis. Bioinformatics 32, 1891–1894. 10.1093/bioinformatics/btw030 26833345

[B47] Shannon P. Markiel A. Ozier O. Baliga N. S. Wang J. T. Ramage D. (2003). Cytoscape: a software environment for integrated models of biomolecular interaction networks. Genome Res. 13, 2498–2504. 10.1101/gr.1239303 14597658 PMC403769

[B48] Signoretti S. Beroukhim R. (2010). Patterns of gene expression and copy-number alterations in VHL disease-associated and sporadic ccRCC. Available at: https://www.ebi.ac.uk/biostudies/arrayexpress/studies/E-GEOD-14994. 10.1158/0008-5472.CAN-09-0146 PMC2745239 19470766

[B49] Subramanian A. Tamayo P. Mootha V. K. Mukherjee S. Ebert B. L. Gillette M. A. (2005). Gene set enrichment analysis: a knowledge-based approach for interpreting genome-wide expression profiles. Proc. Natl. Acad. Sci. 102, 15545–15550. 10.1073/pnas.0506580102 16199517 PMC1239896

[B50] Supek F. Bošnjak M. Škunca N. Šmuc T. (2011). Revigo summarizes and visualizes long lists of gene ontology terms. PloS one 6, e21800. 10.1371/journal.pone.0021800 21789182 PMC3138752

[B51] Välikangas T. Suomi T. Elo L. L. (2018). A comprehensive evaluation of popular proteomics software workflows for label-free proteome quantification and imputation. Briefings Bioinforma. 19, 1344–1355. 10.1093/bib/bbx054 PMC6291797 28575146

[B52] Wang B. Mezlini A. M. Demir F. Fiume M. Tu Z. Brudno M. (2014). Similarity network fusion for aggregating data types on a genomic scale. Nat. methods 11, 333–337. 10.1038/nmeth.2810 24464287

[B53] Wu T. Hu E. Xu S. Chen M. Guo P. Dai Z. (2021). Clusterprofiler 4.0: a universal enrichment tool for interpreting omics data. Innovation 2, 100141. 10.1016/j.xinn.2021.100141 34557778 PMC8454663

[B54] Xiong Q. Ancona N. Hauser E. R. Mukherjee S. Furey T. S. (2012). Integrating genetic and gene expression evidence into genome-wide association analysis of gene sets. Genome Res. 22, 386–397. 10.1101/gr.124370.111 21940837 PMC3266045

[B55] Zhao Y. Wong L. Goh W. W. B. (2020). How to do quantile normalization correctly for gene expression data analyses. Sci. Rep. 10, 15534. 10.1038/s41598-020-72664-6 32968196 PMC7511327

