# Supplementary material for: bootGSEA: a bootstrap and rank aggregation pipeline for multi-study and multi-omics enrichment analyses
Source: Front Bioinform. 2024 Apr 3;4:1380928. doi: 10.3389/fbinf.2024.1380928 (PMC11021641; doi:10.3389/fbinf.2024.1380928)
Supplement: Supplementary file 2 [file Presentation1.PPTX]

## Slide 1
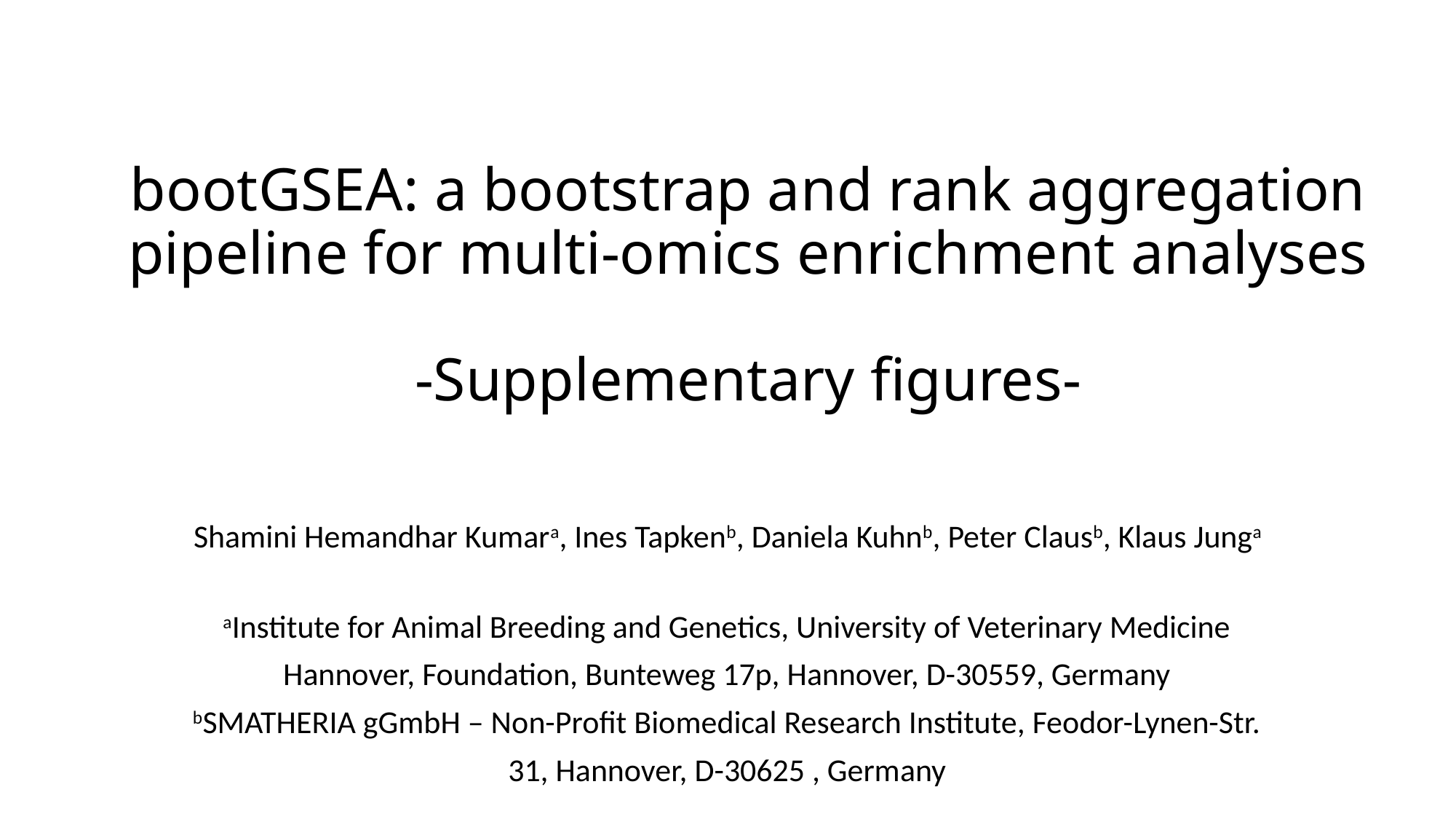

# bootGSEA: a bootstrap and rank aggregation pipeline for multi-omics enrichment analyses -Supplementary figures-
Shamini Hemandhar Kumara, Ines Tapkenb, Daniela Kuhnb, Peter Clausb, Klaus Junga
aInstitute for Animal Breeding and Genetics, University of Veterinary Medicine
Hannover, Foundation, Bunteweg 17p, Hannover, D-30559, Germany
bSMATHERIA gGmbH – Non-Profit Biomedical Research Institute, Feodor-Lynen-Str.
31, Hannover, D-30625 , Germany

## Slide 2
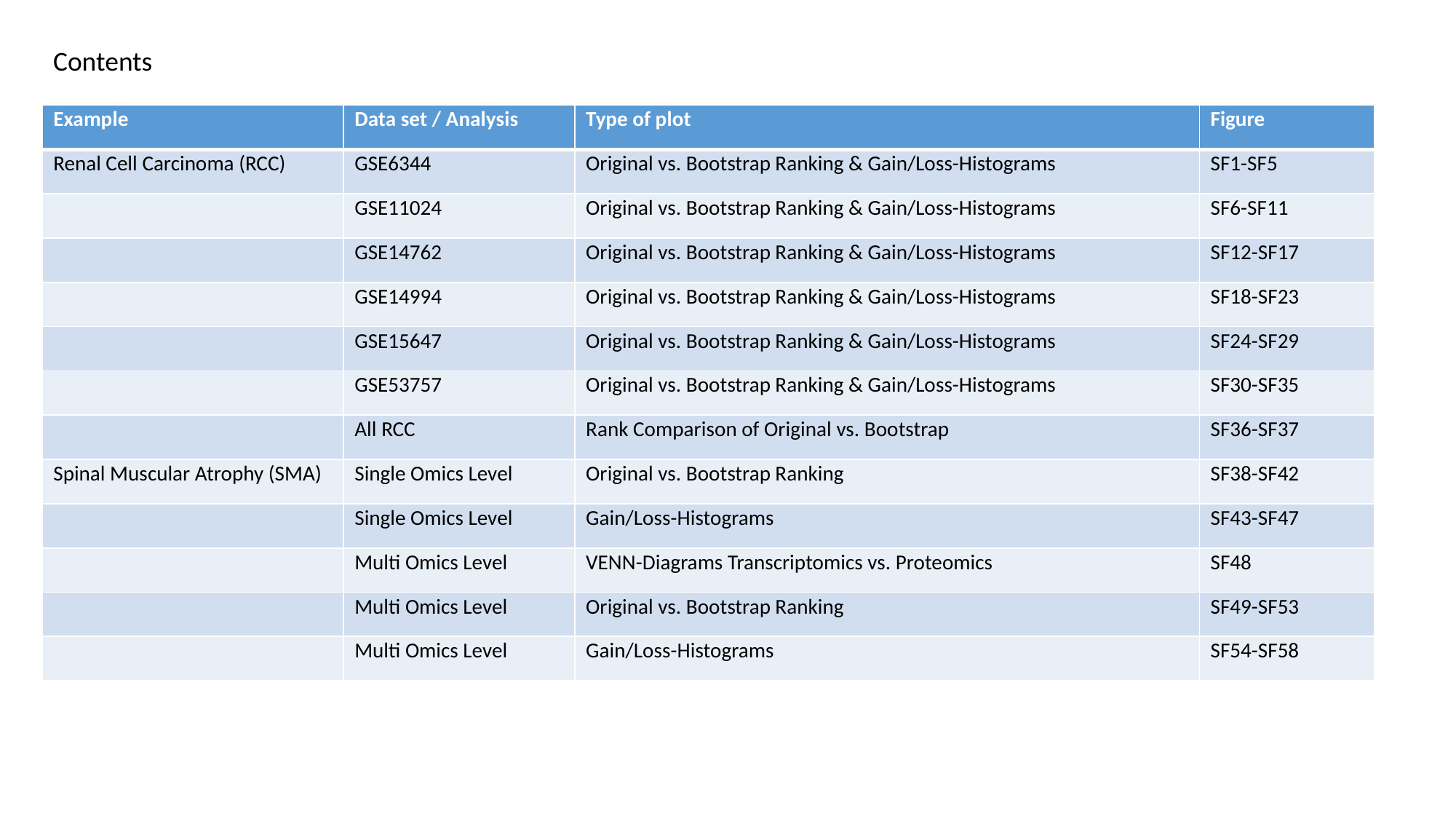

Contents
| Example | Data set / Analysis | Type of plot | Figure |
| --- | --- | --- | --- |
| Renal Cell Carcinoma (RCC) | GSE6344 | Original vs. Bootstrap Ranking & Gain/Loss-Histograms | SF1-SF5 |
| | GSE11024 | Original vs. Bootstrap Ranking & Gain/Loss-Histograms | SF6-SF11 |
| | GSE14762 | Original vs. Bootstrap Ranking & Gain/Loss-Histograms | SF12-SF17 |
| | GSE14994 | Original vs. Bootstrap Ranking & Gain/Loss-Histograms | SF18-SF23 |
| | GSE15647 | Original vs. Bootstrap Ranking & Gain/Loss-Histograms | SF24-SF29 |
| | GSE53757 | Original vs. Bootstrap Ranking & Gain/Loss-Histograms | SF30-SF35 |
| | All RCC | Rank Comparison of Original vs. Bootstrap | SF36-SF37 |
| Spinal Muscular Atrophy (SMA) | Single Omics Level | Original vs. Bootstrap Ranking | SF38-SF42 |
| | Single Omics Level | Gain/Loss-Histograms | SF43-SF47 |
| | Multi Omics Level | VENN-Diagrams Transcriptomics vs. Proteomics | SF48 |
| | Multi Omics Level | Original vs. Bootstrap Ranking | SF49-SF53 |
| | Multi Omics Level | Gain/Loss-Histograms | SF54-SF58 |

## Slide 3
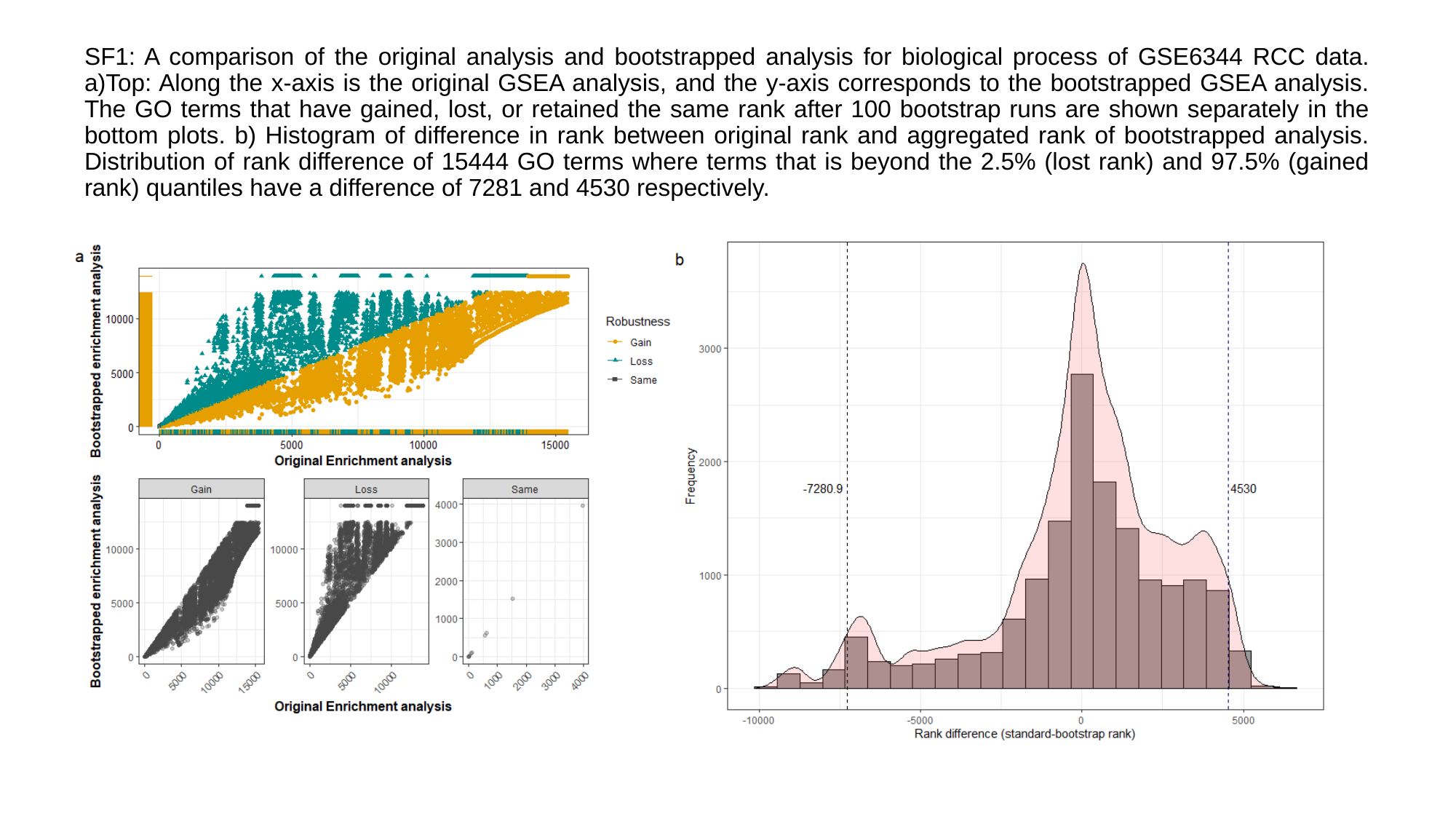

# SF1: A comparison of the original analysis and bootstrapped analysis for biological process of GSE6344 RCC data. a)Top: Along the x-axis is the original GSEA analysis, and the y-axis corresponds to the bootstrapped GSEA analysis. The GO terms that have gained, lost, or retained the same rank after 100 bootstrap runs are shown separately in the bottom plots. b) Histogram of difference in rank between original rank and aggregated rank of bootstrapped analysis. Distribution of rank difference of 15444 GO terms where terms that is beyond the 2.5% (lost rank) and 97.5% (gained rank) quantiles have a difference of 7281 and 4530 respectively.

## Slide 4
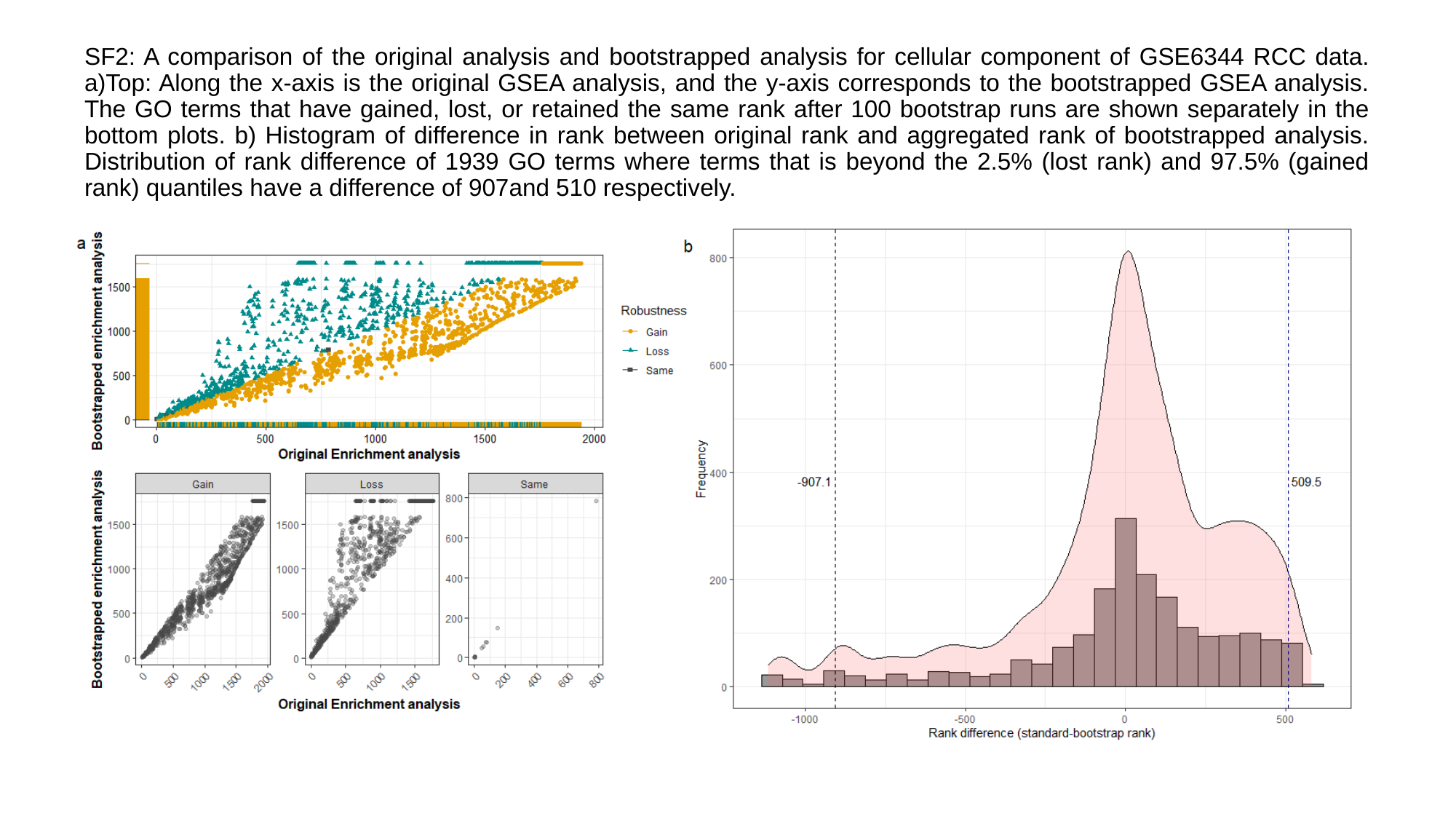

# SF2: A comparison of the original analysis and bootstrapped analysis for cellular component of GSE6344 RCC data. a)Top: Along the x-axis is the original GSEA analysis, and the y-axis corresponds to the bootstrapped GSEA analysis. The GO terms that have gained, lost, or retained the same rank after 100 bootstrap runs are shown separately in the bottom plots. b) Histogram of difference in rank between original rank and aggregated rank of bootstrapped analysis. Distribution of rank difference of 1939 GO terms where terms that is beyond the 2.5% (lost rank) and 97.5% (gained rank) quantiles have a difference of 907and 510 respectively.

## Slide 5
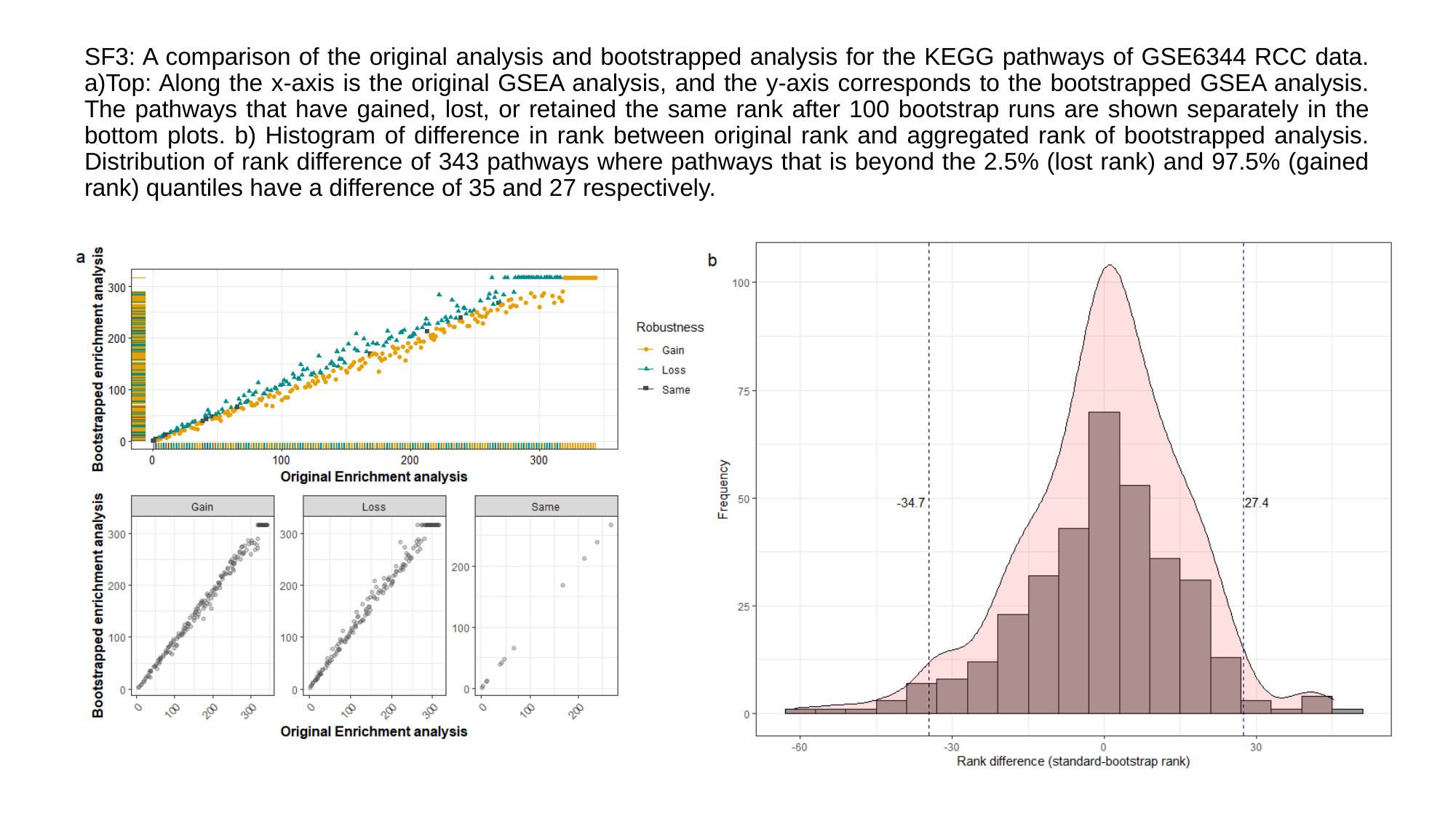

# SF3: A comparison of the original analysis and bootstrapped analysis for the KEGG pathways of GSE6344 RCC data. a)Top: Along the x-axis is the original GSEA analysis, and the y-axis corresponds to the bootstrapped GSEA analysis. The pathways that have gained, lost, or retained the same rank after 100 bootstrap runs are shown separately in the bottom plots. b) Histogram of difference in rank between original rank and aggregated rank of bootstrapped analysis. Distribution of rank difference of 343 pathways where pathways that is beyond the 2.5% (lost rank) and 97.5% (gained rank) quantiles have a difference of 35 and 27 respectively.

## Slide 6
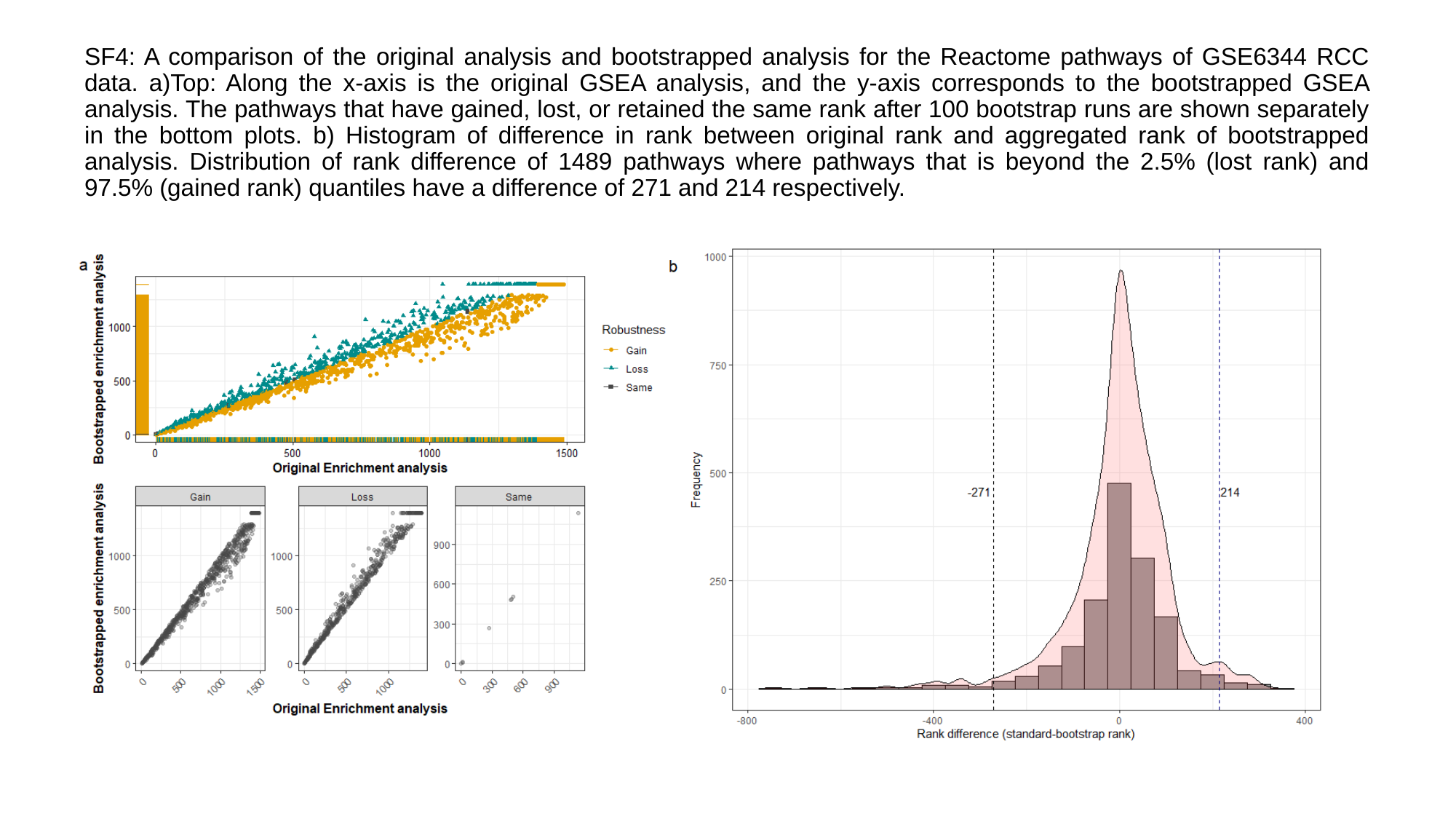

# SF4: A comparison of the original analysis and bootstrapped analysis for the Reactome pathways of GSE6344 RCC data. a)Top: Along the x-axis is the original GSEA analysis, and the y-axis corresponds to the bootstrapped GSEA analysis. The pathways that have gained, lost, or retained the same rank after 100 bootstrap runs are shown separately in the bottom plots. b) Histogram of difference in rank between original rank and aggregated rank of bootstrapped analysis. Distribution of rank difference of 1489 pathways where pathways that is beyond the 2.5% (lost rank) and 97.5% (gained rank) quantiles have a difference of 271 and 214 respectively.

## Slide 7
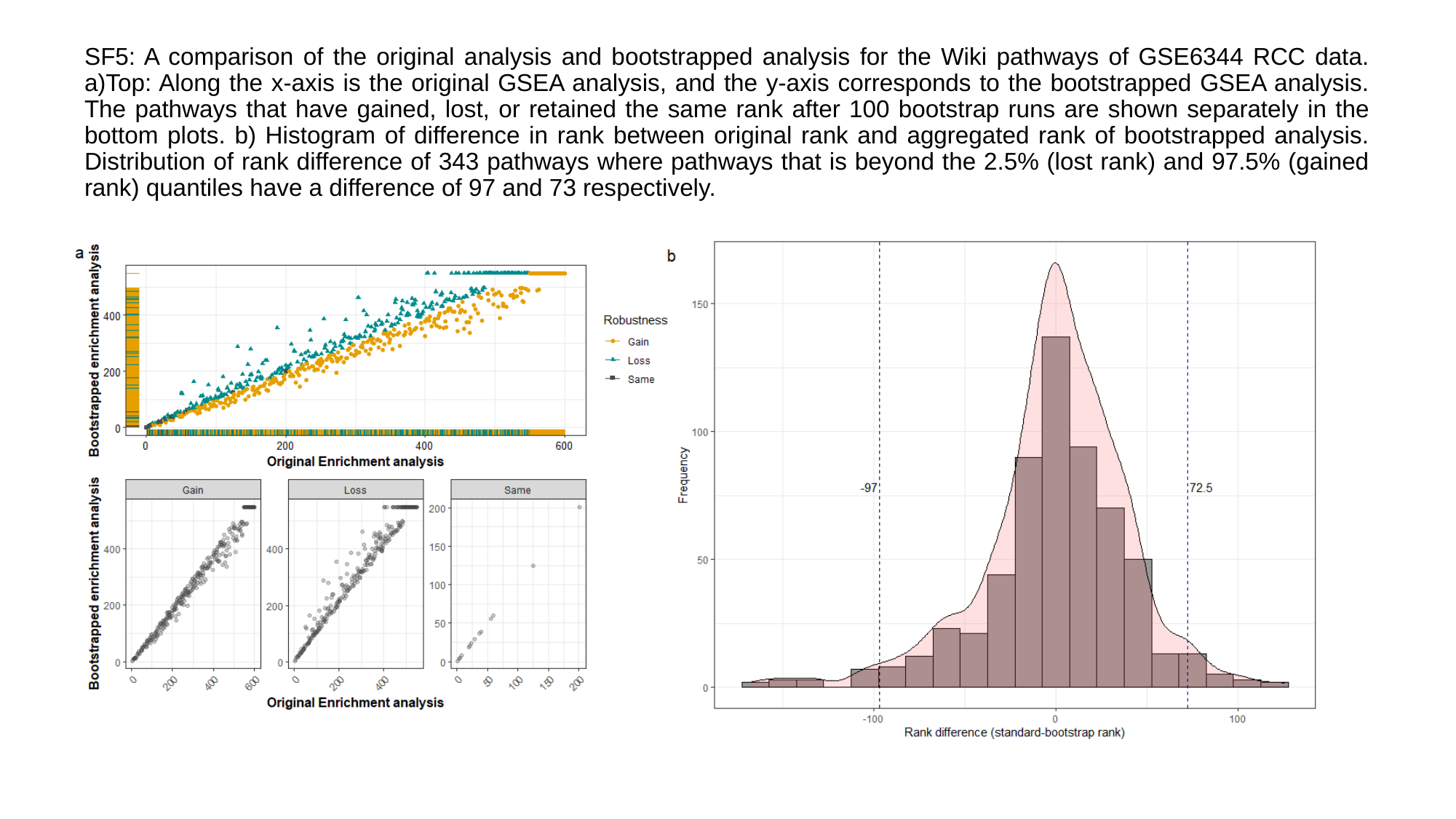

# SF5: A comparison of the original analysis and bootstrapped analysis for the Wiki pathways of GSE6344 RCC data. a)Top: Along the x-axis is the original GSEA analysis, and the y-axis corresponds to the bootstrapped GSEA analysis. The pathways that have gained, lost, or retained the same rank after 100 bootstrap runs are shown separately in the bottom plots. b) Histogram of difference in rank between original rank and aggregated rank of bootstrapped analysis. Distribution of rank difference of 343 pathways where pathways that is beyond the 2.5% (lost rank) and 97.5% (gained rank) quantiles have a difference of 97 and 73 respectively.

## Slide 8
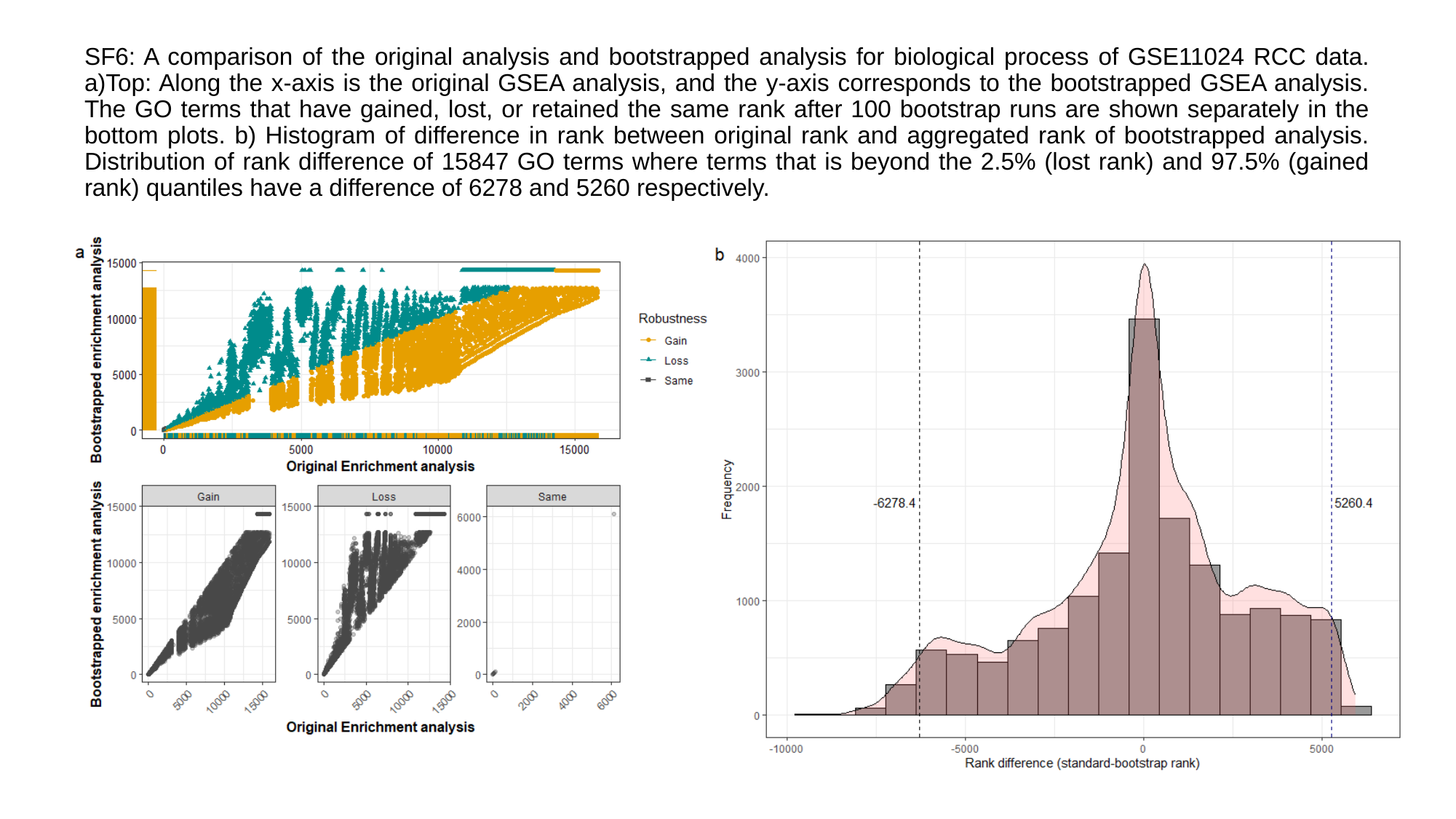

# SF6: A comparison of the original analysis and bootstrapped analysis for biological process of GSE11024 RCC data. a)Top: Along the x-axis is the original GSEA analysis, and the y-axis corresponds to the bootstrapped GSEA analysis. The GO terms that have gained, lost, or retained the same rank after 100 bootstrap runs are shown separately in the bottom plots. b) Histogram of difference in rank between original rank and aggregated rank of bootstrapped analysis. Distribution of rank difference of 15847 GO terms where terms that is beyond the 2.5% (lost rank) and 97.5% (gained rank) quantiles have a difference of 6278 and 5260 respectively.

## Slide 9
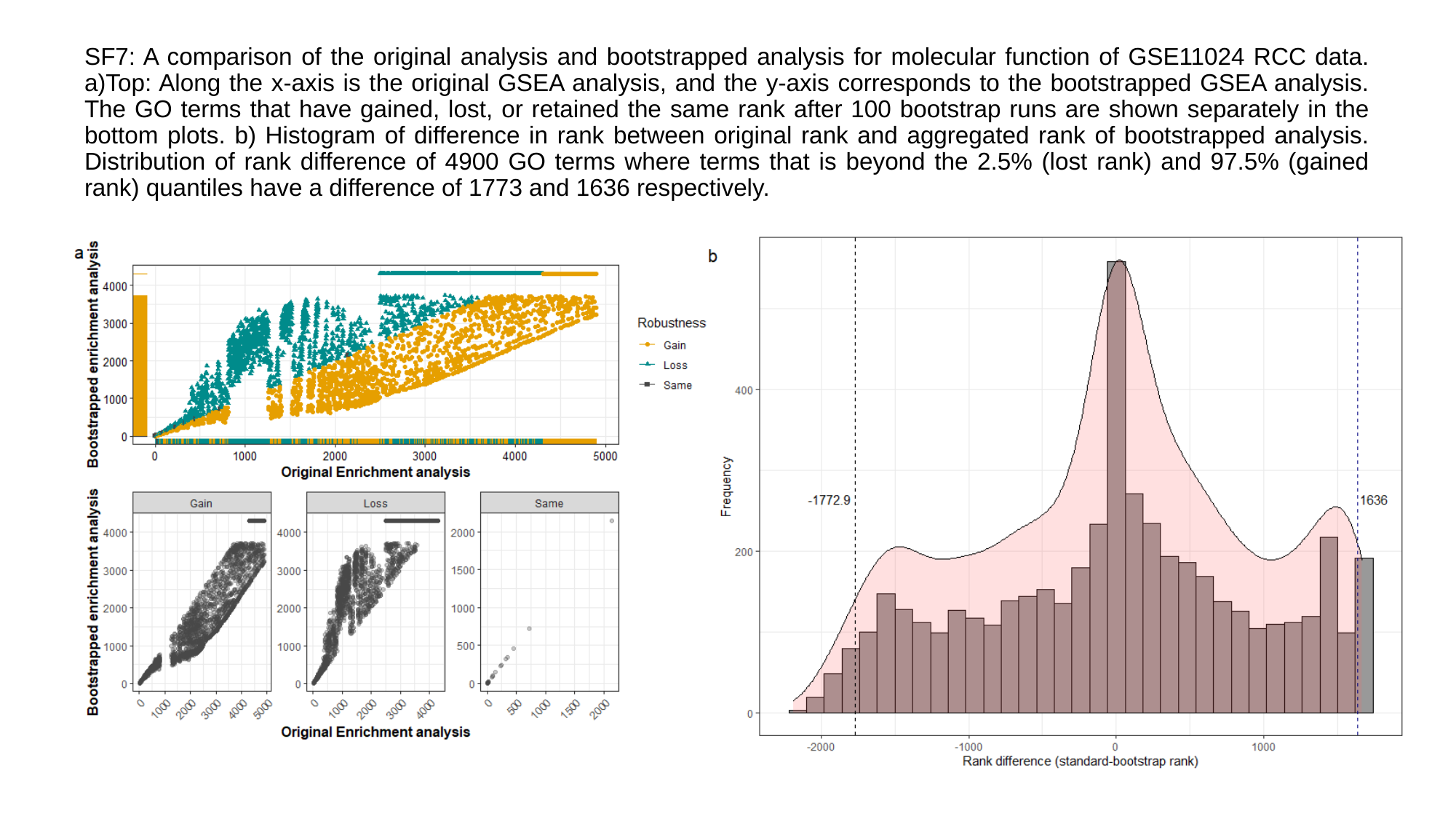

# SF7: A comparison of the original analysis and bootstrapped analysis for molecular function of GSE11024 RCC data. a)Top: Along the x-axis is the original GSEA analysis, and the y-axis corresponds to the bootstrapped GSEA analysis. The GO terms that have gained, lost, or retained the same rank after 100 bootstrap runs are shown separately in the bottom plots. b) Histogram of difference in rank between original rank and aggregated rank of bootstrapped analysis. Distribution of rank difference of 4900 GO terms where terms that is beyond the 2.5% (lost rank) and 97.5% (gained rank) quantiles have a difference of 1773 and 1636 respectively.

## Slide 10
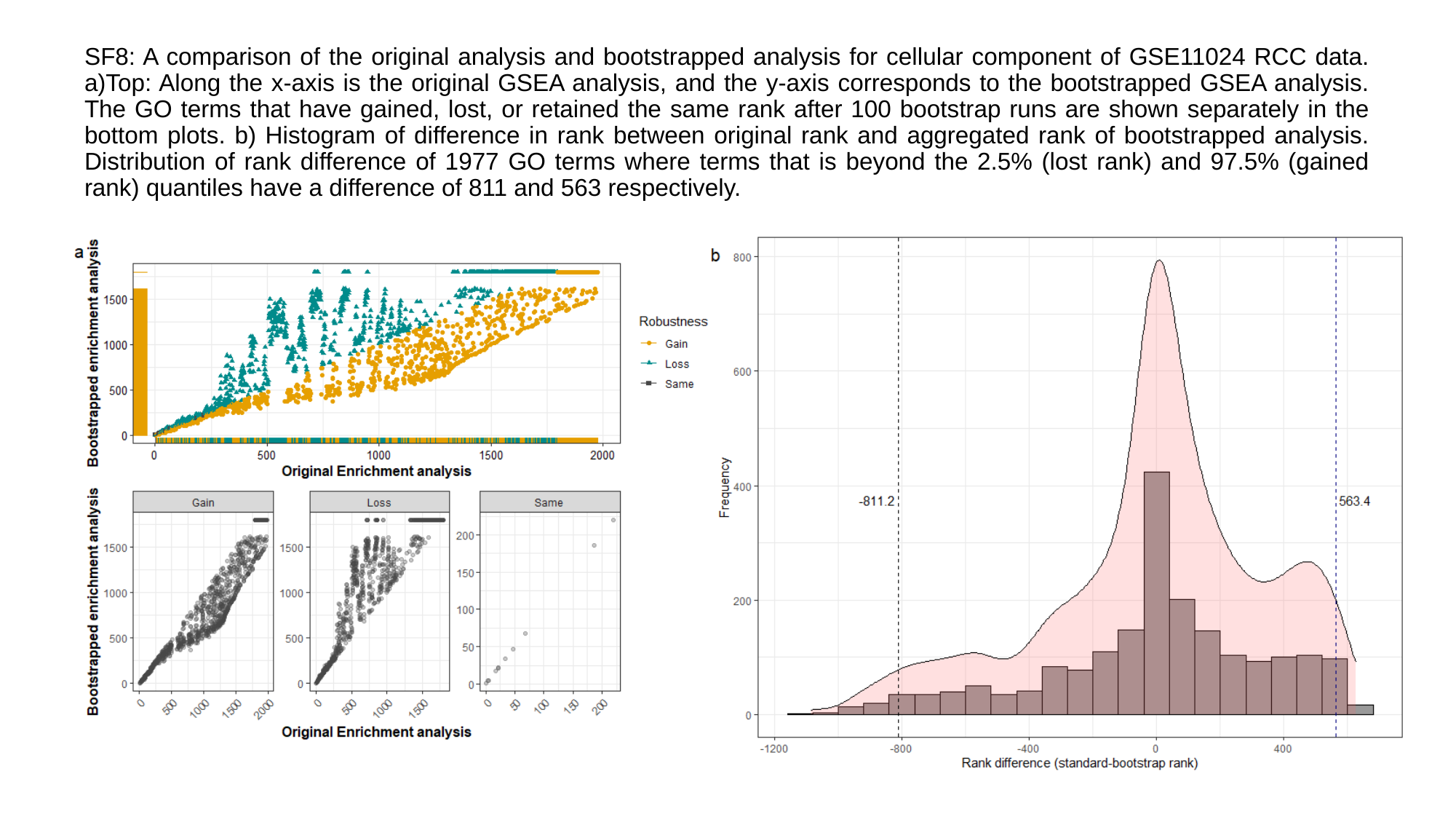

# SF8: A comparison of the original analysis and bootstrapped analysis for cellular component of GSE11024 RCC data. a)Top: Along the x-axis is the original GSEA analysis, and the y-axis corresponds to the bootstrapped GSEA analysis. The GO terms that have gained, lost, or retained the same rank after 100 bootstrap runs are shown separately in the bottom plots. b) Histogram of difference in rank between original rank and aggregated rank of bootstrapped analysis. Distribution of rank difference of 1977 GO terms where terms that is beyond the 2.5% (lost rank) and 97.5% (gained rank) quantiles have a difference of 811 and 563 respectively.

## Slide 11
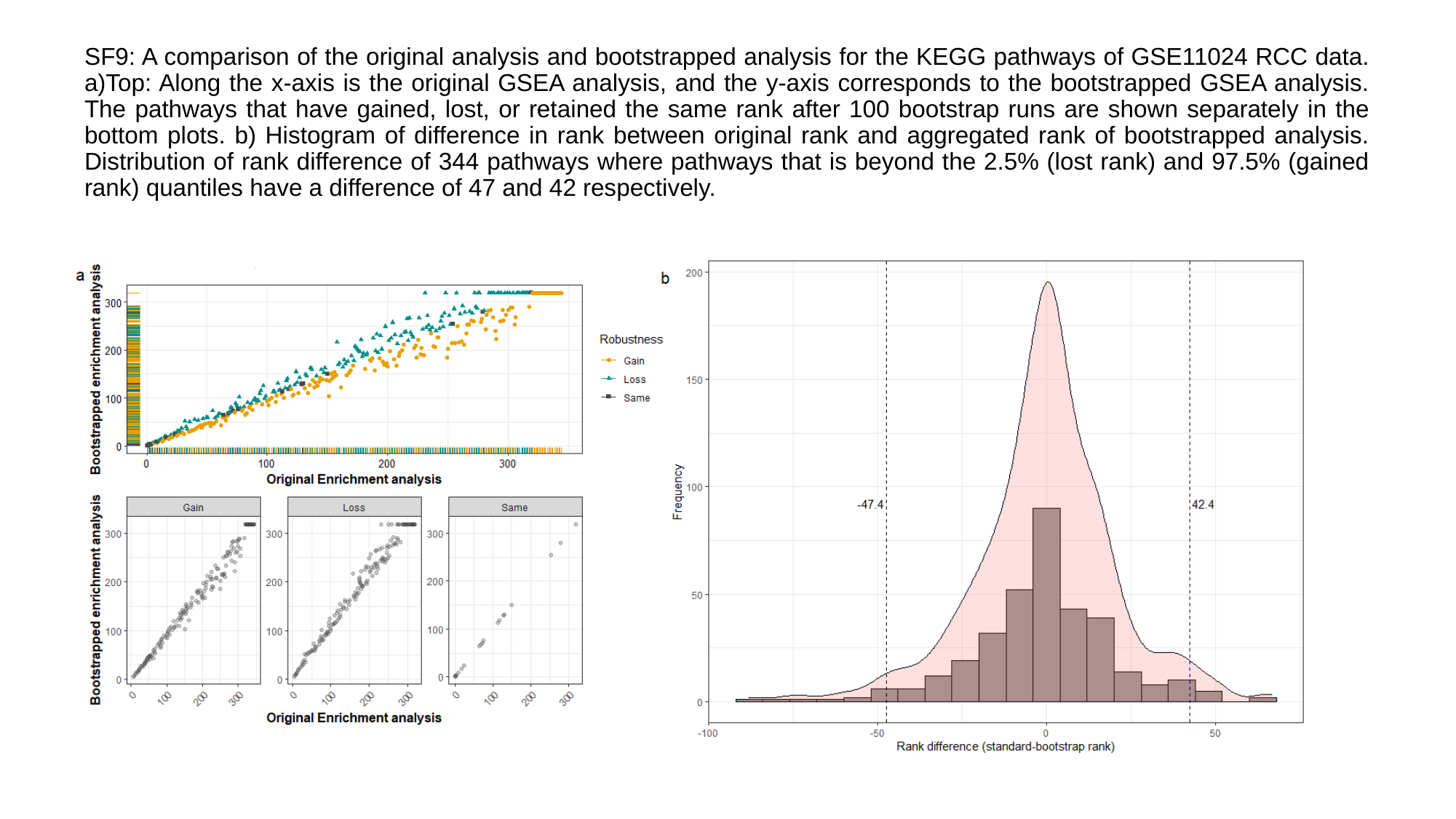

# SF9: A comparison of the original analysis and bootstrapped analysis for the KEGG pathways of GSE11024 RCC data. a)Top: Along the x-axis is the original GSEA analysis, and the y-axis corresponds to the bootstrapped GSEA analysis. The pathways that have gained, lost, or retained the same rank after 100 bootstrap runs are shown separately in the bottom plots. b) Histogram of difference in rank between original rank and aggregated rank of bootstrapped analysis. Distribution of rank difference of 344 pathways where pathways that is beyond the 2.5% (lost rank) and 97.5% (gained rank) quantiles have a difference of 47 and 42 respectively.

## Slide 12
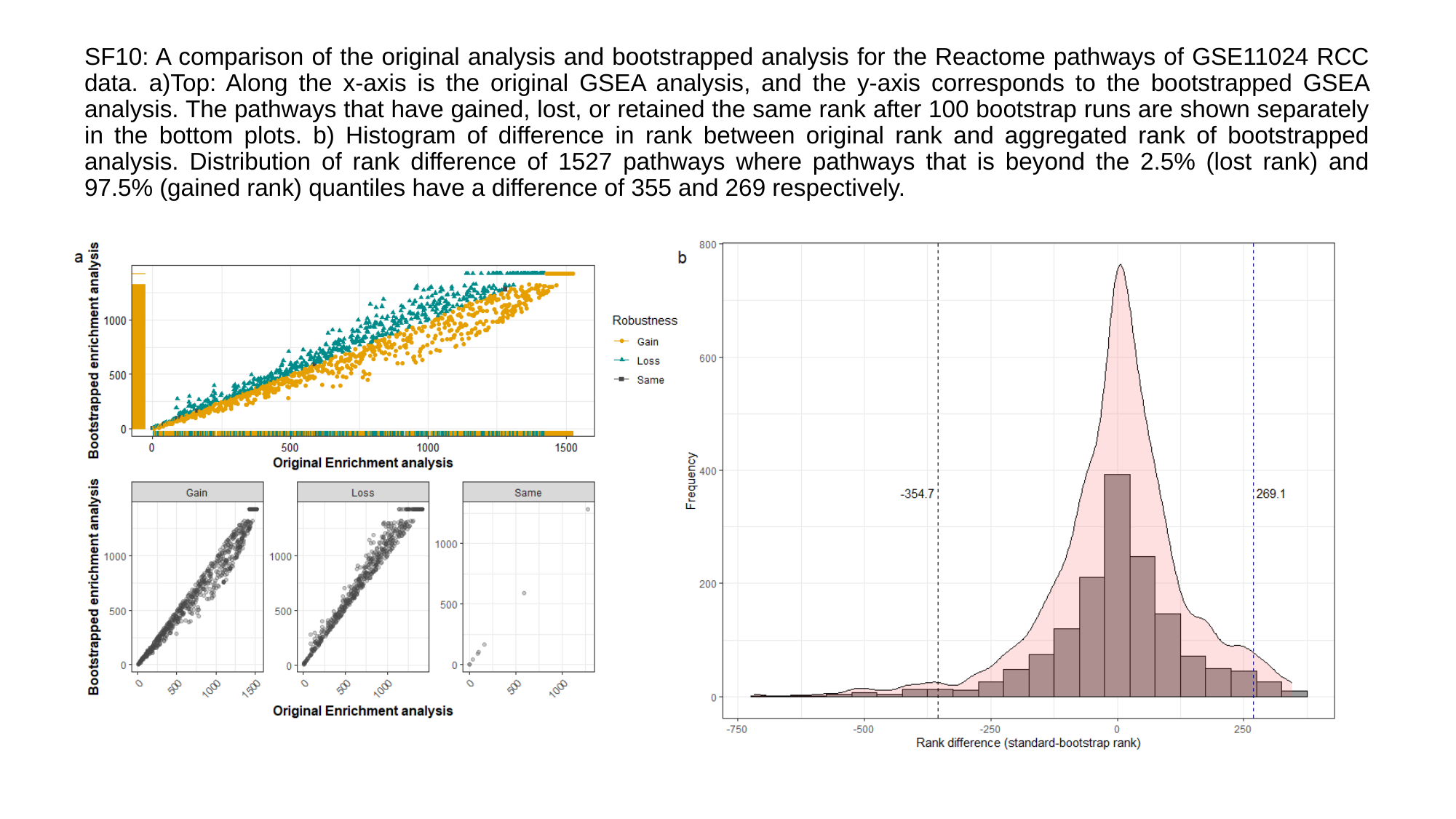

# SF10: A comparison of the original analysis and bootstrapped analysis for the Reactome pathways of GSE11024 RCC data. a)Top: Along the x-axis is the original GSEA analysis, and the y-axis corresponds to the bootstrapped GSEA analysis. The pathways that have gained, lost, or retained the same rank after 100 bootstrap runs are shown separately in the bottom plots. b) Histogram of difference in rank between original rank and aggregated rank of bootstrapped analysis. Distribution of rank difference of 1527 pathways where pathways that is beyond the 2.5% (lost rank) and 97.5% (gained rank) quantiles have a difference of 355 and 269 respectively.

## Slide 13
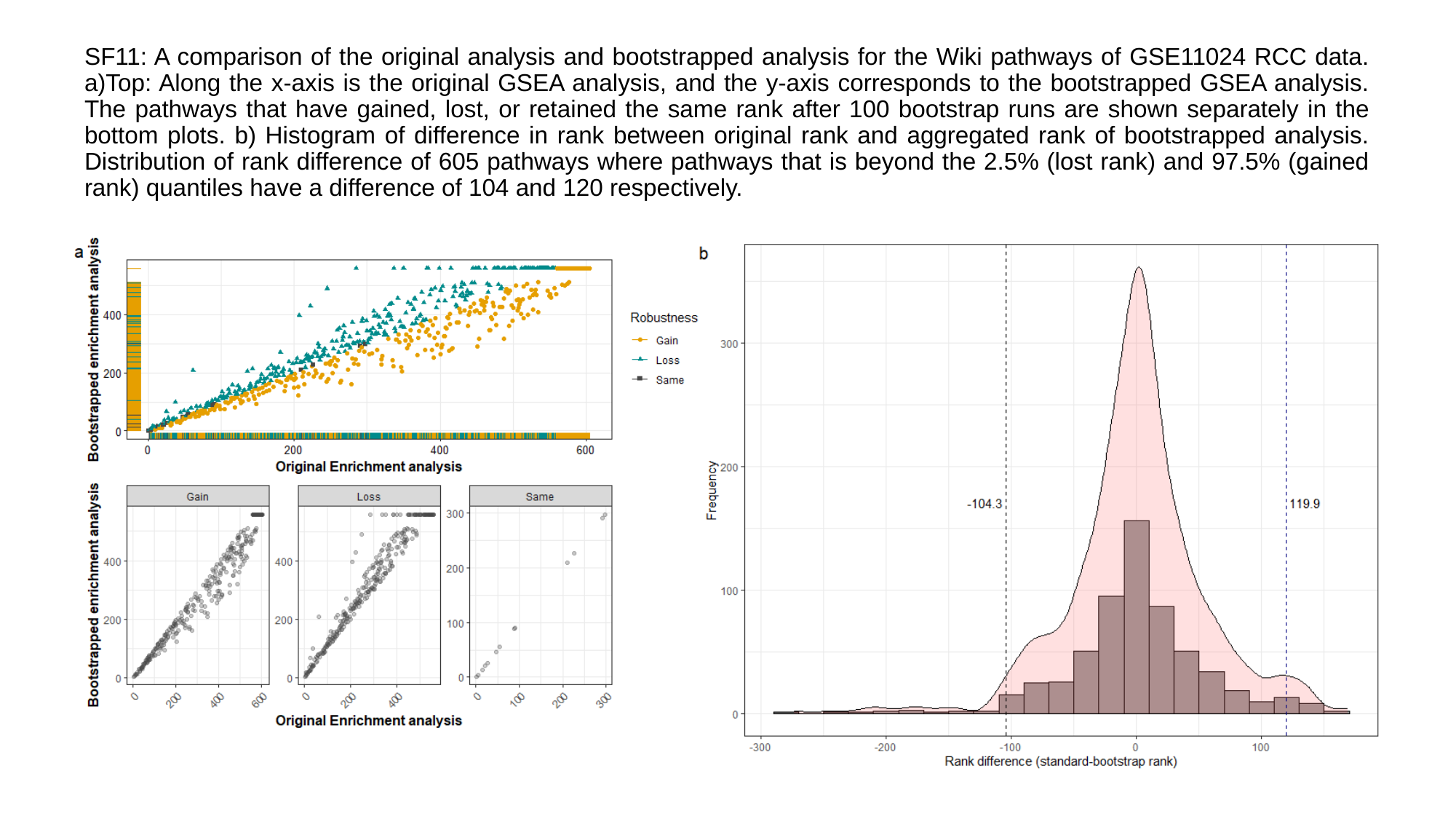

# SF11: A comparison of the original analysis and bootstrapped analysis for the Wiki pathways of GSE11024 RCC data. a)Top: Along the x-axis is the original GSEA analysis, and the y-axis corresponds to the bootstrapped GSEA analysis. The pathways that have gained, lost, or retained the same rank after 100 bootstrap runs are shown separately in the bottom plots. b) Histogram of difference in rank between original rank and aggregated rank of bootstrapped analysis. Distribution of rank difference of 605 pathways where pathways that is beyond the 2.5% (lost rank) and 97.5% (gained rank) quantiles have a difference of 104 and 120 respectively.

## Slide 14
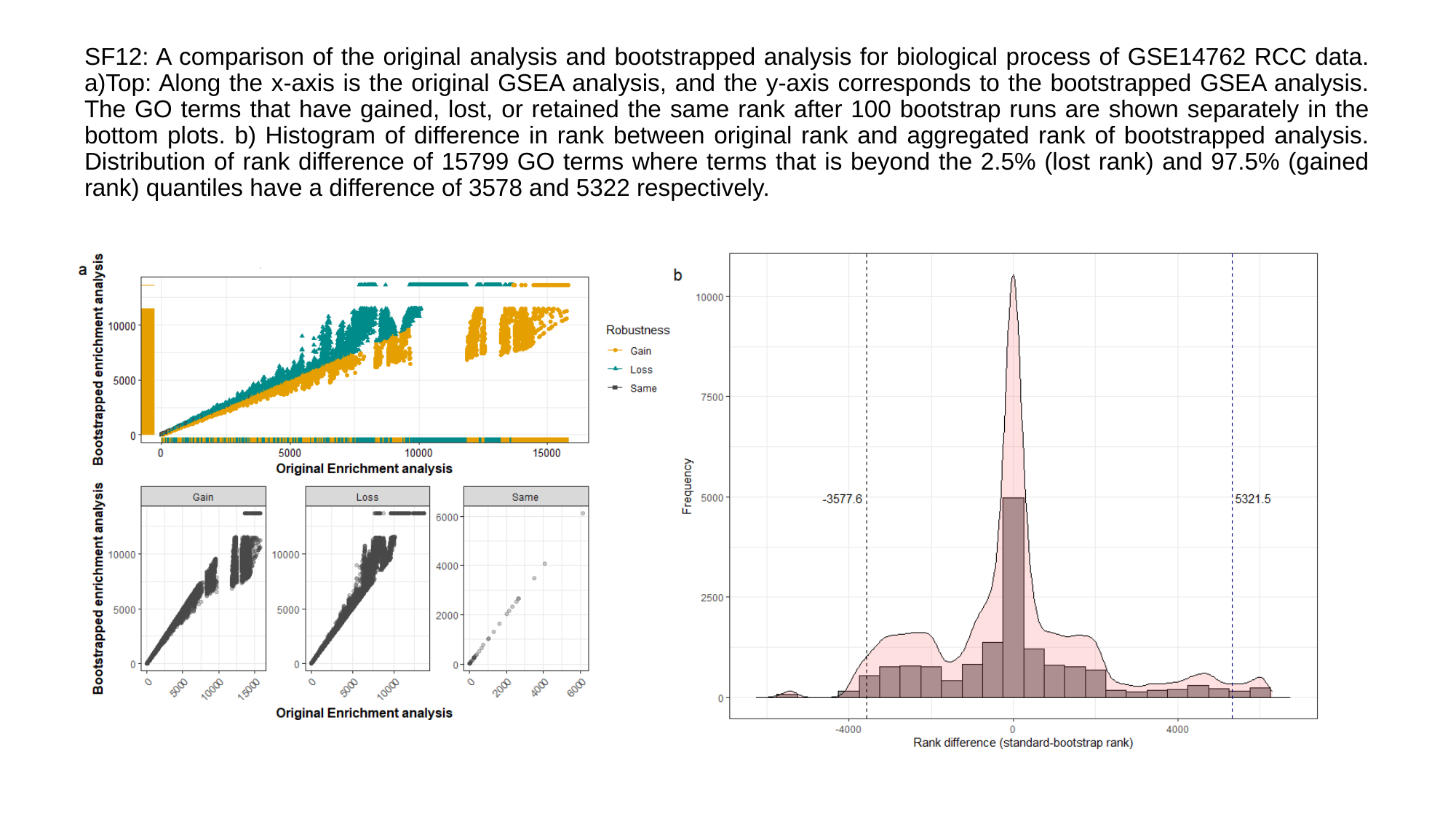

# SF12: A comparison of the original analysis and bootstrapped analysis for biological process of GSE14762 RCC data. a)Top: Along the x-axis is the original GSEA analysis, and the y-axis corresponds to the bootstrapped GSEA analysis. The GO terms that have gained, lost, or retained the same rank after 100 bootstrap runs are shown separately in the bottom plots. b) Histogram of difference in rank between original rank and aggregated rank of bootstrapped analysis. Distribution of rank difference of 15799 GO terms where terms that is beyond the 2.5% (lost rank) and 97.5% (gained rank) quantiles have a difference of 3578 and 5322 respectively.

## Slide 15
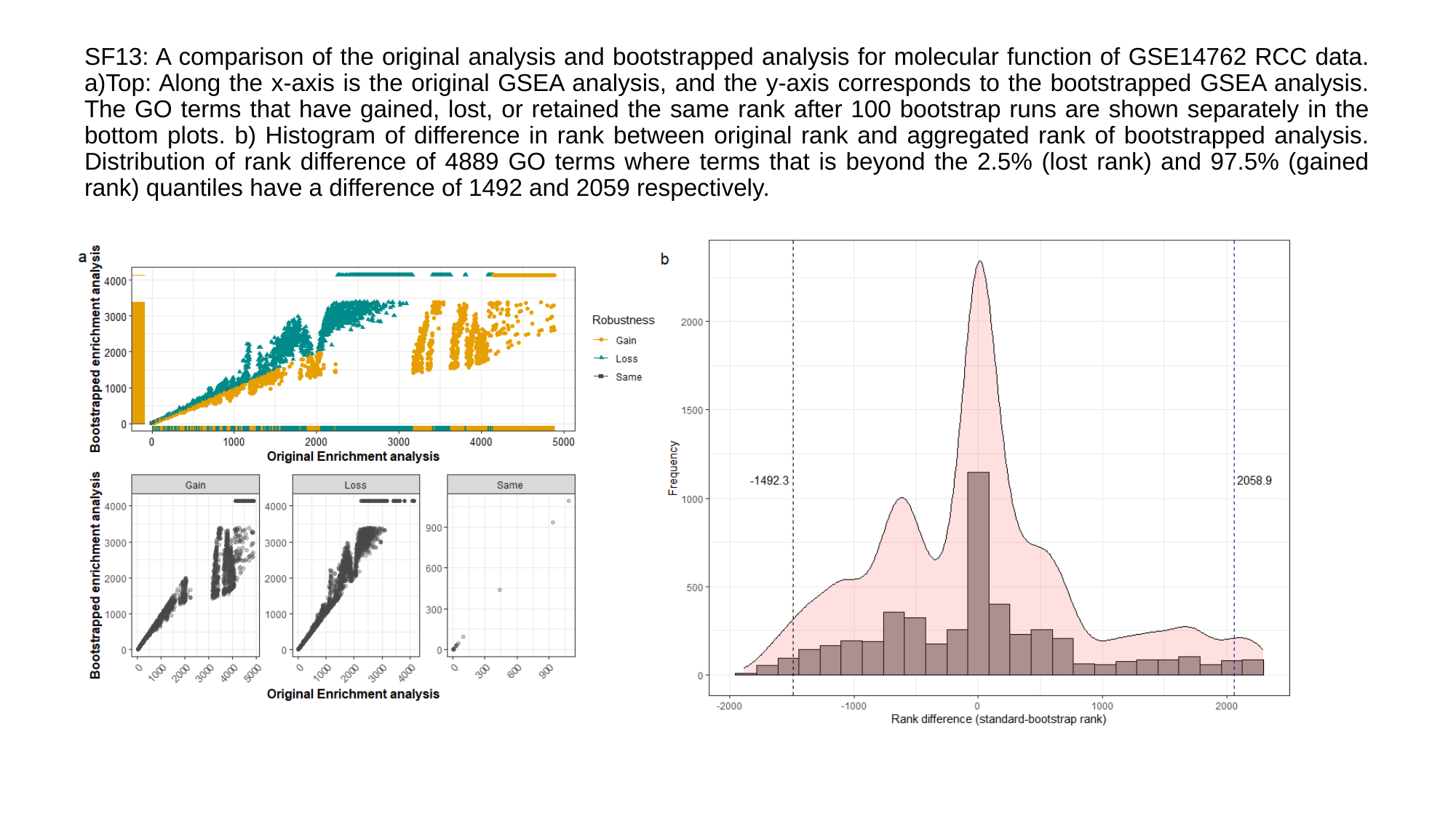

# SF13: A comparison of the original analysis and bootstrapped analysis for molecular function of GSE14762 RCC data. a)Top: Along the x-axis is the original GSEA analysis, and the y-axis corresponds to the bootstrapped GSEA analysis. The GO terms that have gained, lost, or retained the same rank after 100 bootstrap runs are shown separately in the bottom plots. b) Histogram of difference in rank between original rank and aggregated rank of bootstrapped analysis. Distribution of rank difference of 4889 GO terms where terms that is beyond the 2.5% (lost rank) and 97.5% (gained rank) quantiles have a difference of 1492 and 2059 respectively.

## Slide 16
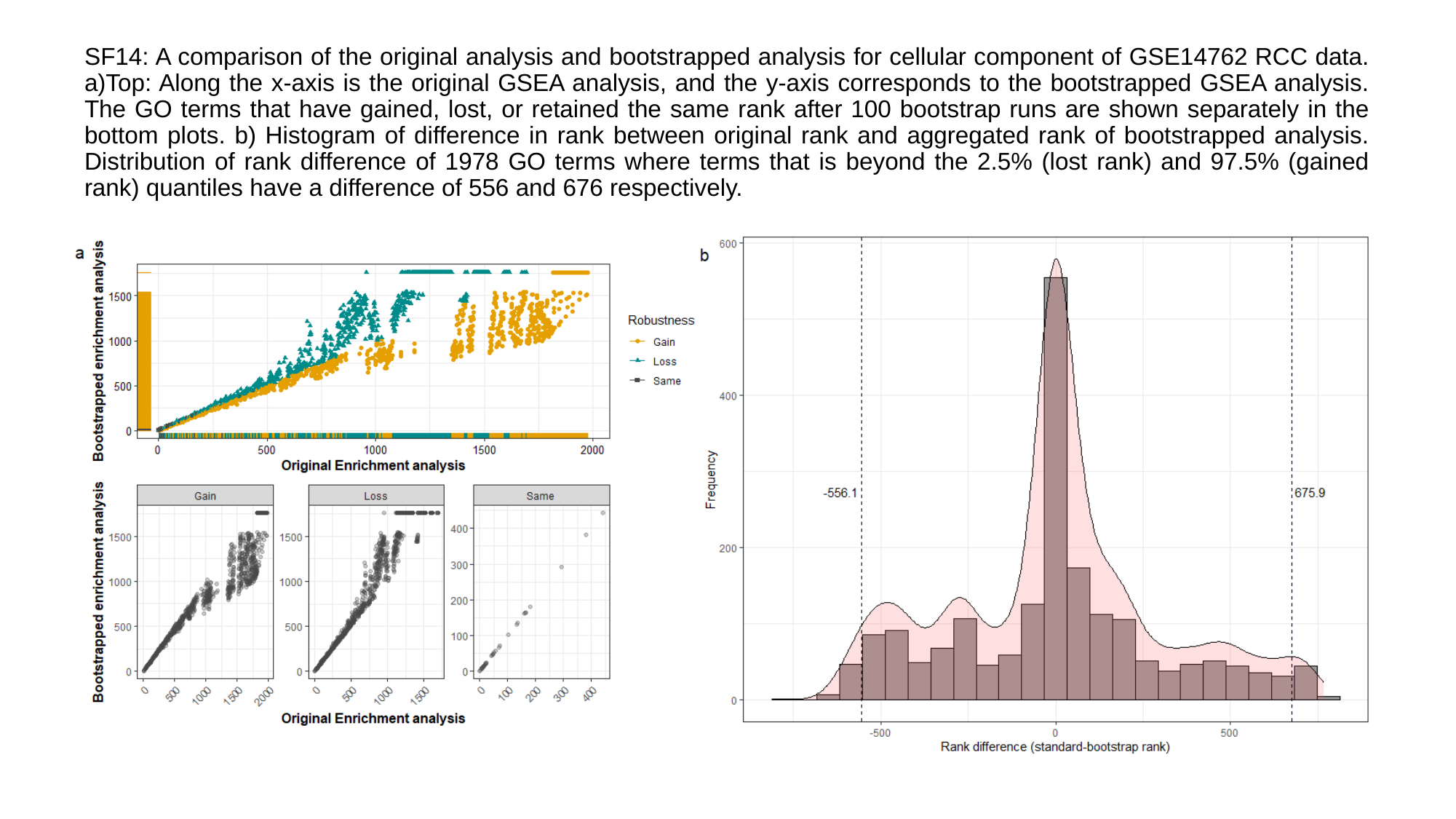

# SF14: A comparison of the original analysis and bootstrapped analysis for cellular component of GSE14762 RCC data. a)Top: Along the x-axis is the original GSEA analysis, and the y-axis corresponds to the bootstrapped GSEA analysis. The GO terms that have gained, lost, or retained the same rank after 100 bootstrap runs are shown separately in the bottom plots. b) Histogram of difference in rank between original rank and aggregated rank of bootstrapped analysis. Distribution of rank difference of 1978 GO terms where terms that is beyond the 2.5% (lost rank) and 97.5% (gained rank) quantiles have a difference of 556 and 676 respectively.

## Slide 17
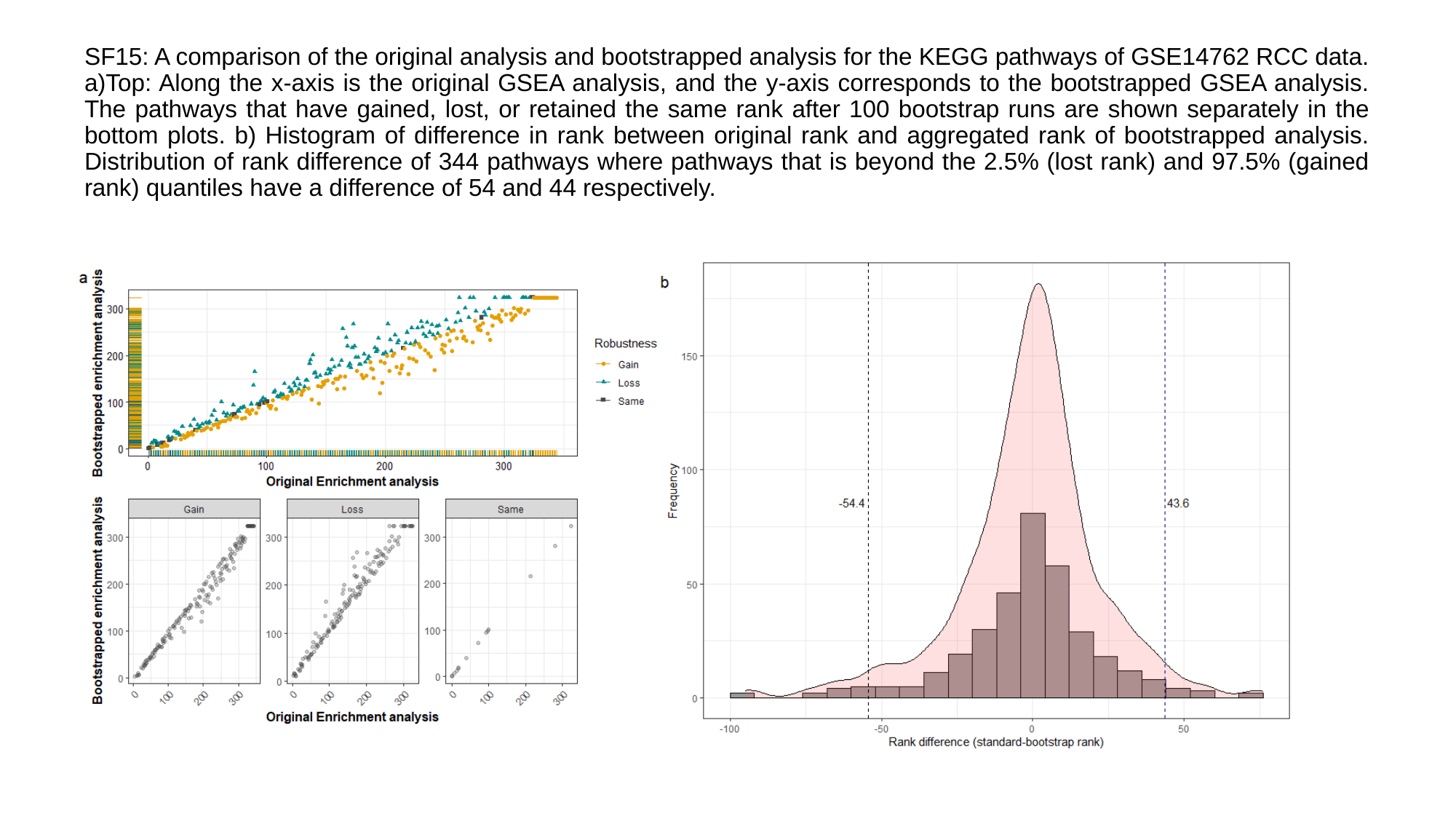

# SF15: A comparison of the original analysis and bootstrapped analysis for the KEGG pathways of GSE14762 RCC data. a)Top: Along the x-axis is the original GSEA analysis, and the y-axis corresponds to the bootstrapped GSEA analysis. The pathways that have gained, lost, or retained the same rank after 100 bootstrap runs are shown separately in the bottom plots. b) Histogram of difference in rank between original rank and aggregated rank of bootstrapped analysis. Distribution of rank difference of 344 pathways where pathways that is beyond the 2.5% (lost rank) and 97.5% (gained rank) quantiles have a difference of 54 and 44 respectively.

## Slide 18
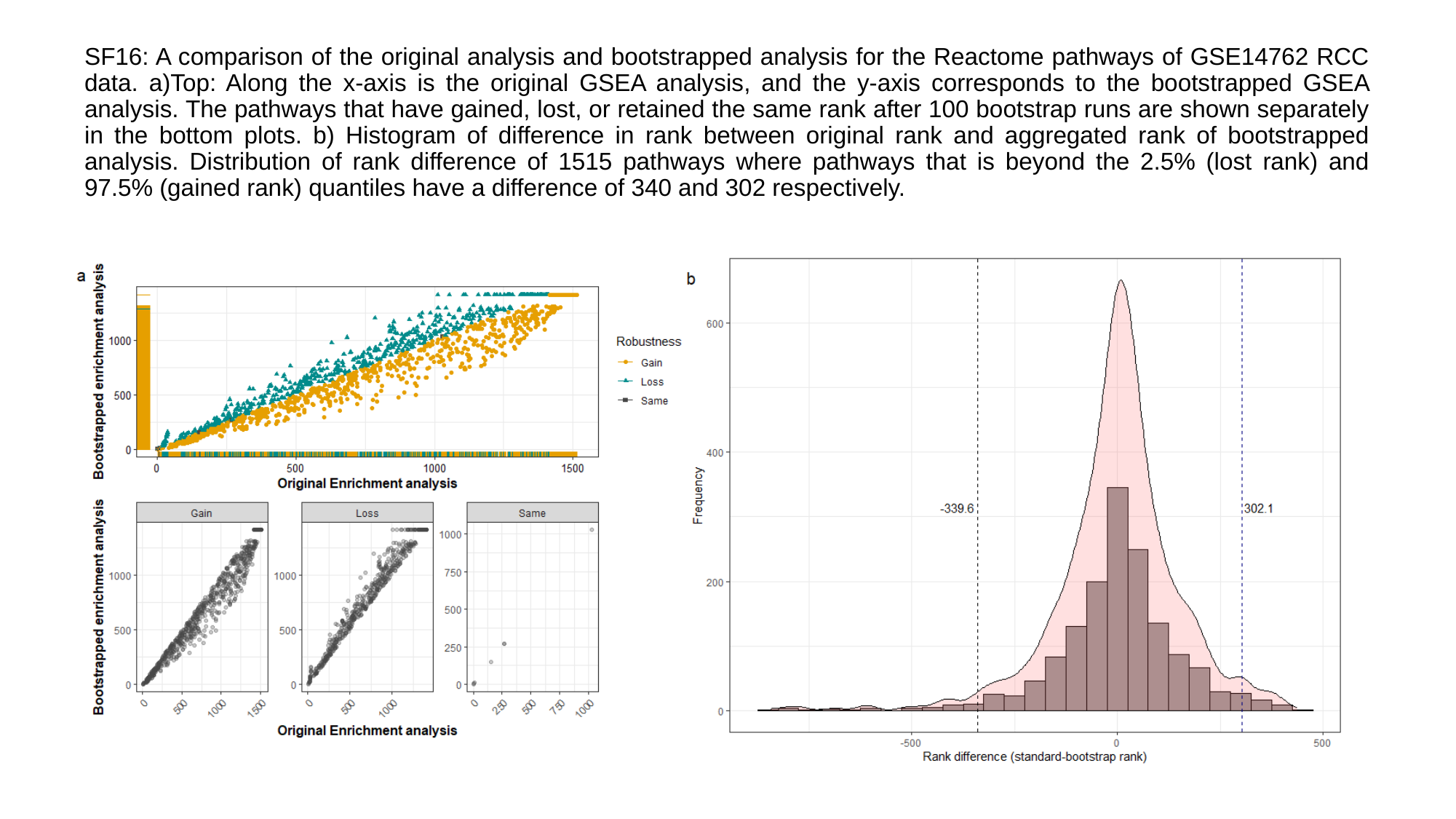

# SF16: A comparison of the original analysis and bootstrapped analysis for the Reactome pathways of GSE14762 RCC data. a)Top: Along the x-axis is the original GSEA analysis, and the y-axis corresponds to the bootstrapped GSEA analysis. The pathways that have gained, lost, or retained the same rank after 100 bootstrap runs are shown separately in the bottom plots. b) Histogram of difference in rank between original rank and aggregated rank of bootstrapped analysis. Distribution of rank difference of 1515 pathways where pathways that is beyond the 2.5% (lost rank) and 97.5% (gained rank) quantiles have a difference of 340 and 302 respectively.

## Slide 19
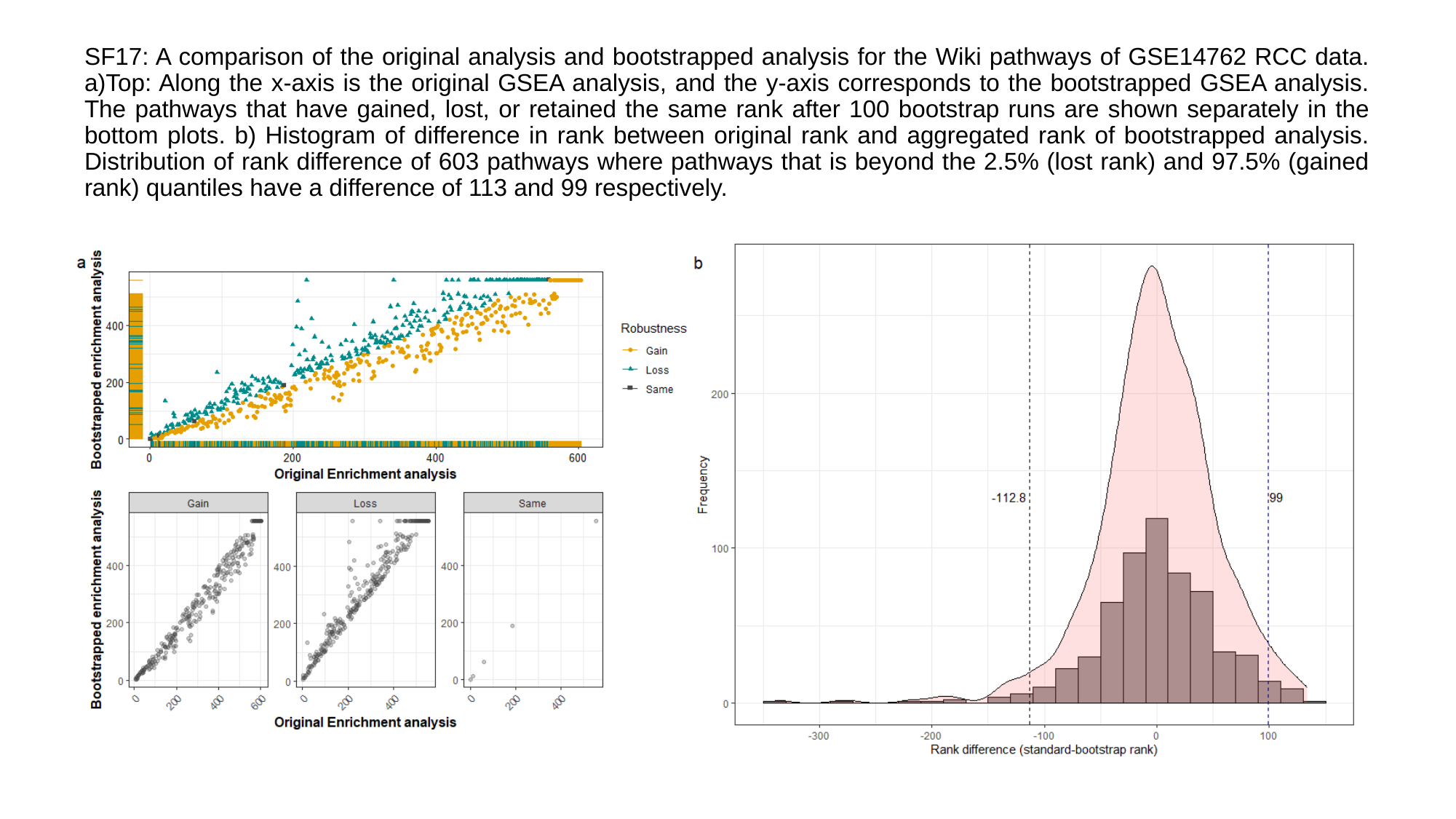

# SF17: A comparison of the original analysis and bootstrapped analysis for the Wiki pathways of GSE14762 RCC data. a)Top: Along the x-axis is the original GSEA analysis, and the y-axis corresponds to the bootstrapped GSEA analysis. The pathways that have gained, lost, or retained the same rank after 100 bootstrap runs are shown separately in the bottom plots. b) Histogram of difference in rank between original rank and aggregated rank of bootstrapped analysis. Distribution of rank difference of 603 pathways where pathways that is beyond the 2.5% (lost rank) and 97.5% (gained rank) quantiles have a difference of 113 and 99 respectively.

## Slide 20
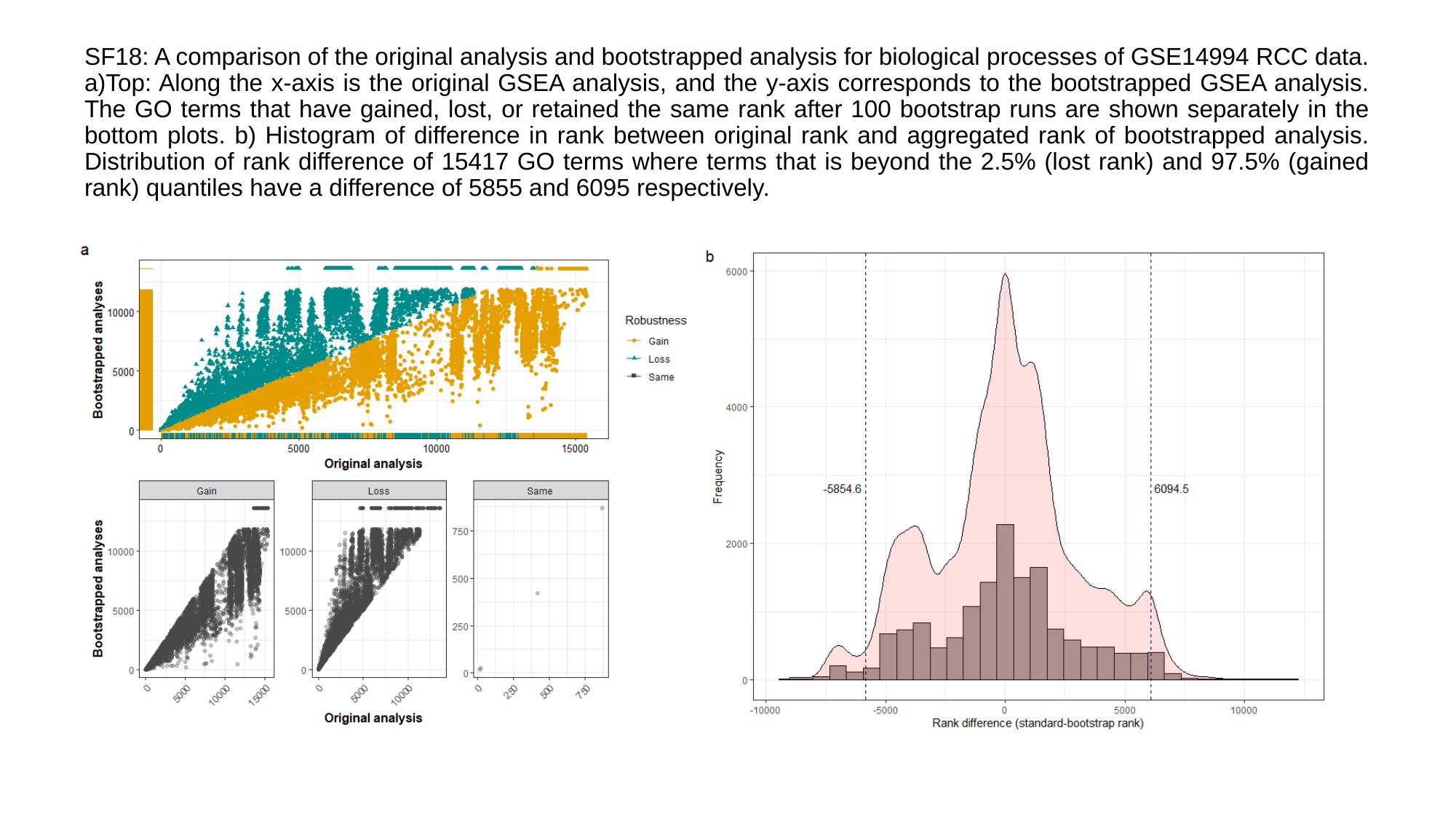

# SF18: A comparison of the original analysis and bootstrapped analysis for biological processes of GSE14994 RCC data. a)Top: Along the x-axis is the original GSEA analysis, and the y-axis corresponds to the bootstrapped GSEA analysis. The GO terms that have gained, lost, or retained the same rank after 100 bootstrap runs are shown separately in the bottom plots. b) Histogram of difference in rank between original rank and aggregated rank of bootstrapped analysis. Distribution of rank difference of 15417 GO terms where terms that is beyond the 2.5% (lost rank) and 97.5% (gained rank) quantiles have a difference of 5855 and 6095 respectively.

## Slide 21
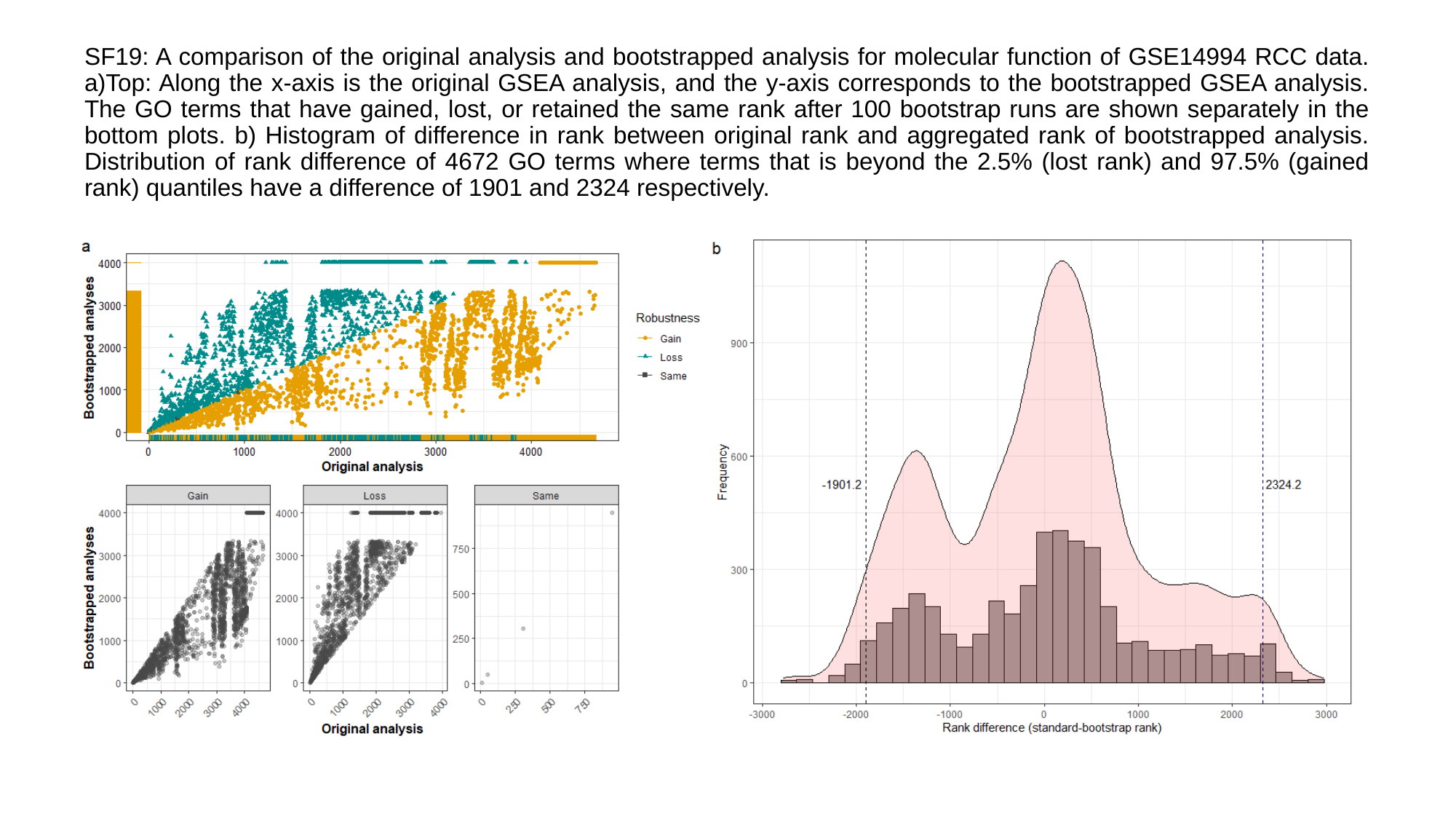

# SF19: A comparison of the original analysis and bootstrapped analysis for molecular function of GSE14994 RCC data. a)Top: Along the x-axis is the original GSEA analysis, and the y-axis corresponds to the bootstrapped GSEA analysis. The GO terms that have gained, lost, or retained the same rank after 100 bootstrap runs are shown separately in the bottom plots. b) Histogram of difference in rank between original rank and aggregated rank of bootstrapped analysis. Distribution of rank difference of 4672 GO terms where terms that is beyond the 2.5% (lost rank) and 97.5% (gained rank) quantiles have a difference of 1901 and 2324 respectively.

## Slide 22
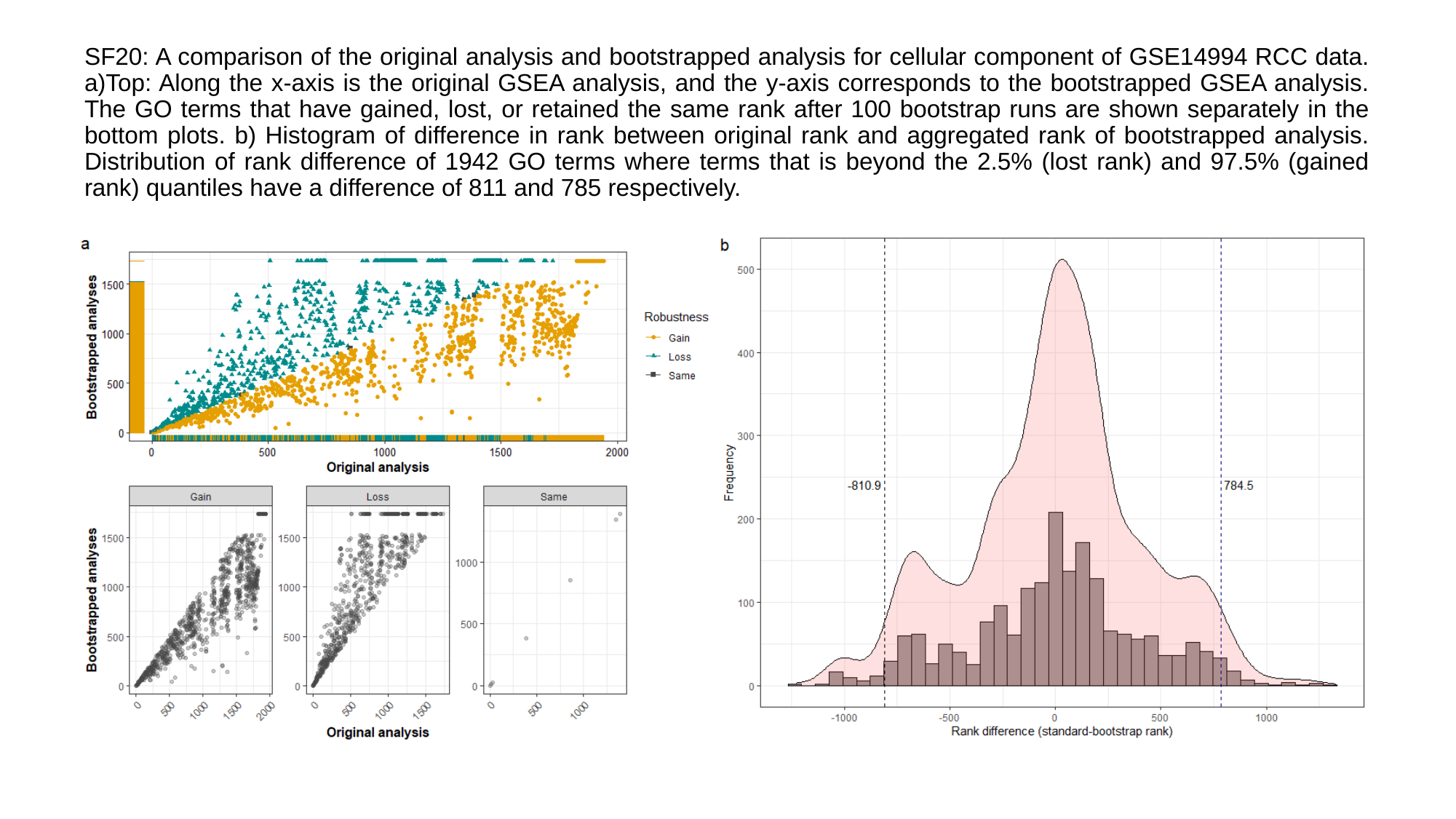

# SF20: A comparison of the original analysis and bootstrapped analysis for cellular component of GSE14994 RCC data. a)Top: Along the x-axis is the original GSEA analysis, and the y-axis corresponds to the bootstrapped GSEA analysis. The GO terms that have gained, lost, or retained the same rank after 100 bootstrap runs are shown separately in the bottom plots. b) Histogram of difference in rank between original rank and aggregated rank of bootstrapped analysis. Distribution of rank difference of 1942 GO terms where terms that is beyond the 2.5% (lost rank) and 97.5% (gained rank) quantiles have a difference of 811 and 785 respectively.

## Slide 23
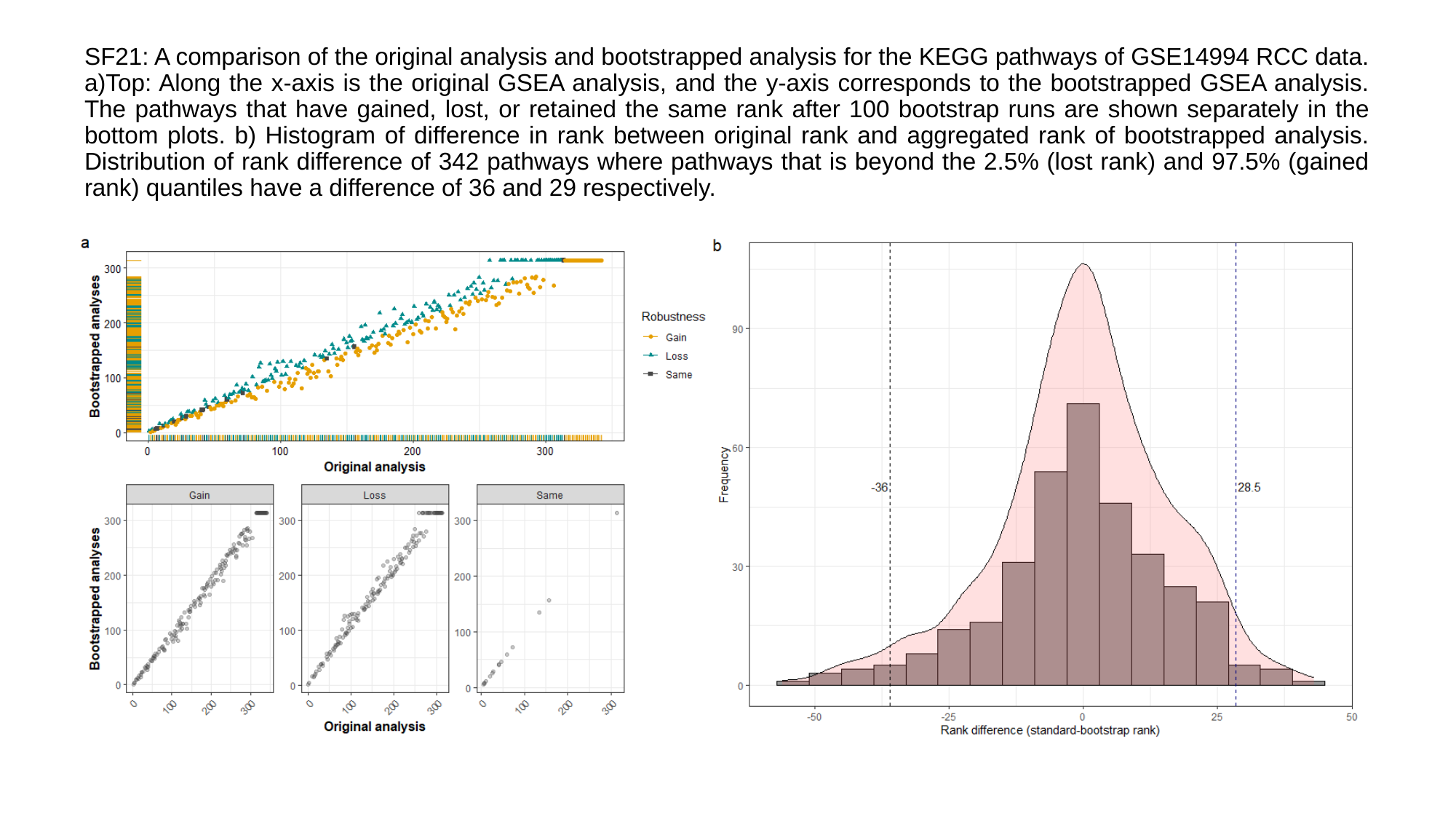

# SF21: A comparison of the original analysis and bootstrapped analysis for the KEGG pathways of GSE14994 RCC data. a)Top: Along the x-axis is the original GSEA analysis, and the y-axis corresponds to the bootstrapped GSEA analysis. The pathways that have gained, lost, or retained the same rank after 100 bootstrap runs are shown separately in the bottom plots. b) Histogram of difference in rank between original rank and aggregated rank of bootstrapped analysis. Distribution of rank difference of 342 pathways where pathways that is beyond the 2.5% (lost rank) and 97.5% (gained rank) quantiles have a difference of 36 and 29 respectively.

## Slide 24
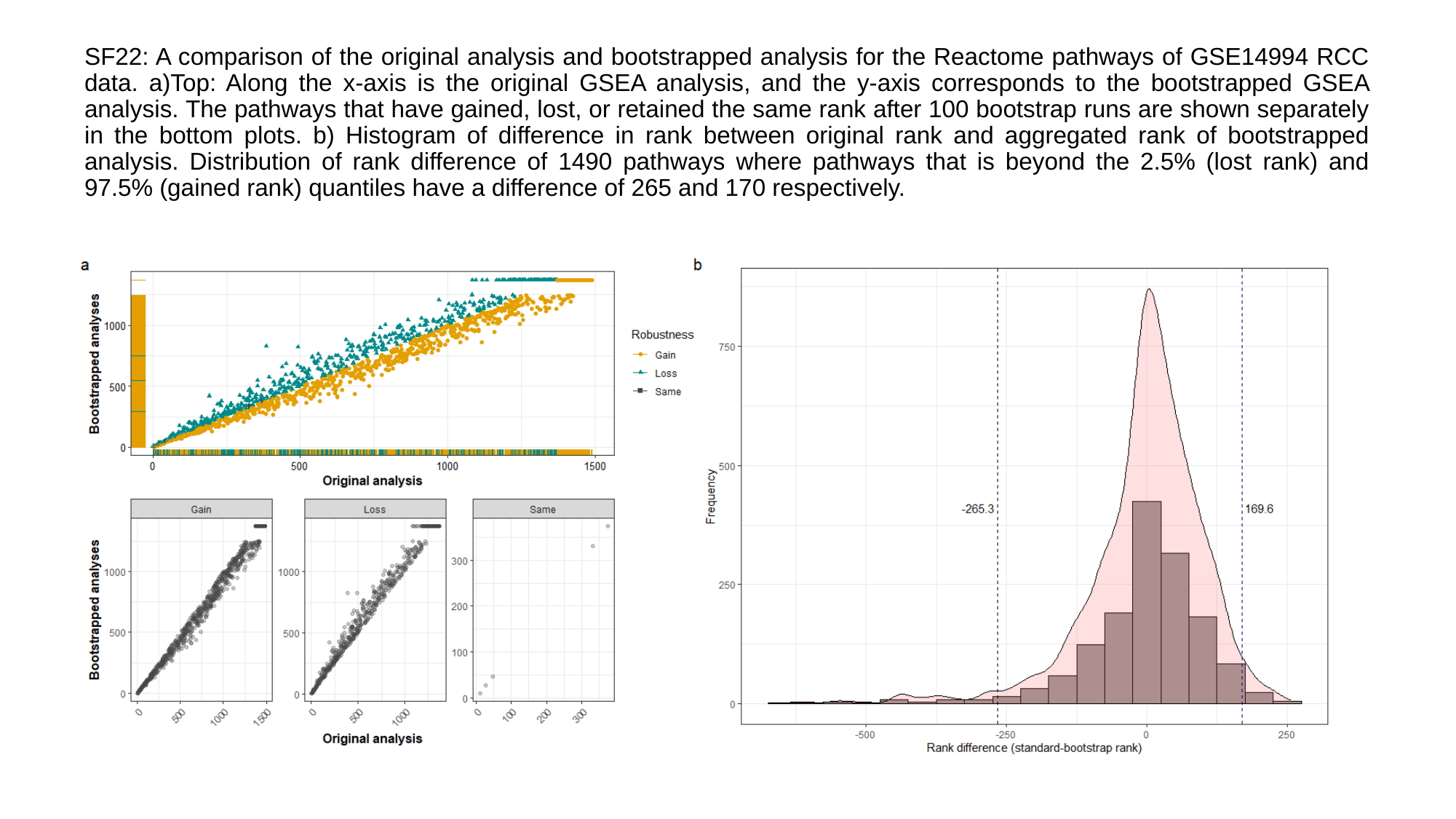

# SF22: A comparison of the original analysis and bootstrapped analysis for the Reactome pathways of GSE14994 RCC data. a)Top: Along the x-axis is the original GSEA analysis, and the y-axis corresponds to the bootstrapped GSEA analysis. The pathways that have gained, lost, or retained the same rank after 100 bootstrap runs are shown separately in the bottom plots. b) Histogram of difference in rank between original rank and aggregated rank of bootstrapped analysis. Distribution of rank difference of 1490 pathways where pathways that is beyond the 2.5% (lost rank) and 97.5% (gained rank) quantiles have a difference of 265 and 170 respectively.

## Slide 25
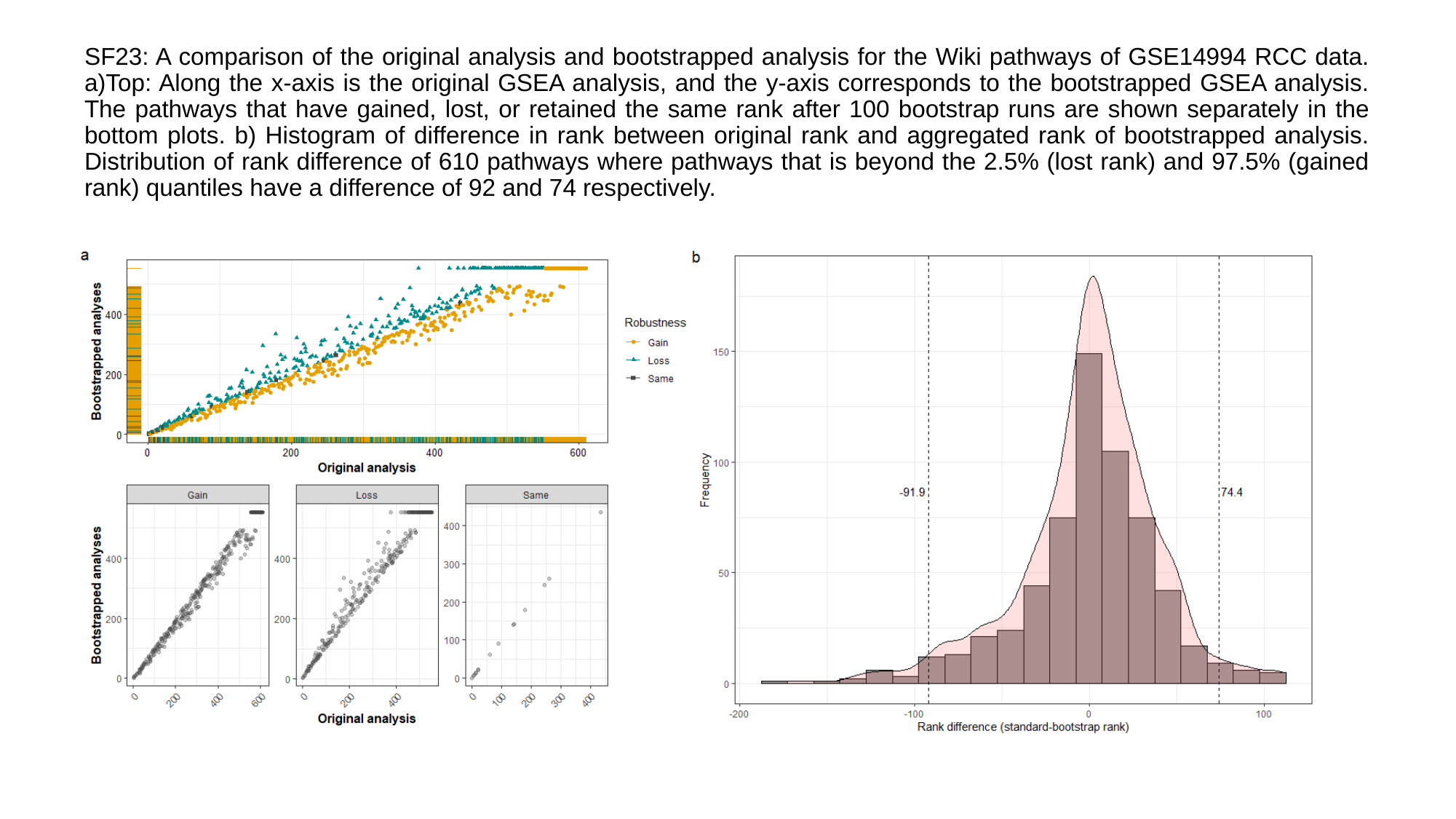

# SF23: A comparison of the original analysis and bootstrapped analysis for the Wiki pathways of GSE14994 RCC data. a)Top: Along the x-axis is the original GSEA analysis, and the y-axis corresponds to the bootstrapped GSEA analysis. The pathways that have gained, lost, or retained the same rank after 100 bootstrap runs are shown separately in the bottom plots. b) Histogram of difference in rank between original rank and aggregated rank of bootstrapped analysis. Distribution of rank difference of 610 pathways where pathways that is beyond the 2.5% (lost rank) and 97.5% (gained rank) quantiles have a difference of 92 and 74 respectively.

## Slide 26
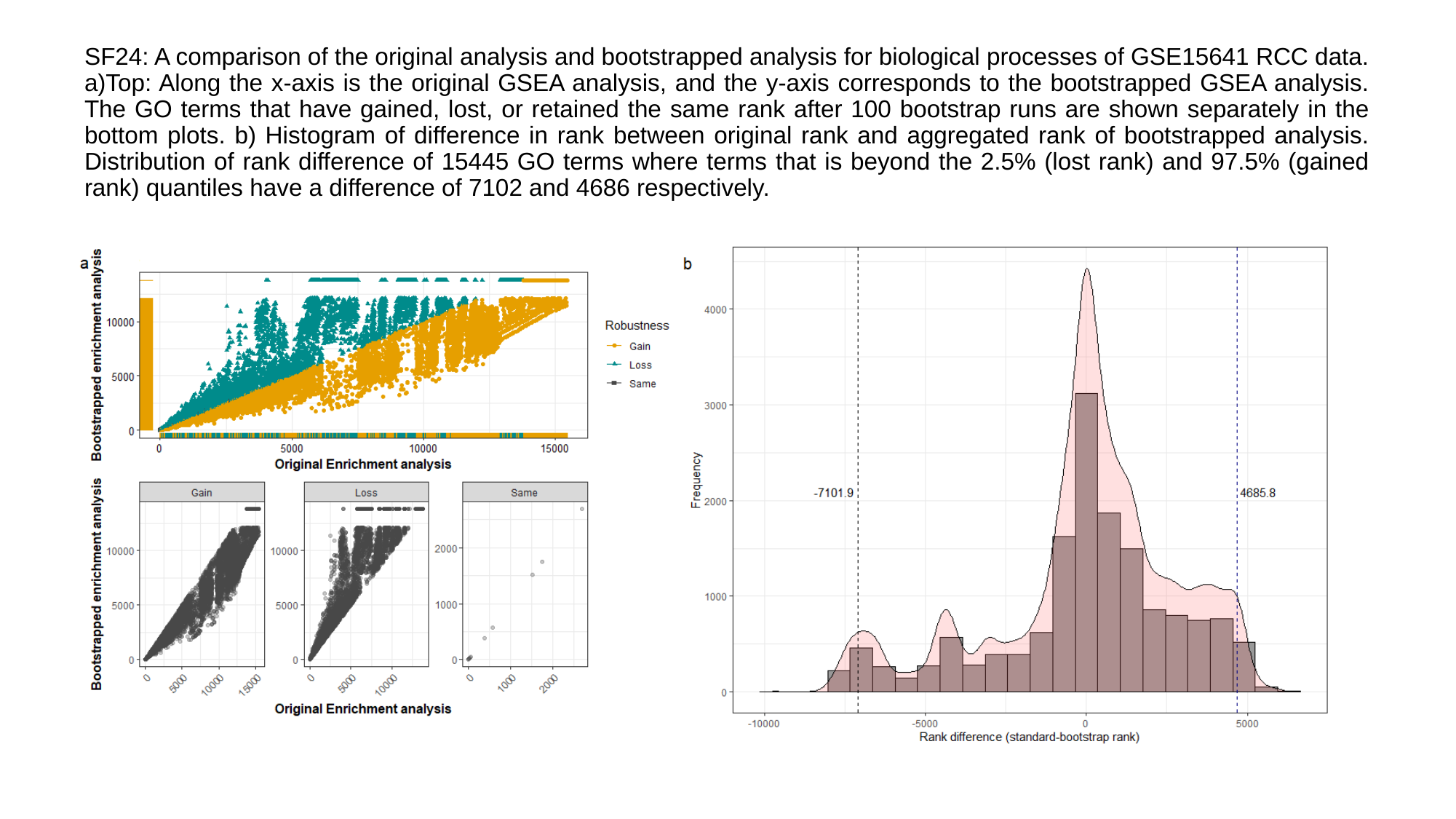

# SF24: A comparison of the original analysis and bootstrapped analysis for biological processes of GSE15641 RCC data. a)Top: Along the x-axis is the original GSEA analysis, and the y-axis corresponds to the bootstrapped GSEA analysis. The GO terms that have gained, lost, or retained the same rank after 100 bootstrap runs are shown separately in the bottom plots. b) Histogram of difference in rank between original rank and aggregated rank of bootstrapped analysis. Distribution of rank difference of 15445 GO terms where terms that is beyond the 2.5% (lost rank) and 97.5% (gained rank) quantiles have a difference of 7102 and 4686 respectively.

## Slide 27
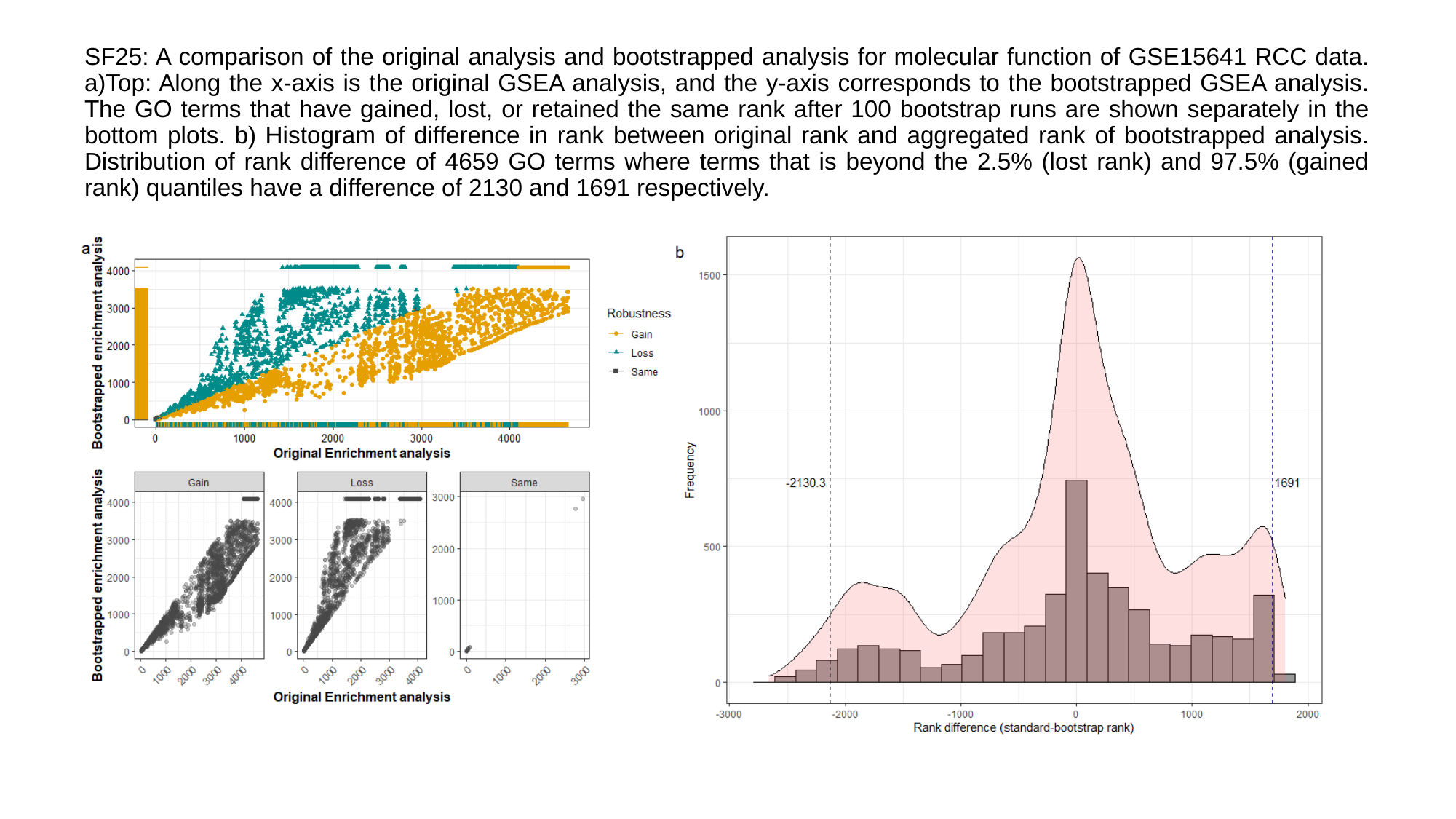

# SF25: A comparison of the original analysis and bootstrapped analysis for molecular function of GSE15641 RCC data. a)Top: Along the x-axis is the original GSEA analysis, and the y-axis corresponds to the bootstrapped GSEA analysis. The GO terms that have gained, lost, or retained the same rank after 100 bootstrap runs are shown separately in the bottom plots. b) Histogram of difference in rank between original rank and aggregated rank of bootstrapped analysis. Distribution of rank difference of 4659 GO terms where terms that is beyond the 2.5% (lost rank) and 97.5% (gained rank) quantiles have a difference of 2130 and 1691 respectively.

## Slide 28
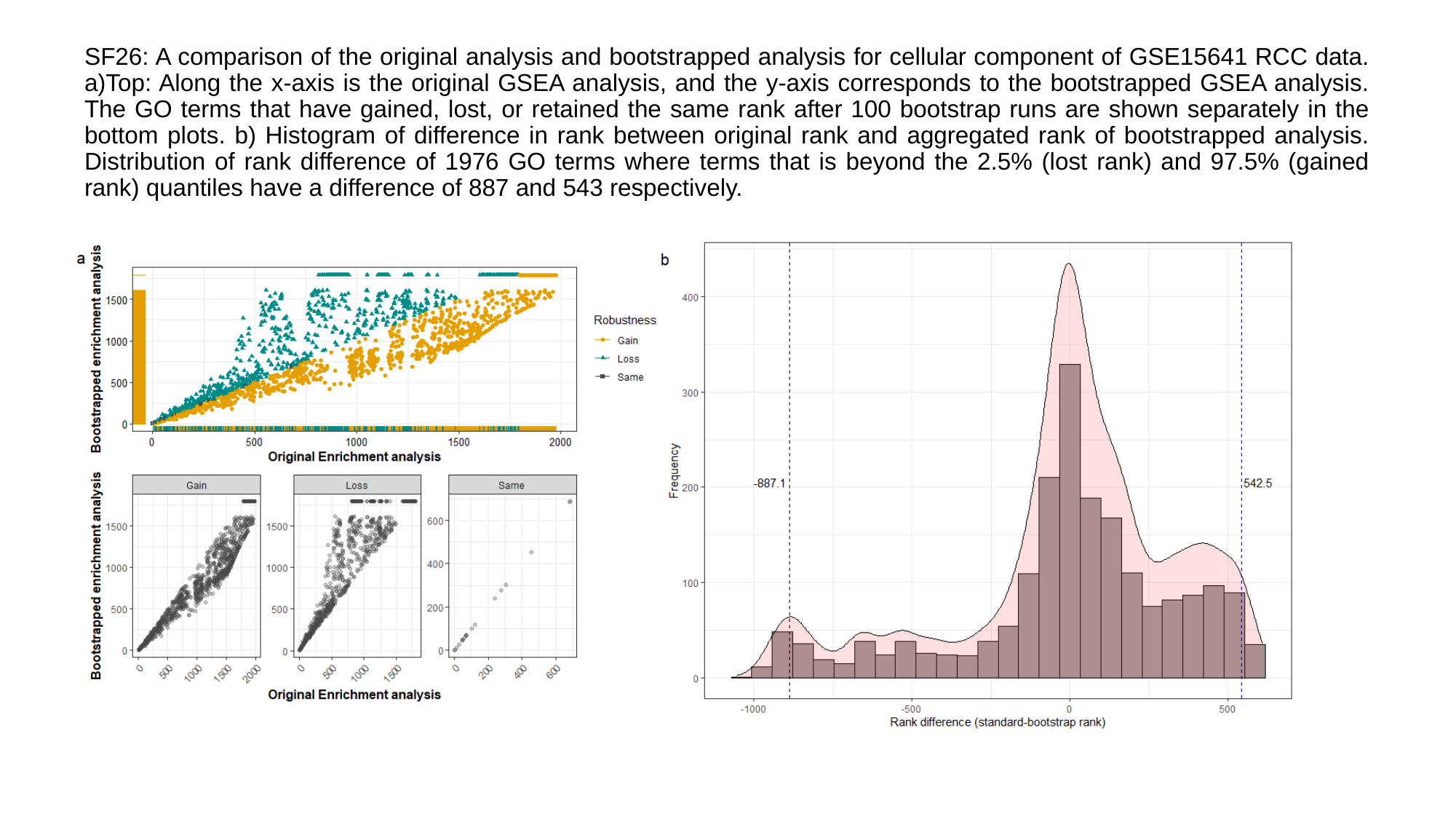

# SF26: A comparison of the original analysis and bootstrapped analysis for cellular component of GSE15641 RCC data. a)Top: Along the x-axis is the original GSEA analysis, and the y-axis corresponds to the bootstrapped GSEA analysis. The GO terms that have gained, lost, or retained the same rank after 100 bootstrap runs are shown separately in the bottom plots. b) Histogram of difference in rank between original rank and aggregated rank of bootstrapped analysis. Distribution of rank difference of 1976 GO terms where terms that is beyond the 2.5% (lost rank) and 97.5% (gained rank) quantiles have a difference of 887 and 543 respectively.

## Slide 29
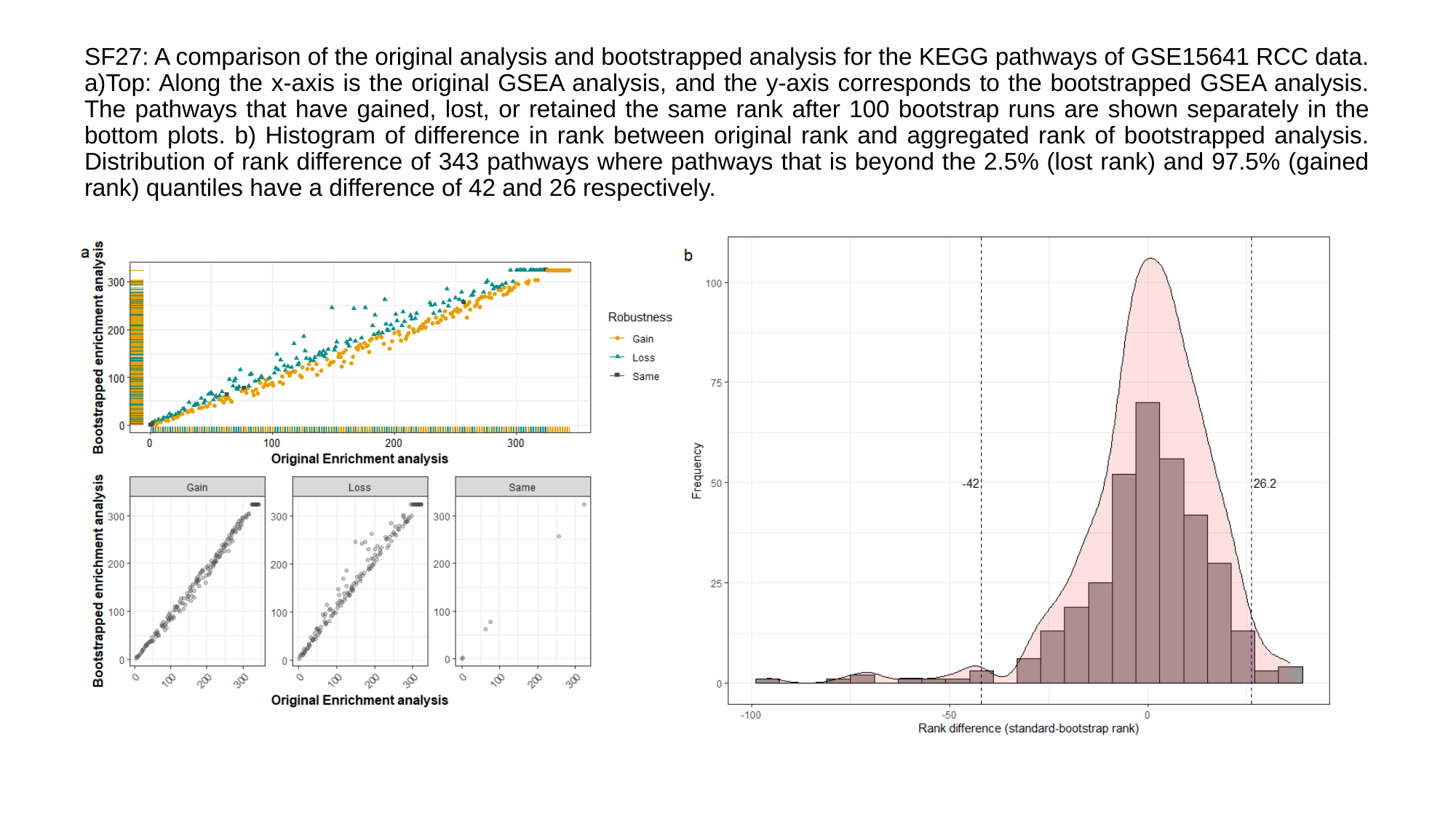

# SF27: A comparison of the original analysis and bootstrapped analysis for the KEGG pathways of GSE15641 RCC data. a)Top: Along the x-axis is the original GSEA analysis, and the y-axis corresponds to the bootstrapped GSEA analysis. The pathways that have gained, lost, or retained the same rank after 100 bootstrap runs are shown separately in the bottom plots. b) Histogram of difference in rank between original rank and aggregated rank of bootstrapped analysis. Distribution of rank difference of 343 pathways where pathways that is beyond the 2.5% (lost rank) and 97.5% (gained rank) quantiles have a difference of 42 and 26 respectively.

## Slide 30
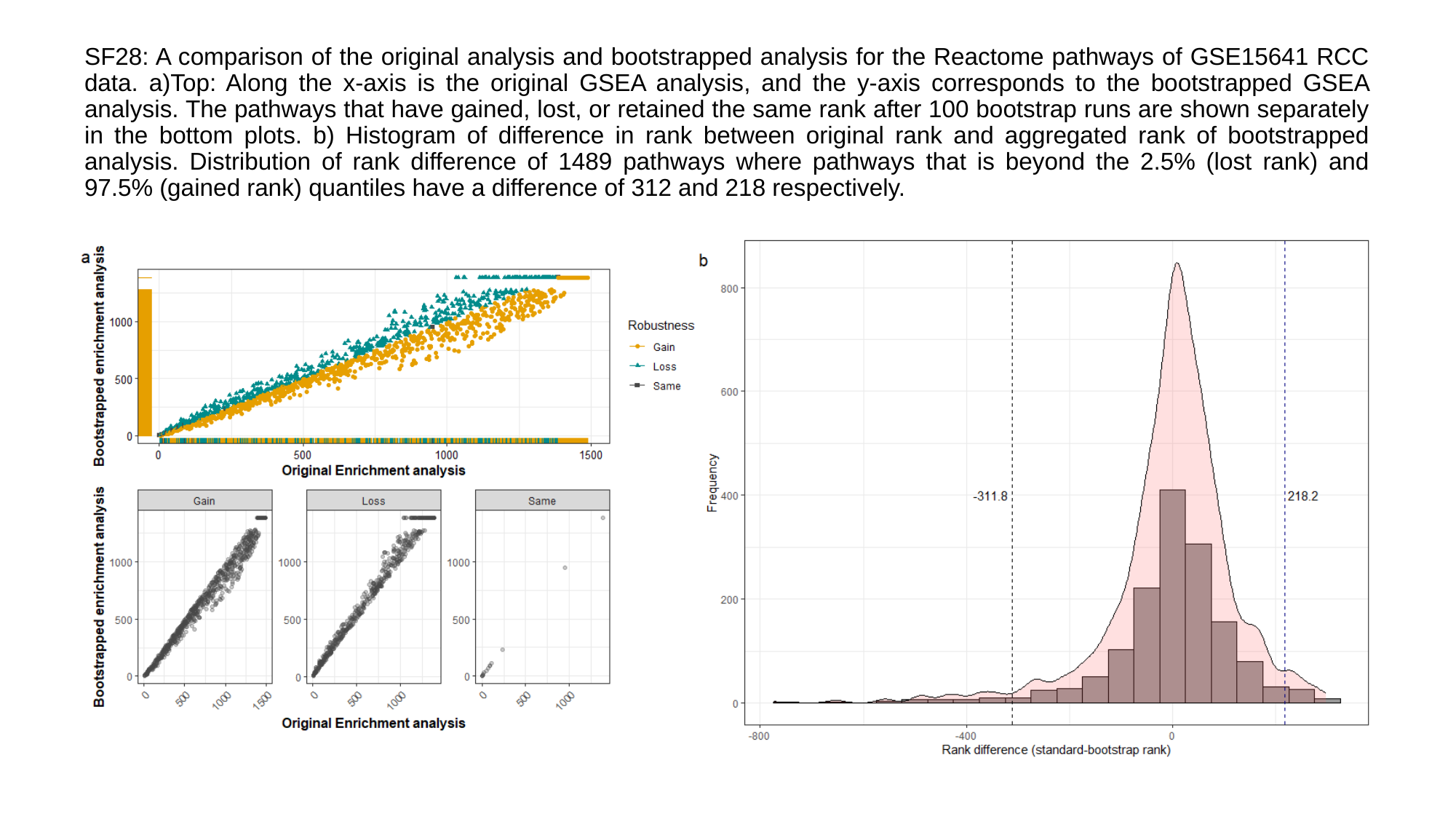

# SF28: A comparison of the original analysis and bootstrapped analysis for the Reactome pathways of GSE15641 RCC data. a)Top: Along the x-axis is the original GSEA analysis, and the y-axis corresponds to the bootstrapped GSEA analysis. The pathways that have gained, lost, or retained the same rank after 100 bootstrap runs are shown separately in the bottom plots. b) Histogram of difference in rank between original rank and aggregated rank of bootstrapped analysis. Distribution of rank difference of 1489 pathways where pathways that is beyond the 2.5% (lost rank) and 97.5% (gained rank) quantiles have a difference of 312 and 218 respectively.

## Slide 31
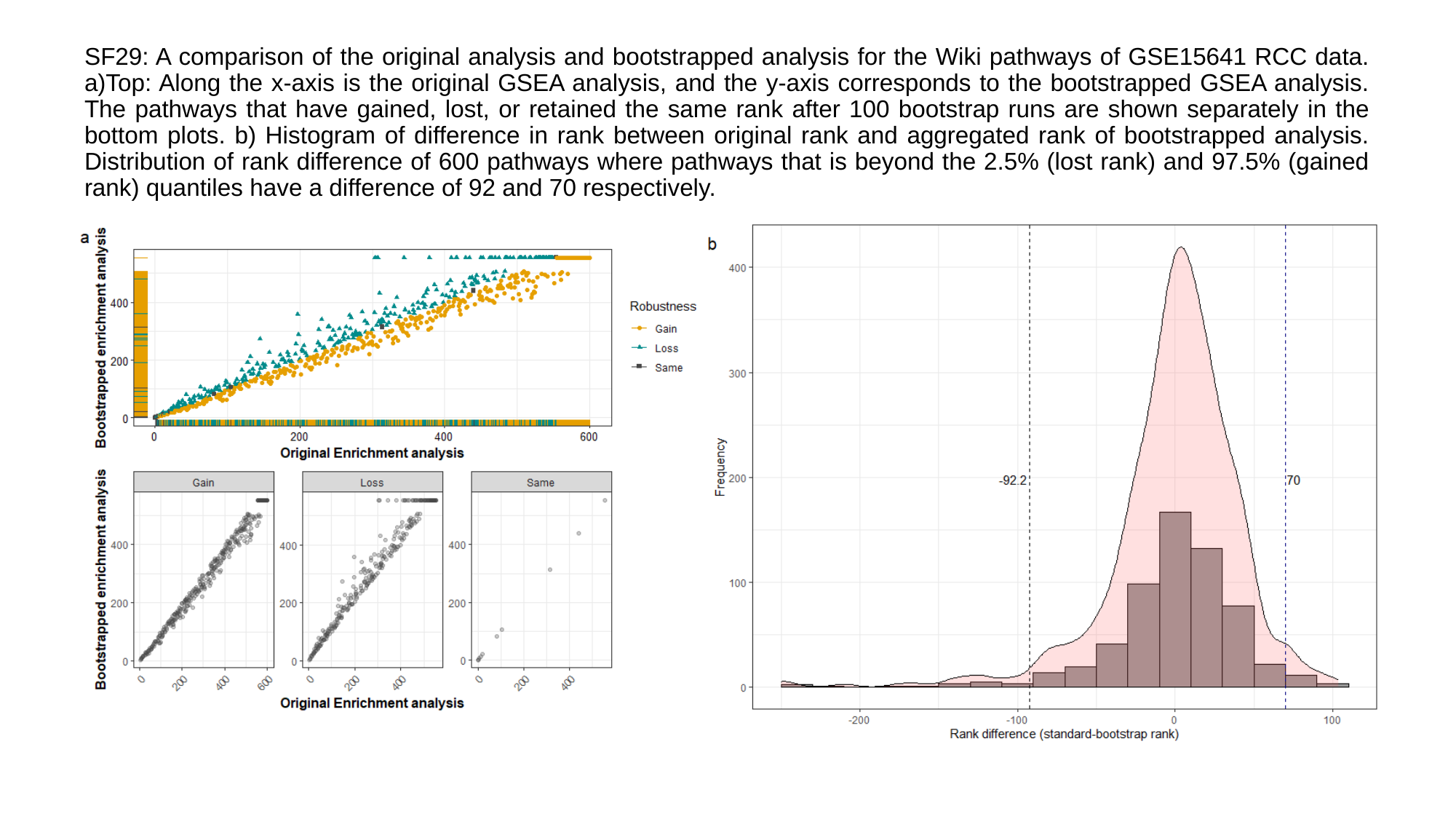

# SF29: A comparison of the original analysis and bootstrapped analysis for the Wiki pathways of GSE15641 RCC data. a)Top: Along the x-axis is the original GSEA analysis, and the y-axis corresponds to the bootstrapped GSEA analysis. The pathways that have gained, lost, or retained the same rank after 100 bootstrap runs are shown separately in the bottom plots. b) Histogram of difference in rank between original rank and aggregated rank of bootstrapped analysis. Distribution of rank difference of 600 pathways where pathways that is beyond the 2.5% (lost rank) and 97.5% (gained rank) quantiles have a difference of 92 and 70 respectively.

## Slide 32
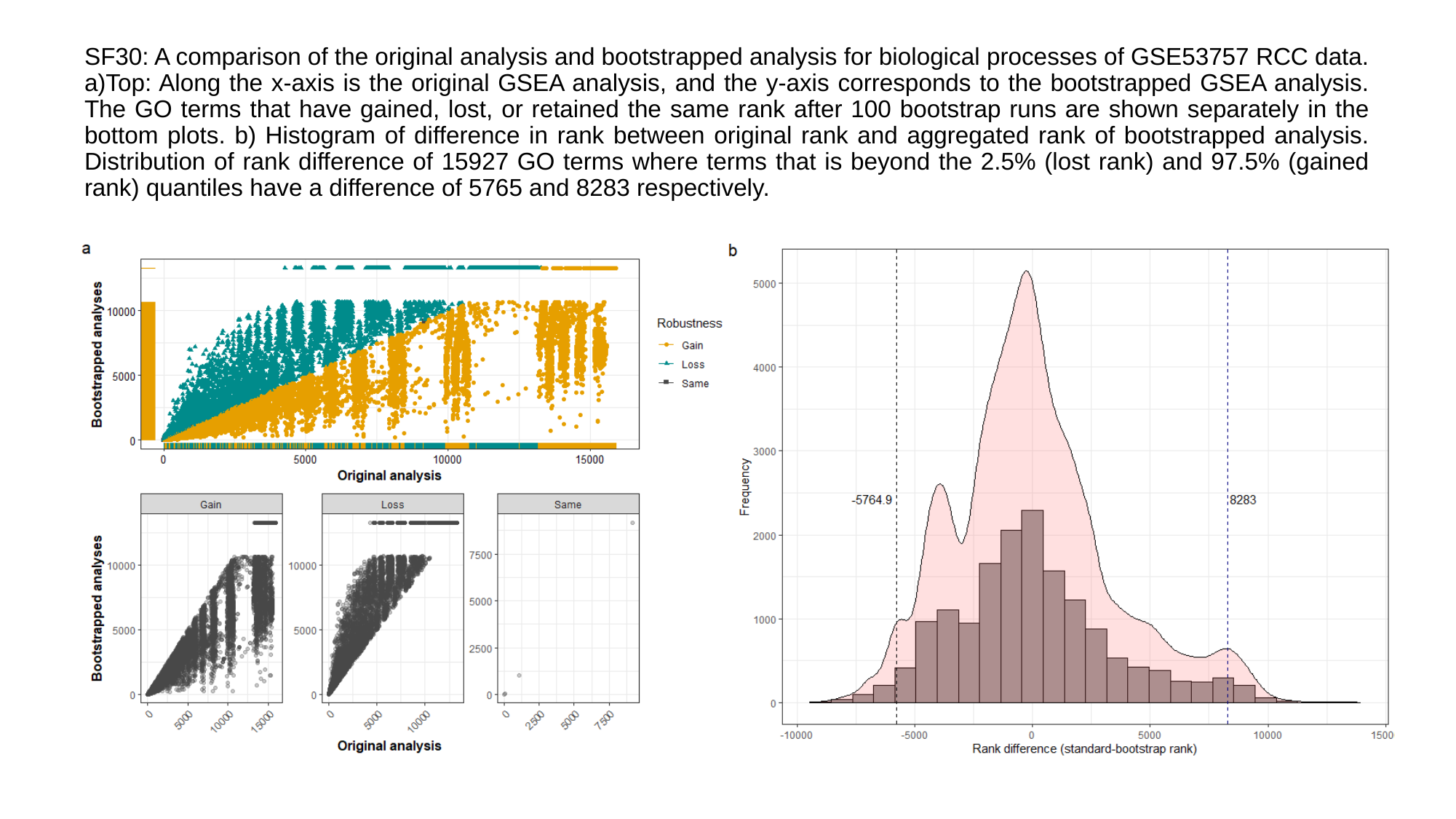

# SF30: A comparison of the original analysis and bootstrapped analysis for biological processes of GSE53757 RCC data. a)Top: Along the x-axis is the original GSEA analysis, and the y-axis corresponds to the bootstrapped GSEA analysis. The GO terms that have gained, lost, or retained the same rank after 100 bootstrap runs are shown separately in the bottom plots. b) Histogram of difference in rank between original rank and aggregated rank of bootstrapped analysis. Distribution of rank difference of 15927 GO terms where terms that is beyond the 2.5% (lost rank) and 97.5% (gained rank) quantiles have a difference of 5765 and 8283 respectively.

## Slide 33
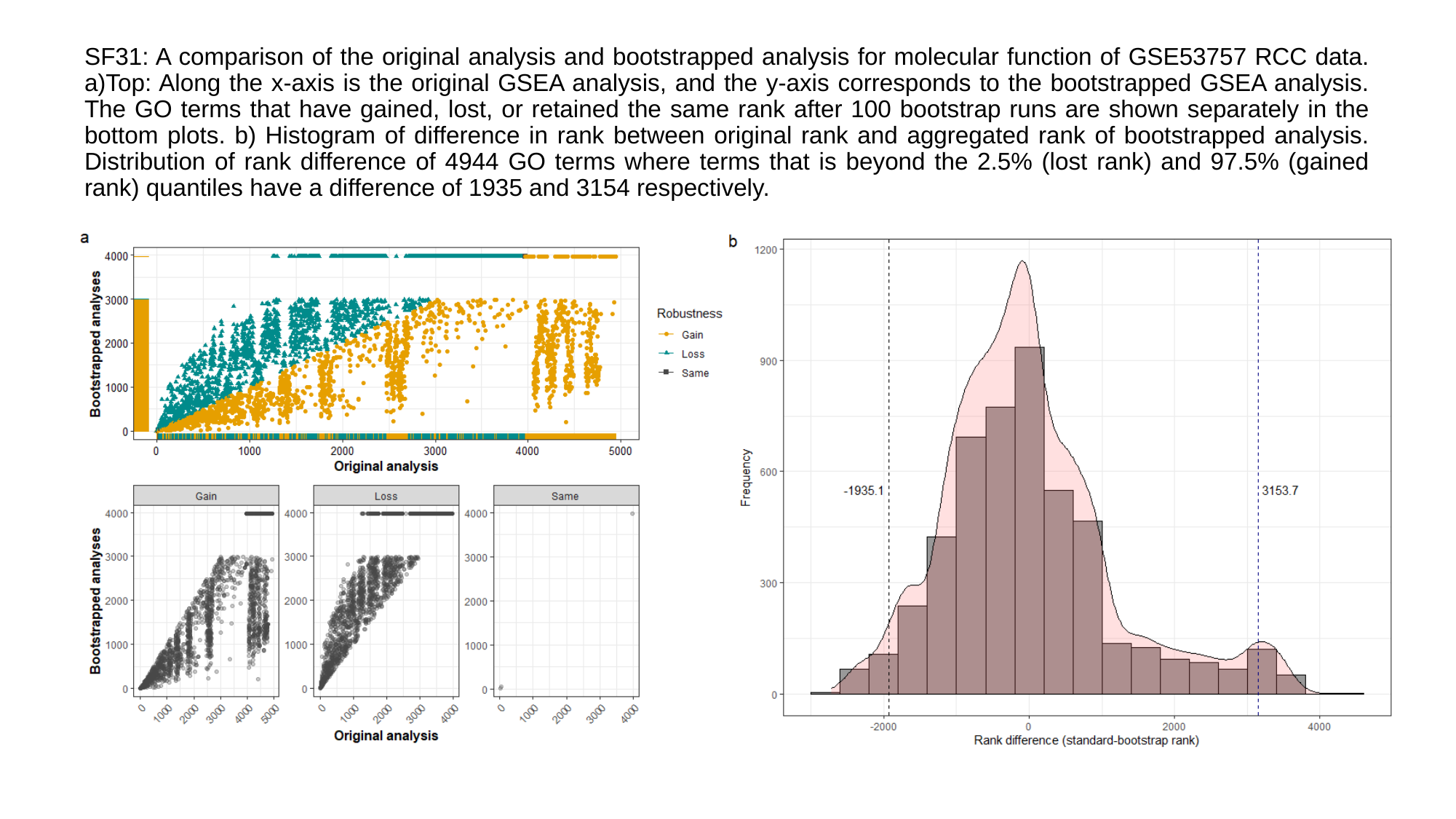

# SF31: A comparison of the original analysis and bootstrapped analysis for molecular function of GSE53757 RCC data. a)Top: Along the x-axis is the original GSEA analysis, and the y-axis corresponds to the bootstrapped GSEA analysis. The GO terms that have gained, lost, or retained the same rank after 100 bootstrap runs are shown separately in the bottom plots. b) Histogram of difference in rank between original rank and aggregated rank of bootstrapped analysis. Distribution of rank difference of 4944 GO terms where terms that is beyond the 2.5% (lost rank) and 97.5% (gained rank) quantiles have a difference of 1935 and 3154 respectively.

## Slide 34
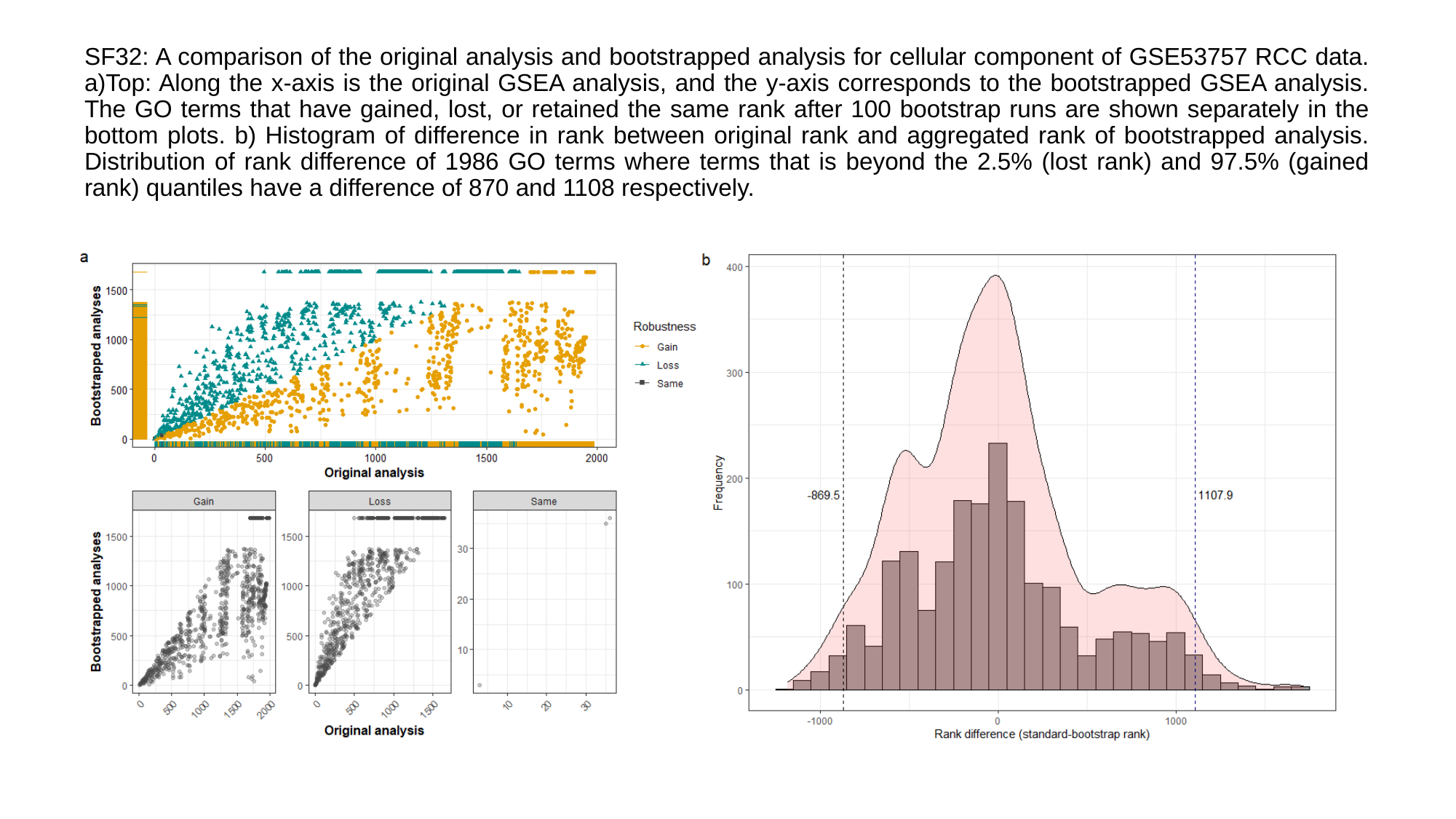

# SF32: A comparison of the original analysis and bootstrapped analysis for cellular component of GSE53757 RCC data. a)Top: Along the x-axis is the original GSEA analysis, and the y-axis corresponds to the bootstrapped GSEA analysis. The GO terms that have gained, lost, or retained the same rank after 100 bootstrap runs are shown separately in the bottom plots. b) Histogram of difference in rank between original rank and aggregated rank of bootstrapped analysis. Distribution of rank difference of 1986 GO terms where terms that is beyond the 2.5% (lost rank) and 97.5% (gained rank) quantiles have a difference of 870 and 1108 respectively.

## Slide 35
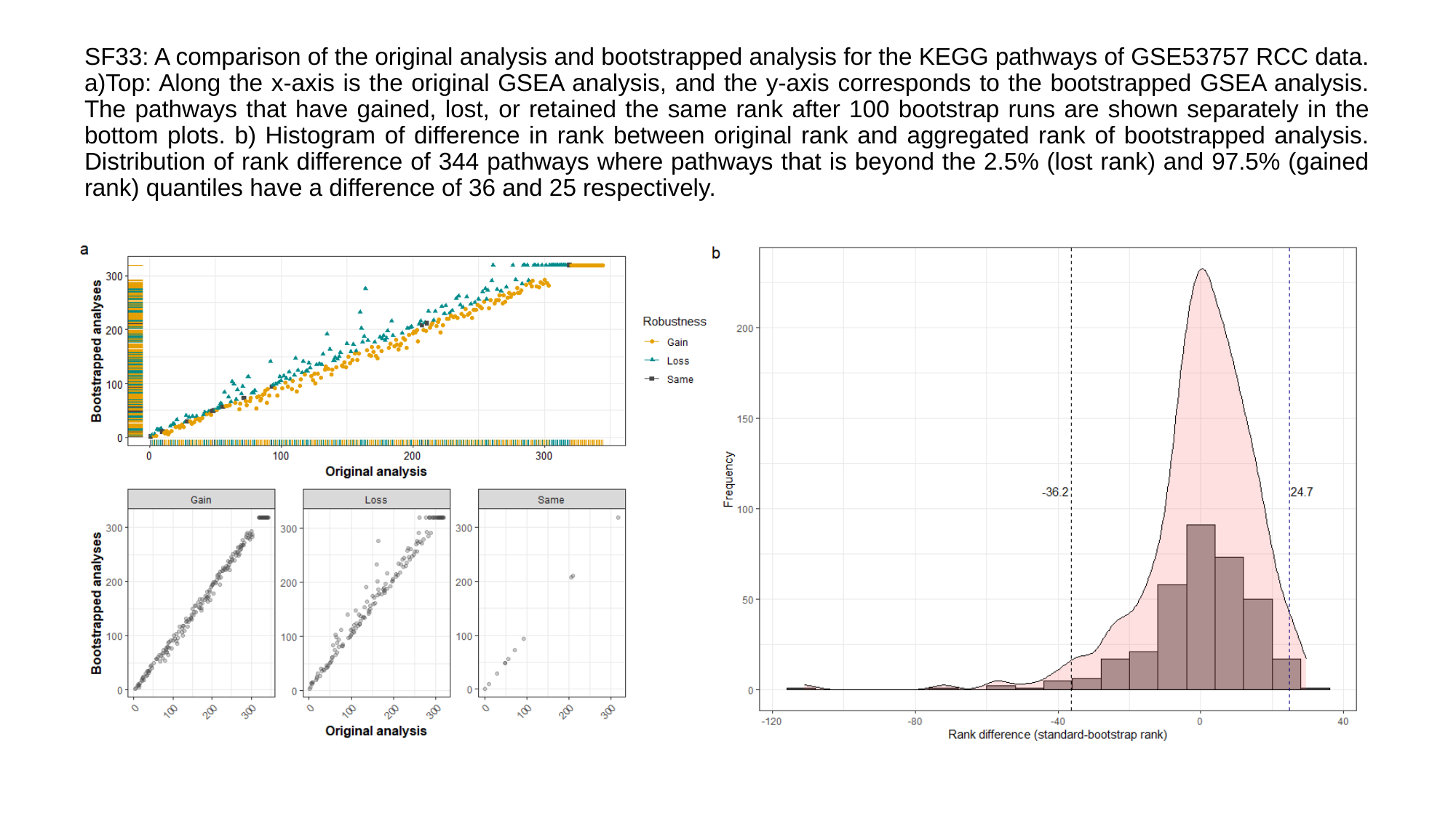

# SF33: A comparison of the original analysis and bootstrapped analysis for the KEGG pathways of GSE53757 RCC data. a)Top: Along the x-axis is the original GSEA analysis, and the y-axis corresponds to the bootstrapped GSEA analysis. The pathways that have gained, lost, or retained the same rank after 100 bootstrap runs are shown separately in the bottom plots. b) Histogram of difference in rank between original rank and aggregated rank of bootstrapped analysis. Distribution of rank difference of 344 pathways where pathways that is beyond the 2.5% (lost rank) and 97.5% (gained rank) quantiles have a difference of 36 and 25 respectively.

## Slide 36
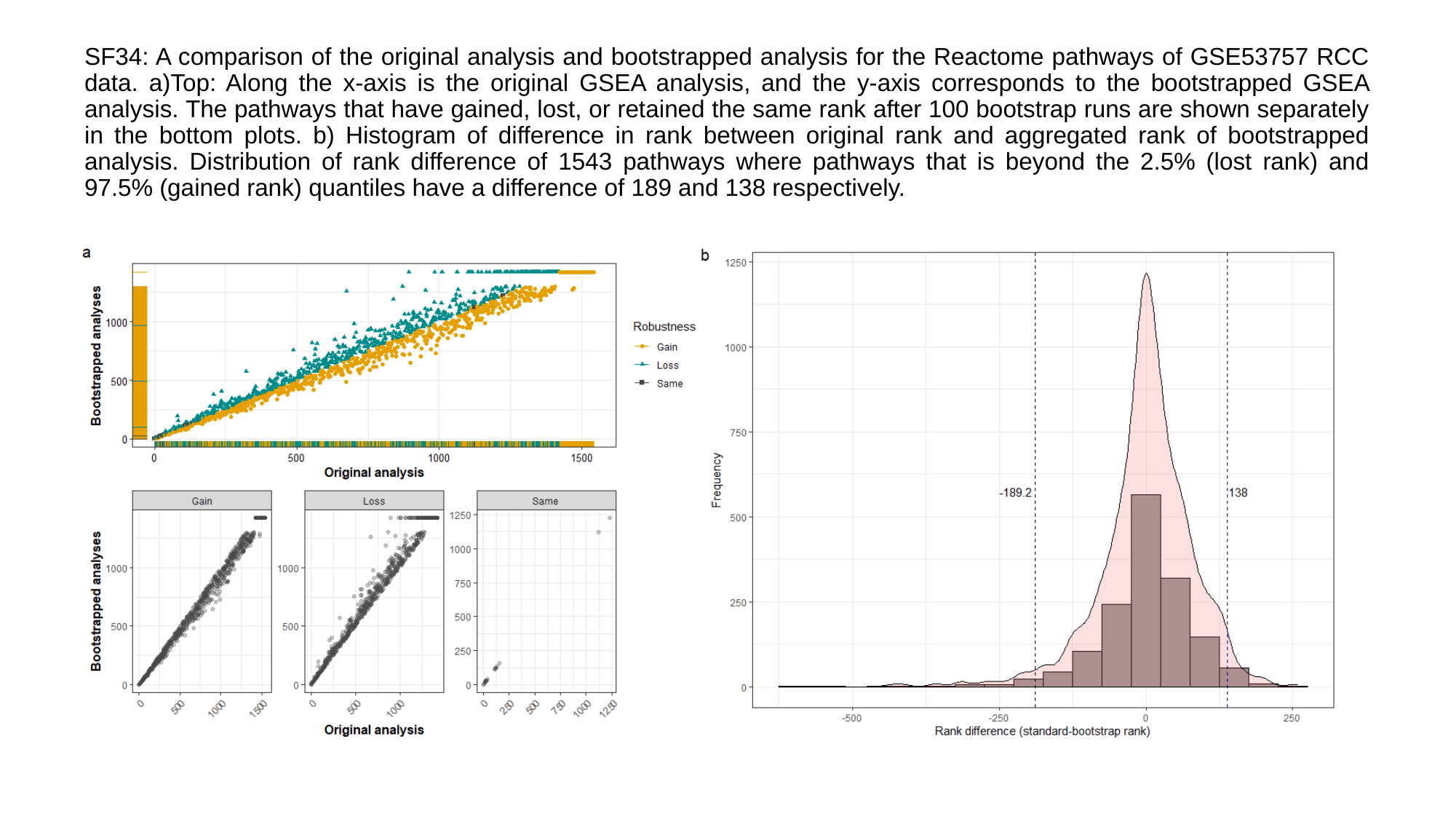

# SF34: A comparison of the original analysis and bootstrapped analysis for the Reactome pathways of GSE53757 RCC data. a)Top: Along the x-axis is the original GSEA analysis, and the y-axis corresponds to the bootstrapped GSEA analysis. The pathways that have gained, lost, or retained the same rank after 100 bootstrap runs are shown separately in the bottom plots. b) Histogram of difference in rank between original rank and aggregated rank of bootstrapped analysis. Distribution of rank difference of 1543 pathways where pathways that is beyond the 2.5% (lost rank) and 97.5% (gained rank) quantiles have a difference of 189 and 138 respectively.

## Slide 37
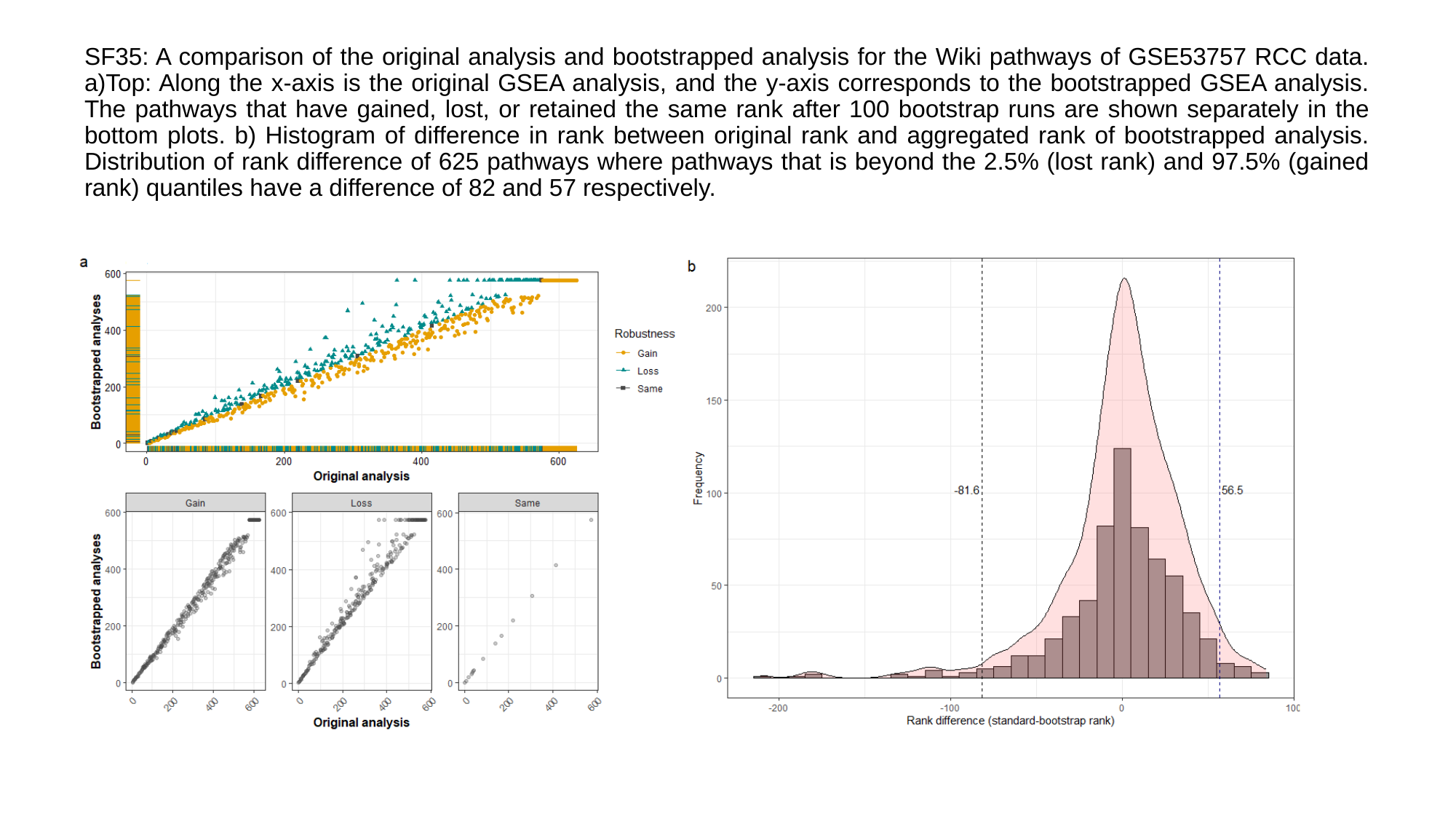

# SF35: A comparison of the original analysis and bootstrapped analysis for the Wiki pathways of GSE53757 RCC data. a)Top: Along the x-axis is the original GSEA analysis, and the y-axis corresponds to the bootstrapped GSEA analysis. The pathways that have gained, lost, or retained the same rank after 100 bootstrap runs are shown separately in the bottom plots. b) Histogram of difference in rank between original rank and aggregated rank of bootstrapped analysis. Distribution of rank difference of 625 pathways where pathways that is beyond the 2.5% (lost rank) and 97.5% (gained rank) quantiles have a difference of 82 and 57 respectively.

## Slide 38
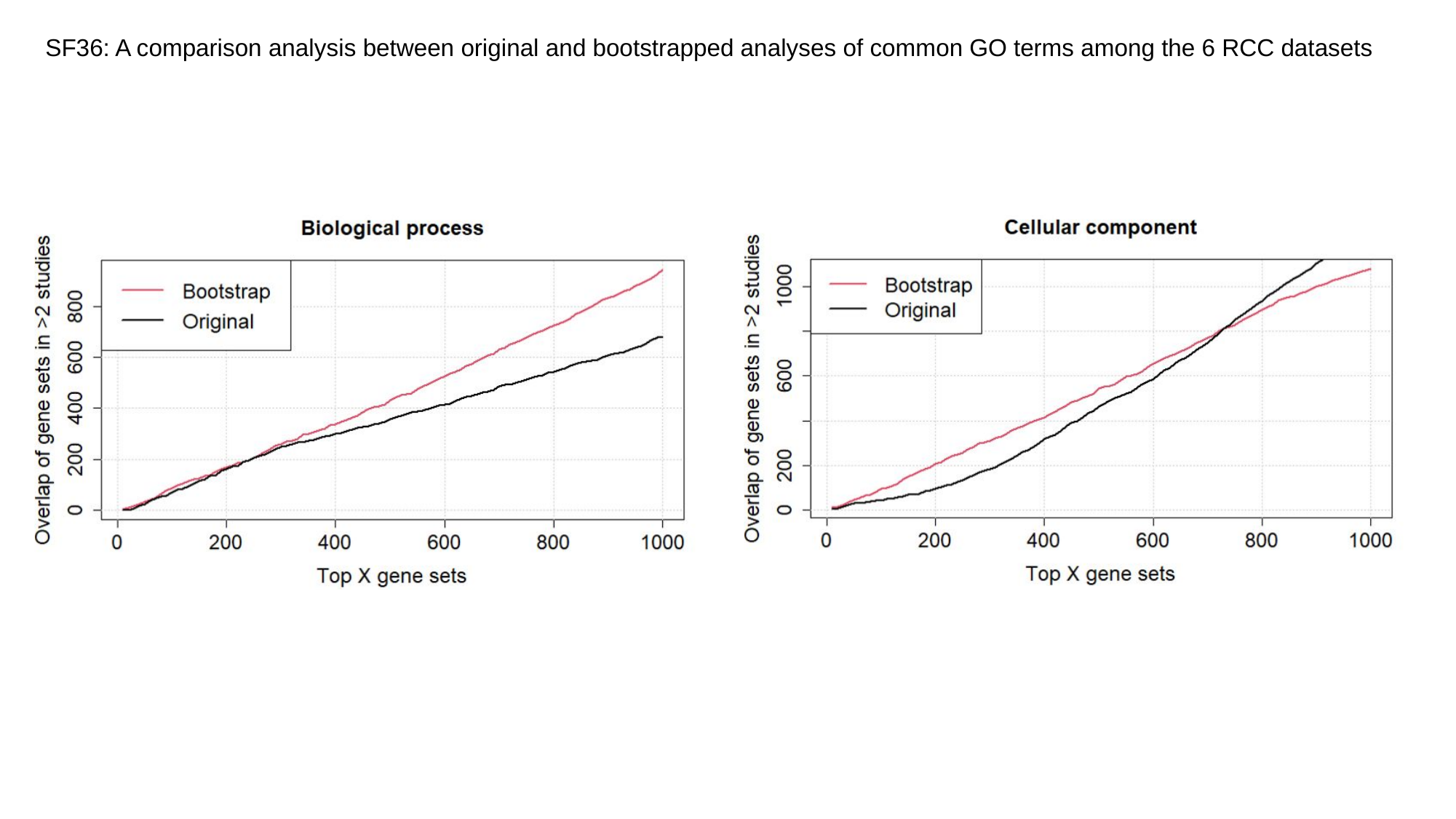

# SF36: A comparison analysis between original and bootstrapped analyses of common GO terms among the 6 RCC datasets

## Slide 39
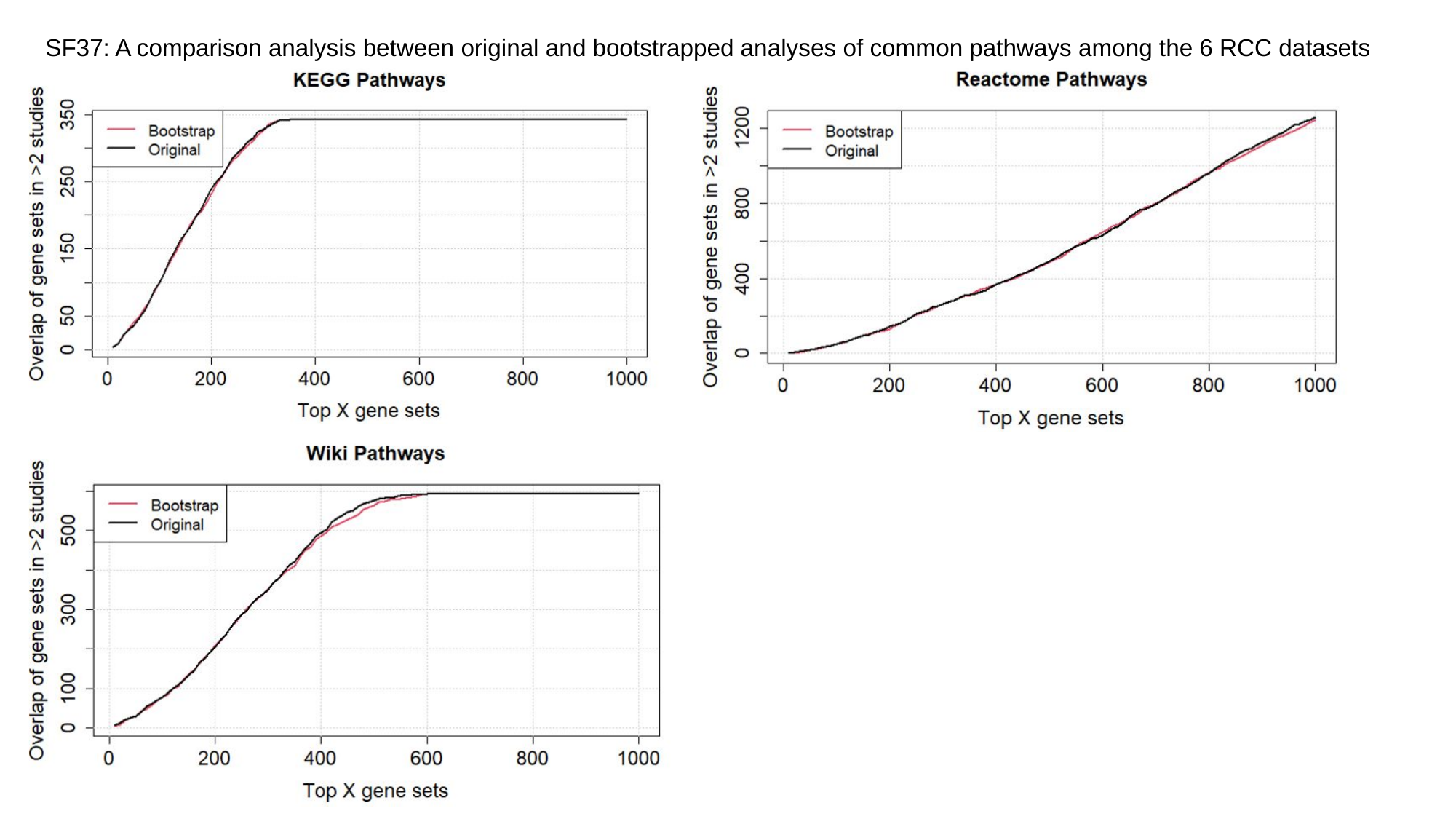

# SF37: A comparison analysis between original and bootstrapped analyses of common pathways among the 6 RCC datasets

## Slide 40
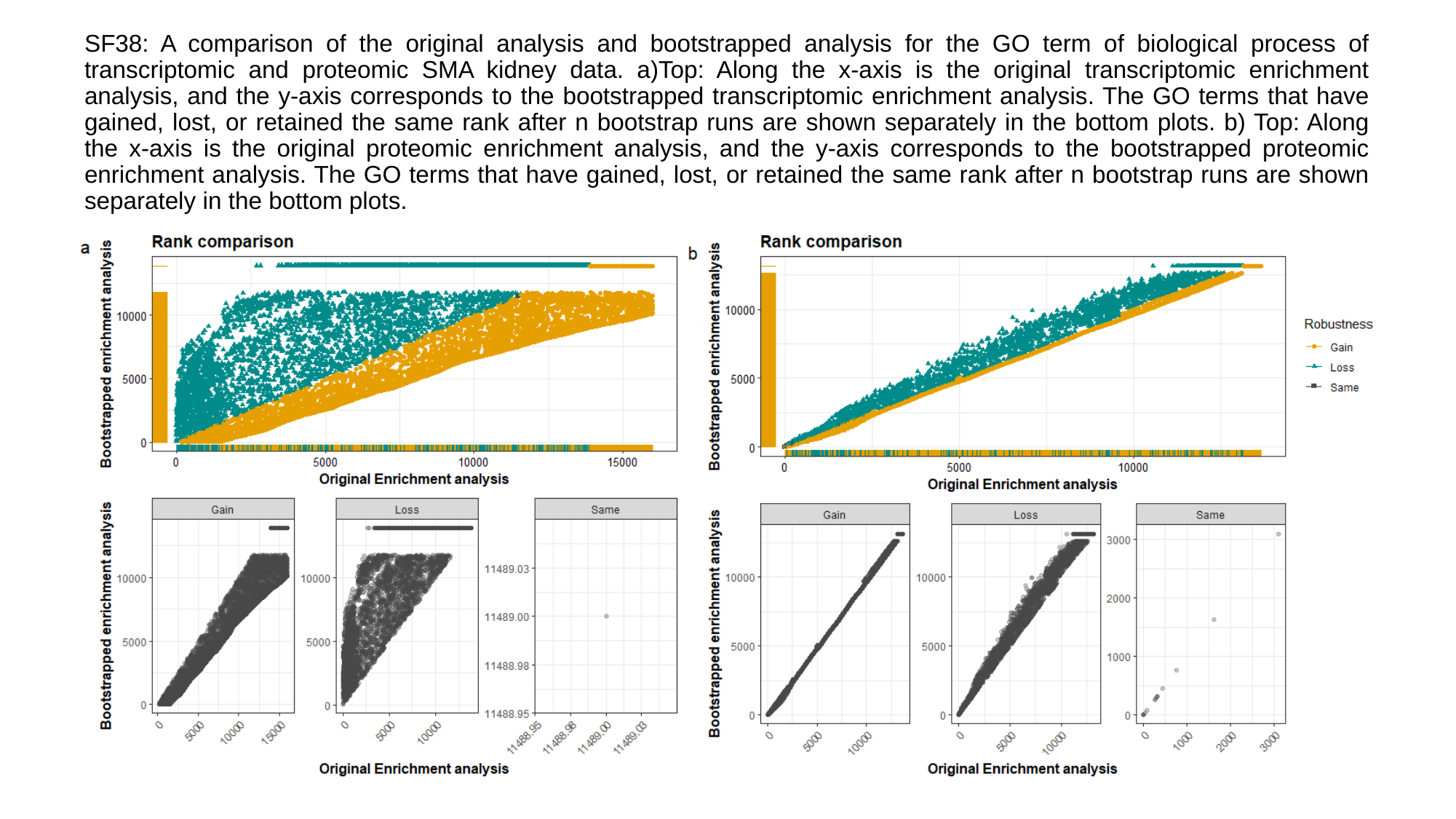

# SF38: A comparison of the original analysis and bootstrapped analysis for the GO term of biological process of transcriptomic and proteomic SMA kidney data. a)Top: Along the x-axis is the original transcriptomic enrichment analysis, and the y-axis corresponds to the bootstrapped transcriptomic enrichment analysis. The GO terms that have gained, lost, or retained the same rank after n bootstrap runs are shown separately in the bottom plots. b) Top: Along the x-axis is the original proteomic enrichment analysis, and the y-axis corresponds to the bootstrapped proteomic enrichment analysis. The GO terms that have gained, lost, or retained the same rank after n bootstrap runs are shown separately in the bottom plots.

## Slide 41
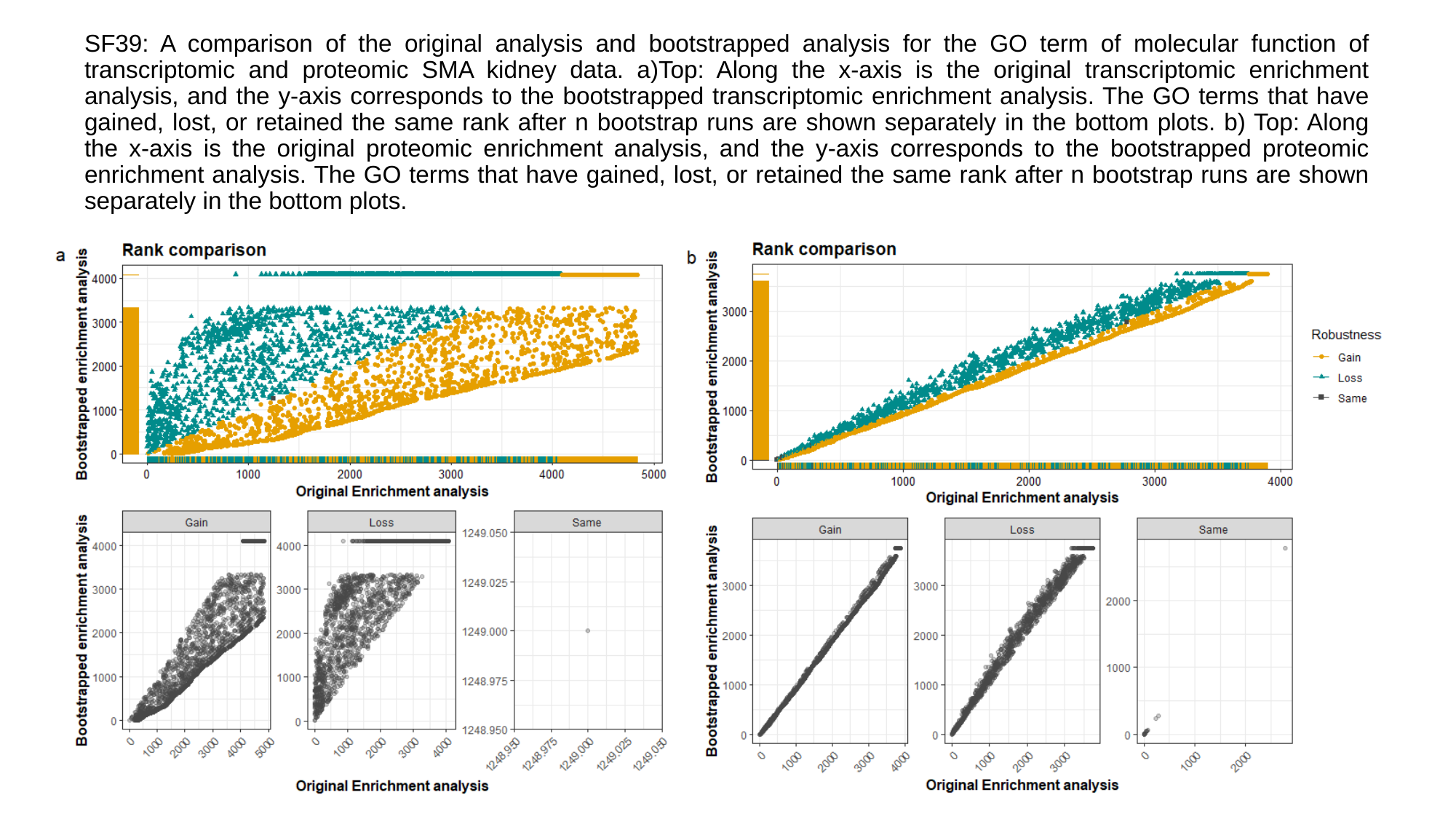

# SF39: A comparison of the original analysis and bootstrapped analysis for the GO term of molecular function of transcriptomic and proteomic SMA kidney data. a)Top: Along the x-axis is the original transcriptomic enrichment analysis, and the y-axis corresponds to the bootstrapped transcriptomic enrichment analysis. The GO terms that have gained, lost, or retained the same rank after n bootstrap runs are shown separately in the bottom plots. b) Top: Along the x-axis is the original proteomic enrichment analysis, and the y-axis corresponds to the bootstrapped proteomic enrichment analysis. The GO terms that have gained, lost, or retained the same rank after n bootstrap runs are shown separately in the bottom plots.

## Slide 42
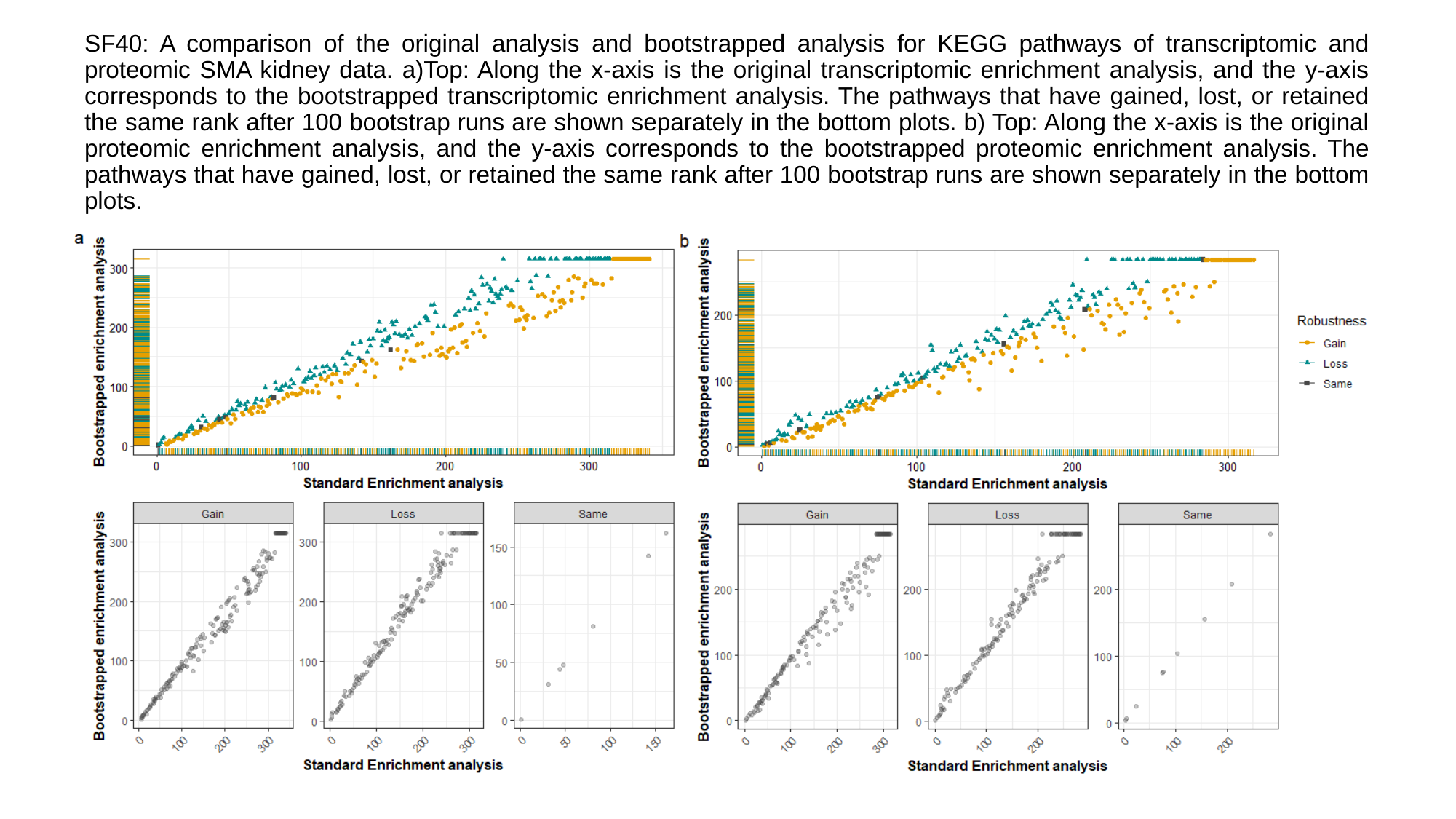

# SF40: A comparison of the original analysis and bootstrapped analysis for KEGG pathways of transcriptomic and proteomic SMA kidney data. a)Top: Along the x-axis is the original transcriptomic enrichment analysis, and the y-axis corresponds to the bootstrapped transcriptomic enrichment analysis. The pathways that have gained, lost, or retained the same rank after 100 bootstrap runs are shown separately in the bottom plots. b) Top: Along the x-axis is the original proteomic enrichment analysis, and the y-axis corresponds to the bootstrapped proteomic enrichment analysis. The pathways that have gained, lost, or retained the same rank after 100 bootstrap runs are shown separately in the bottom plots.

## Slide 43
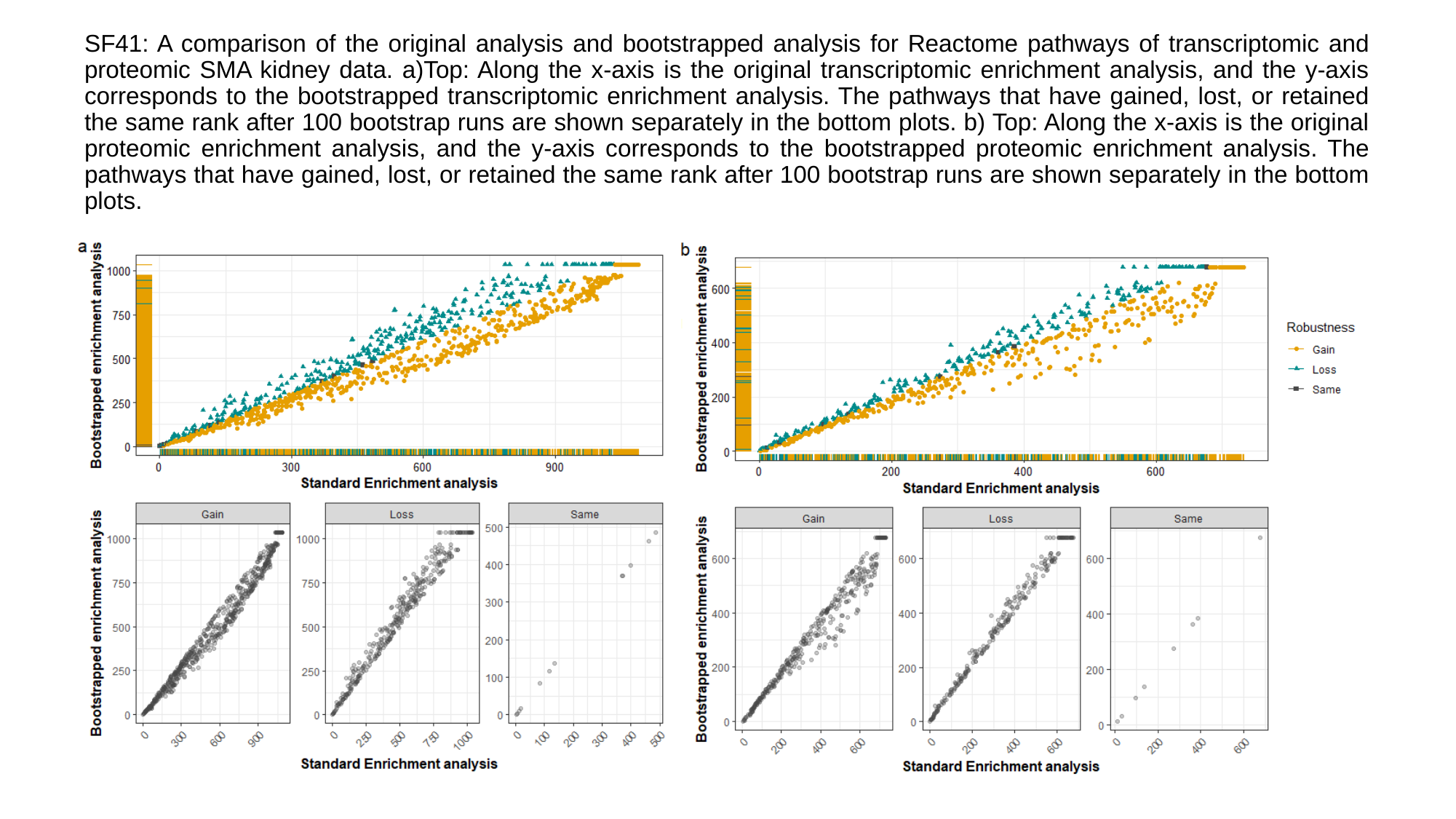

# SF41: A comparison of the original analysis and bootstrapped analysis for Reactome pathways of transcriptomic and proteomic SMA kidney data. a)Top: Along the x-axis is the original transcriptomic enrichment analysis, and the y-axis corresponds to the bootstrapped transcriptomic enrichment analysis. The pathways that have gained, lost, or retained the same rank after 100 bootstrap runs are shown separately in the bottom plots. b) Top: Along the x-axis is the original proteomic enrichment analysis, and the y-axis corresponds to the bootstrapped proteomic enrichment analysis. The pathways that have gained, lost, or retained the same rank after 100 bootstrap runs are shown separately in the bottom plots.

## Slide 44
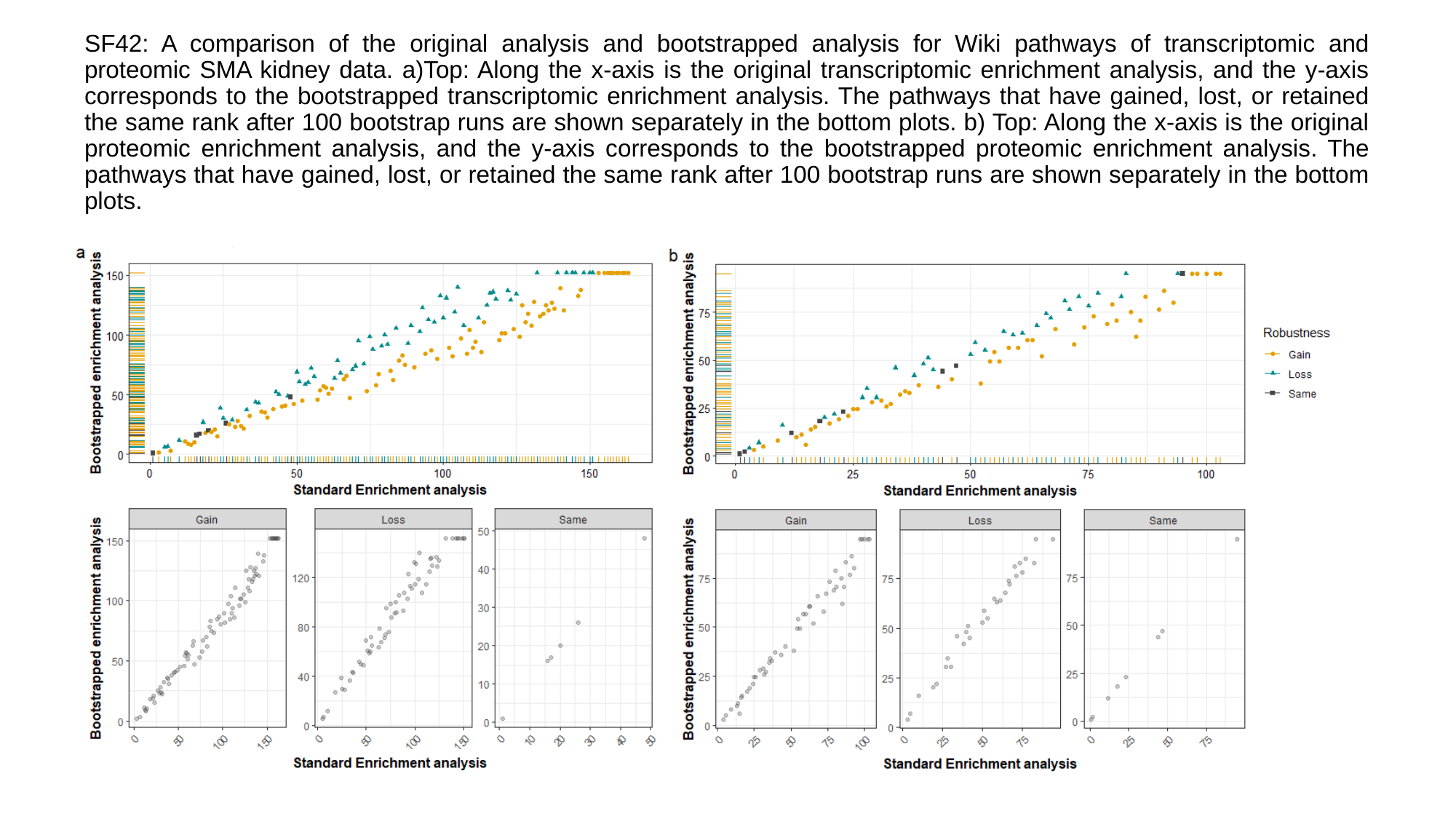

# SF42: A comparison of the original analysis and bootstrapped analysis for Wiki pathways of transcriptomic and proteomic SMA kidney data. a)Top: Along the x-axis is the original transcriptomic enrichment analysis, and the y-axis corresponds to the bootstrapped transcriptomic enrichment analysis. The pathways that have gained, lost, or retained the same rank after 100 bootstrap runs are shown separately in the bottom plots. b) Top: Along the x-axis is the original proteomic enrichment analysis, and the y-axis corresponds to the bootstrapped proteomic enrichment analysis. The pathways that have gained, lost, or retained the same rank after 100 bootstrap runs are shown separately in the bottom plots.

## Slide 45
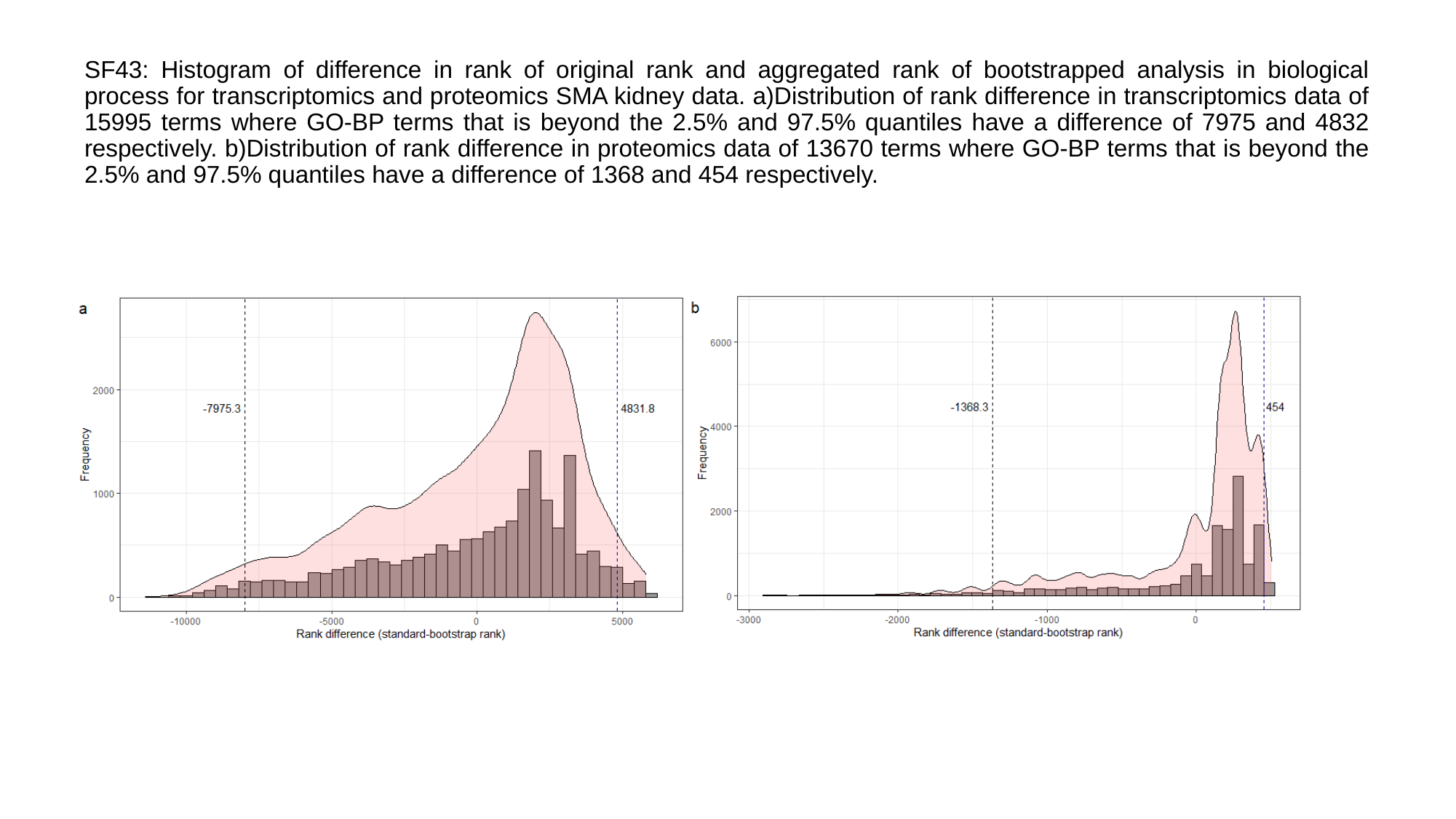

# SF43: Histogram of difference in rank of original rank and aggregated rank of bootstrapped analysis in biological process for transcriptomics and proteomics SMA kidney data. a)Distribution of rank difference in transcriptomics data of 15995 terms where GO-BP terms that is beyond the 2.5% and 97.5% quantiles have a difference of 7975 and 4832 respectively. b)Distribution of rank difference in proteomics data of 13670 terms where GO-BP terms that is beyond the 2.5% and 97.5% quantiles have a difference of 1368 and 454 respectively.

## Slide 46
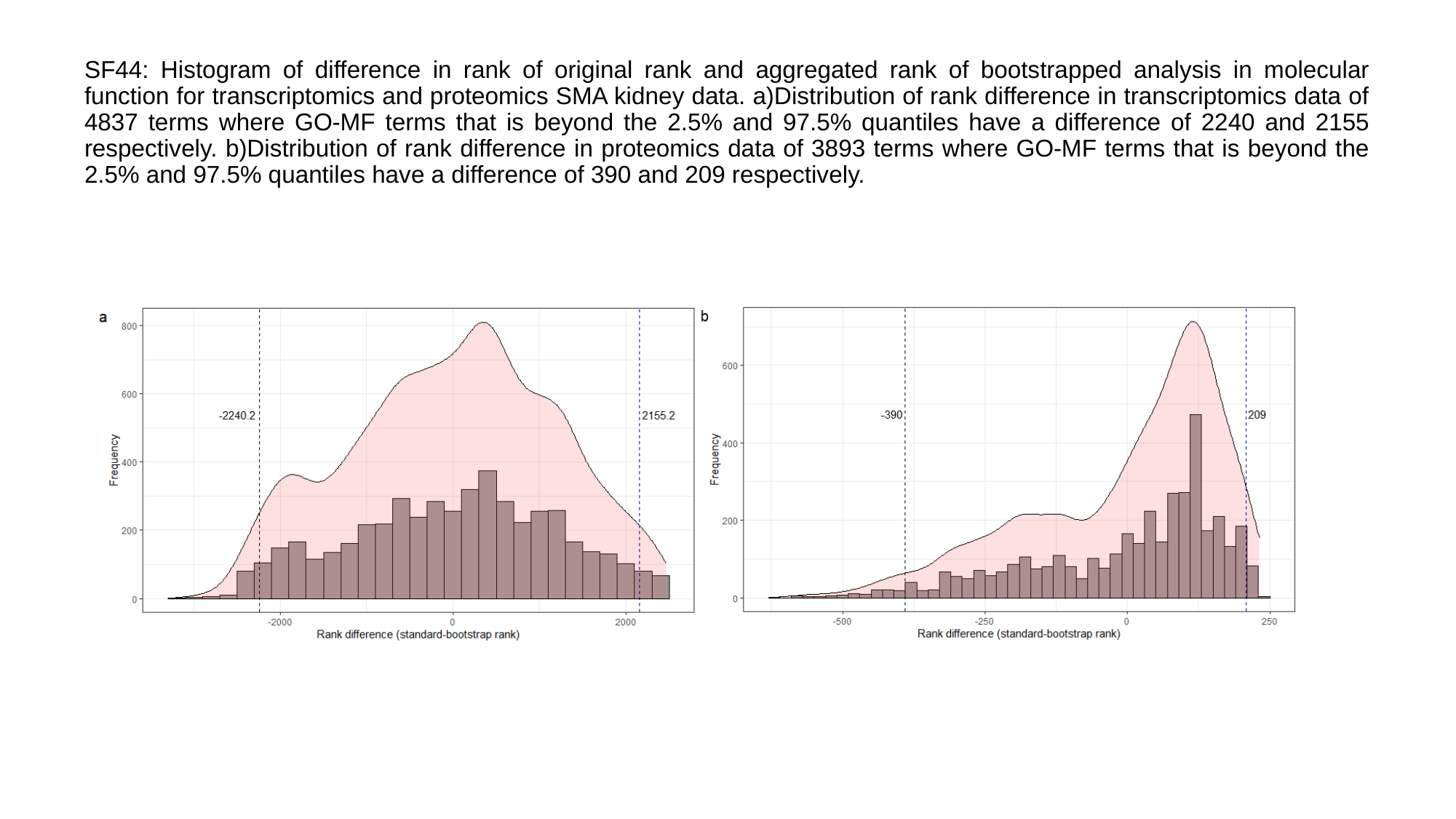

# SF44: Histogram of difference in rank of original rank and aggregated rank of bootstrapped analysis in molecular function for transcriptomics and proteomics SMA kidney data. a)Distribution of rank difference in transcriptomics data of 4837 terms where GO-MF terms that is beyond the 2.5% and 97.5% quantiles have a difference of 2240 and 2155 respectively. b)Distribution of rank difference in proteomics data of 3893 terms where GO-MF terms that is beyond the 2.5% and 97.5% quantiles have a difference of 390 and 209 respectively.

## Slide 47
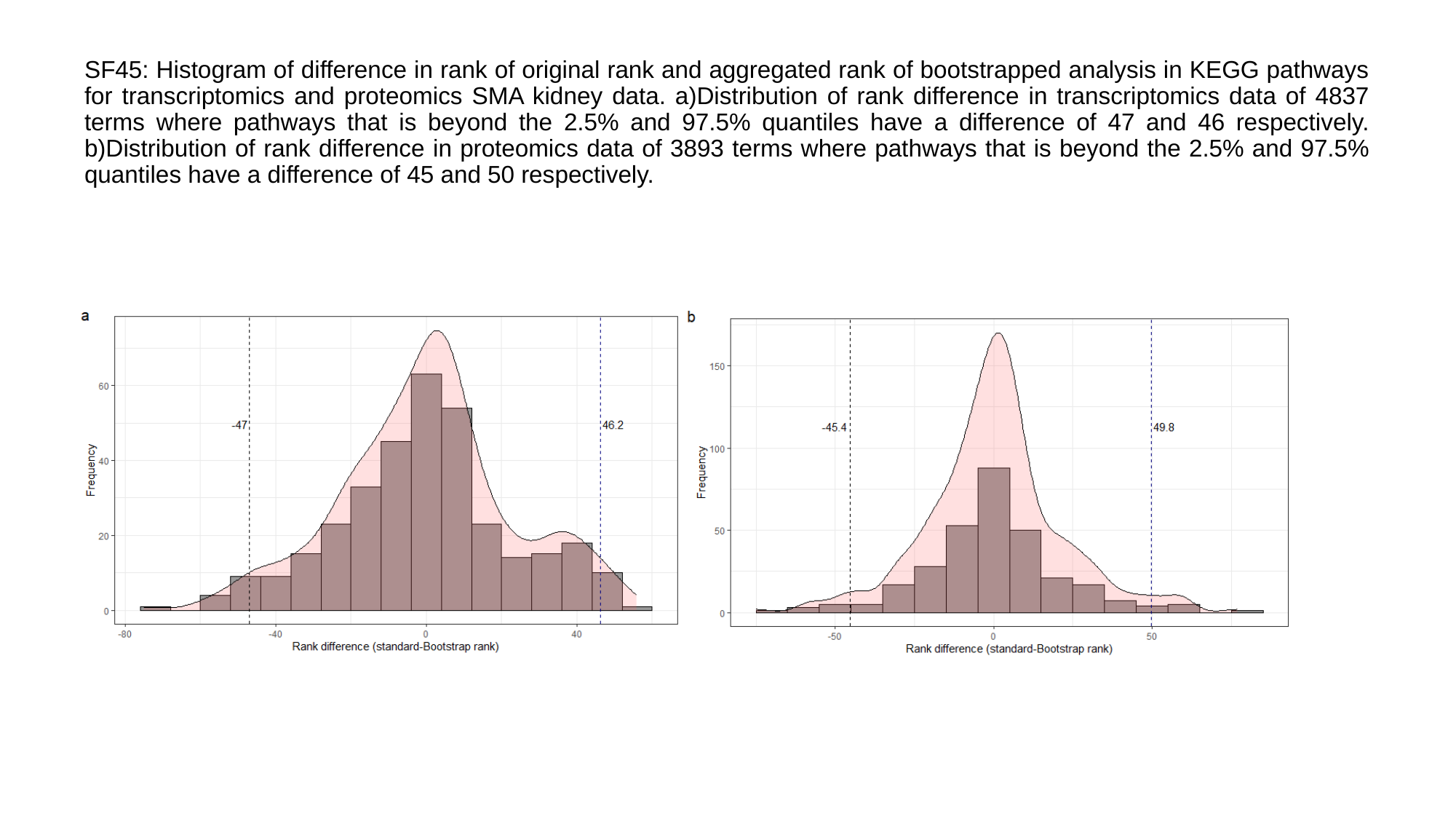

# SF45: Histogram of difference in rank of original rank and aggregated rank of bootstrapped analysis in KEGG pathways for transcriptomics and proteomics SMA kidney data. a)Distribution of rank difference in transcriptomics data of 4837 terms where pathways that is beyond the 2.5% and 97.5% quantiles have a difference of 47 and 46 respectively. b)Distribution of rank difference in proteomics data of 3893 terms where pathways that is beyond the 2.5% and 97.5% quantiles have a difference of 45 and 50 respectively.

## Slide 48
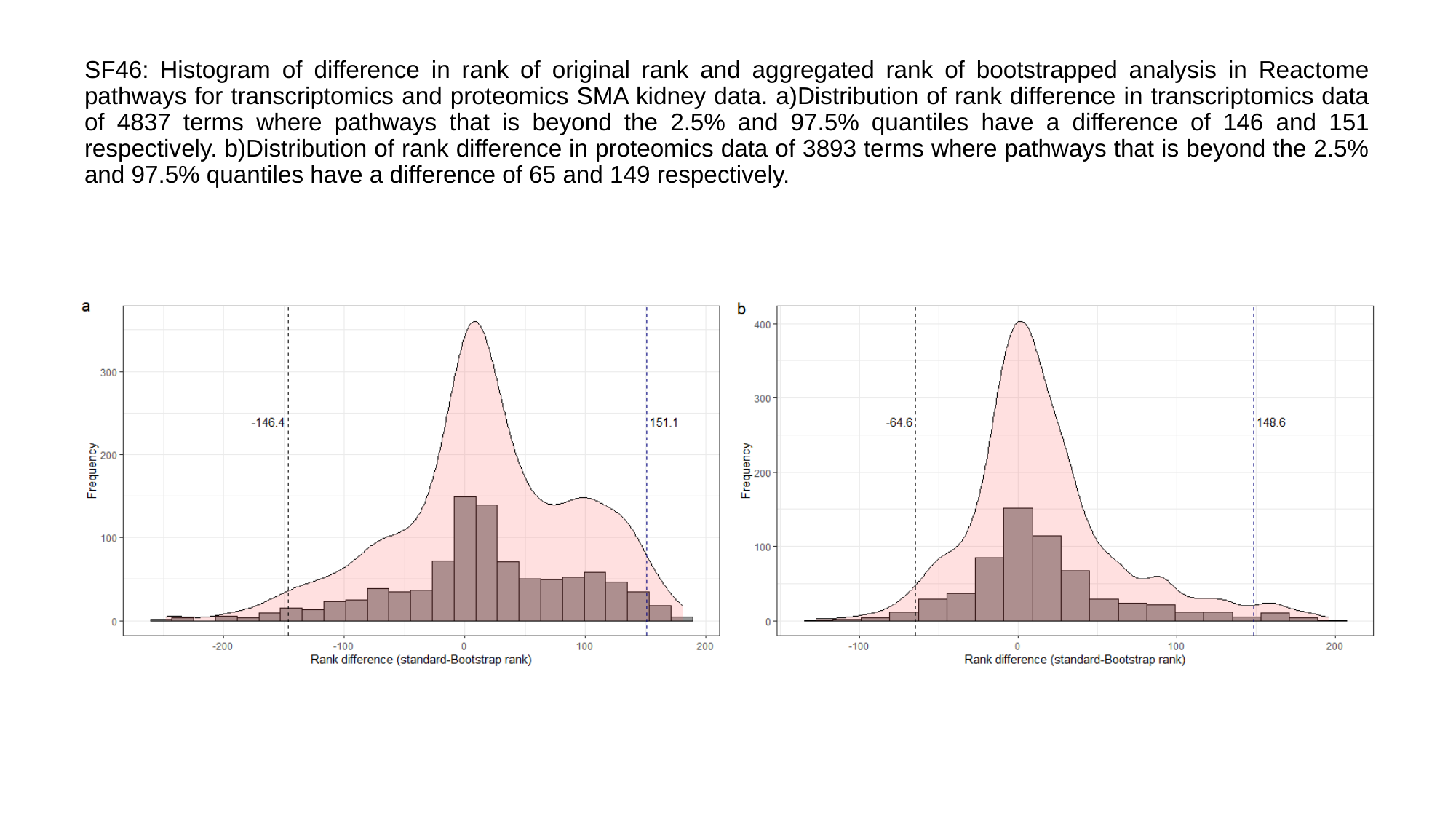

# SF46: Histogram of difference in rank of original rank and aggregated rank of bootstrapped analysis in Reactome pathways for transcriptomics and proteomics SMA kidney data. a)Distribution of rank difference in transcriptomics data of 4837 terms where pathways that is beyond the 2.5% and 97.5% quantiles have a difference of 146 and 151 respectively. b)Distribution of rank difference in proteomics data of 3893 terms where pathways that is beyond the 2.5% and 97.5% quantiles have a difference of 65 and 149 respectively.

## Slide 49
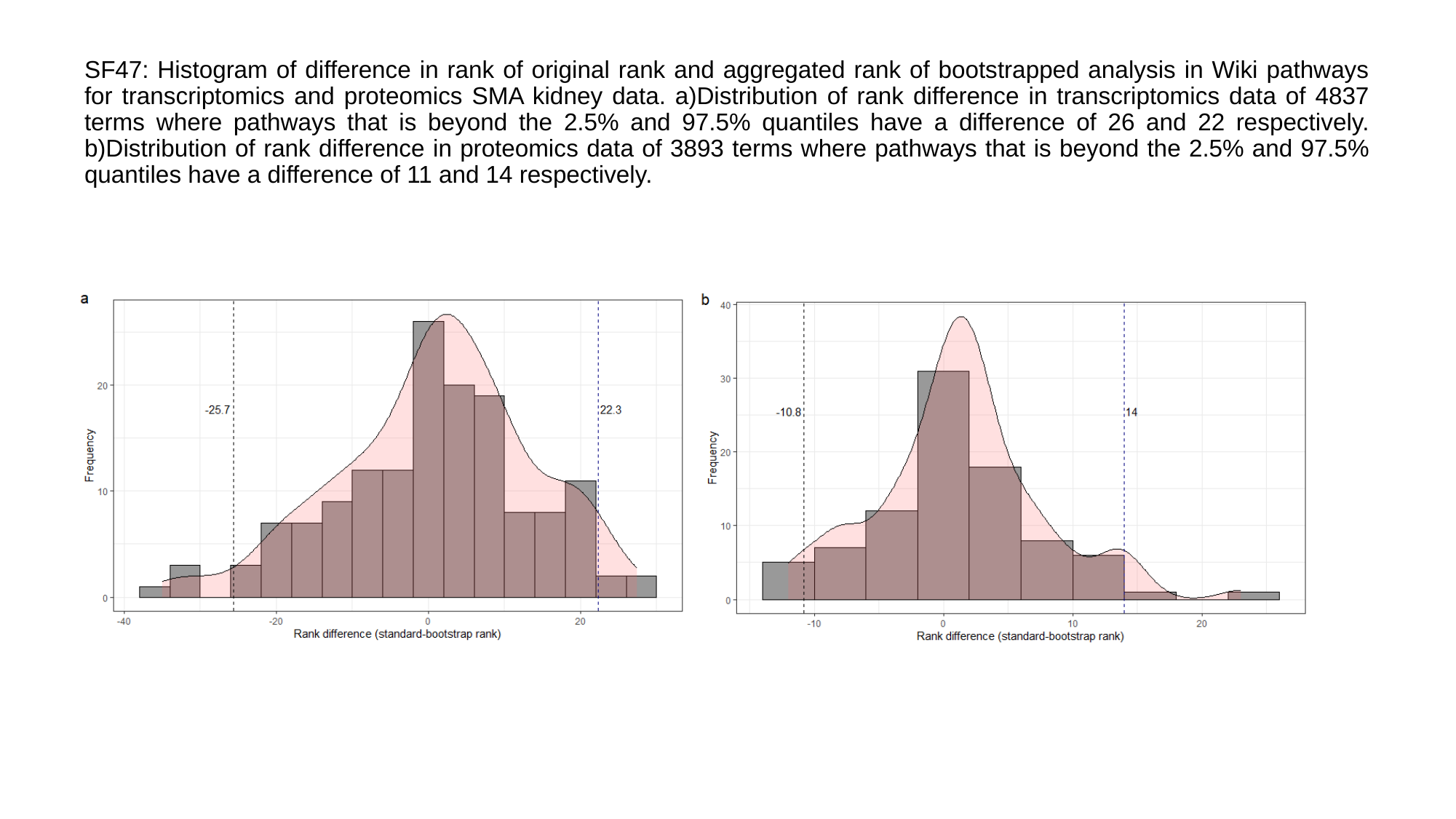

# SF47: Histogram of difference in rank of original rank and aggregated rank of bootstrapped analysis in Wiki pathways for transcriptomics and proteomics SMA kidney data. a)Distribution of rank difference in transcriptomics data of 4837 terms where pathways that is beyond the 2.5% and 97.5% quantiles have a difference of 26 and 22 respectively. b)Distribution of rank difference in proteomics data of 3893 terms where pathways that is beyond the 2.5% and 97.5% quantiles have a difference of 11 and 14 respectively.

## Slide 50
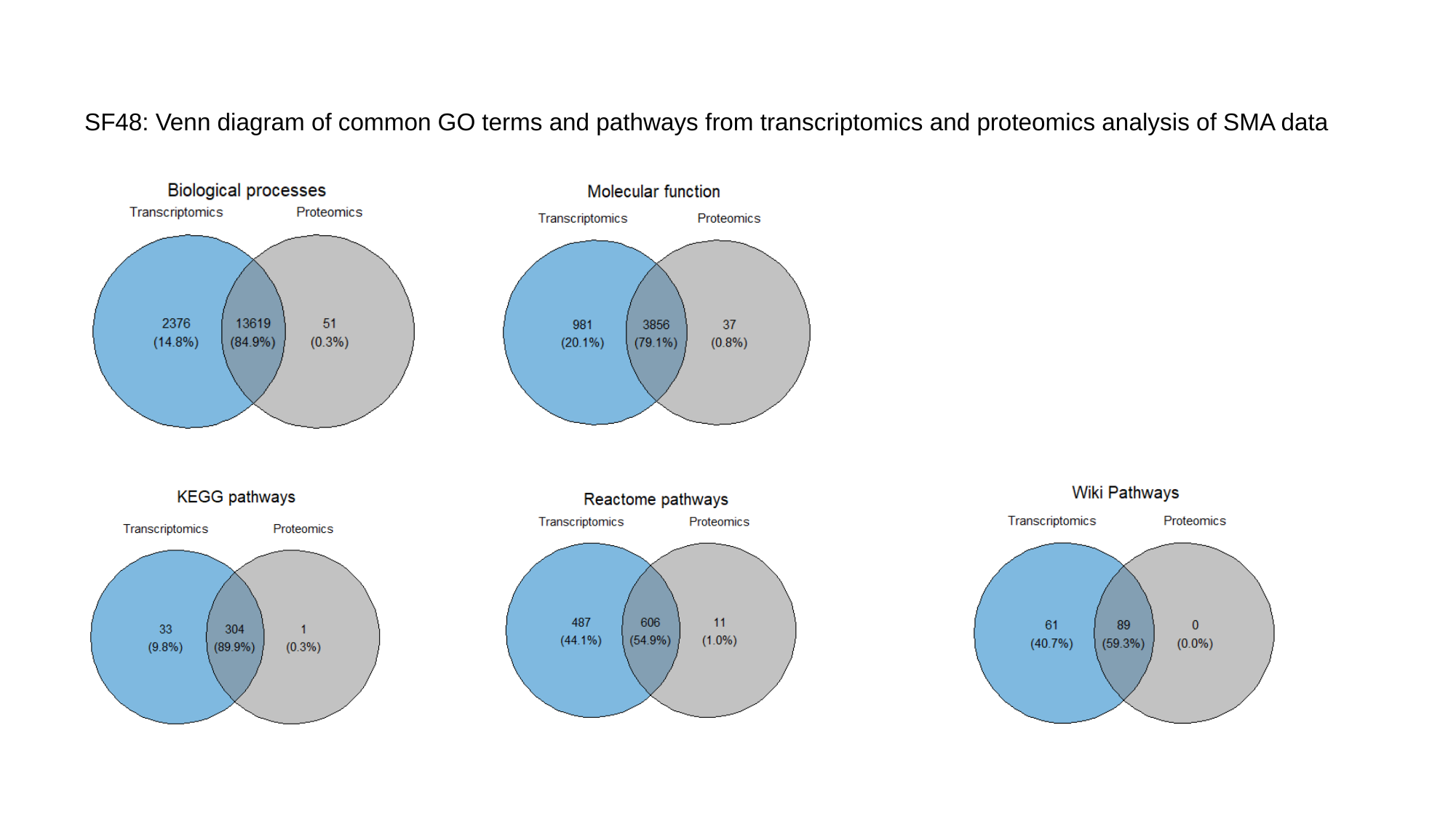

# SF48: Venn diagram of common GO terms and pathways from transcriptomics and proteomics analysis of SMA data

## Slide 51
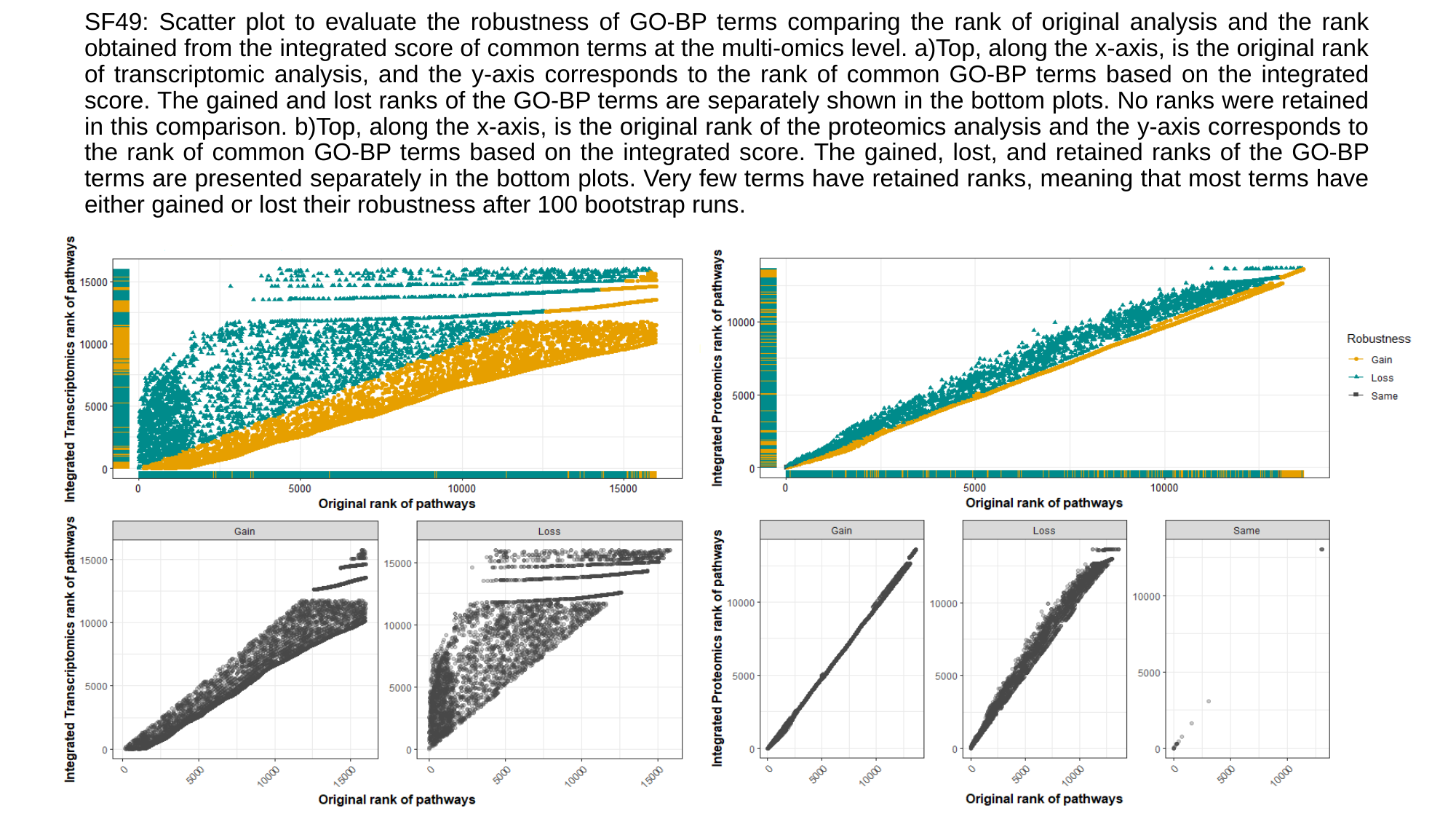

# SF49: Scatter plot to evaluate the robustness of GO-BP terms comparing the rank of original analysis and the rank obtained from the integrated score of common terms at the multi-omics level. a)Top, along the x-axis, is the original rank of transcriptomic analysis, and the y-axis corresponds to the rank of common GO-BP terms based on the integrated score. The gained and lost ranks of the GO-BP terms are separately shown in the bottom plots. No ranks were retained in this comparison. b)Top, along the x-axis, is the original rank of the proteomics analysis and the y-axis corresponds to the rank of common GO-BP terms based on the integrated score. The gained, lost, and retained ranks of the GO-BP terms are presented separately in the bottom plots. Very few terms have retained ranks, meaning that most terms have either gained or lost their robustness after 100 bootstrap runs.

## Slide 52
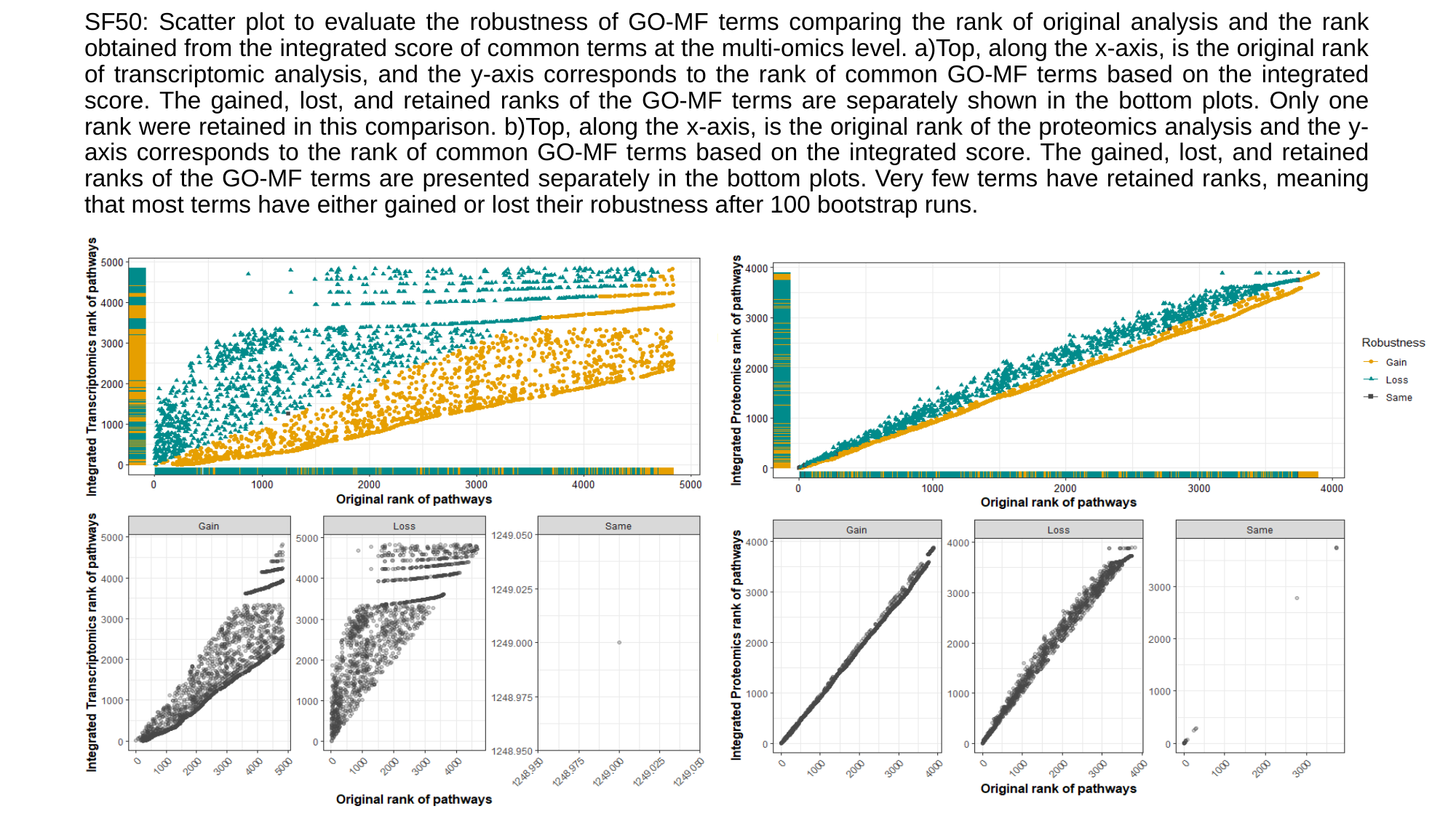

# SF50: Scatter plot to evaluate the robustness of GO-MF terms comparing the rank of original analysis and the rank obtained from the integrated score of common terms at the multi-omics level. a)Top, along the x-axis, is the original rank of transcriptomic analysis, and the y-axis corresponds to the rank of common GO-MF terms based on the integrated score. The gained, lost, and retained ranks of the GO-MF terms are separately shown in the bottom plots. Only one rank were retained in this comparison. b)Top, along the x-axis, is the original rank of the proteomics analysis and the y-axis corresponds to the rank of common GO-MF terms based on the integrated score. The gained, lost, and retained ranks of the GO-MF terms are presented separately in the bottom plots. Very few terms have retained ranks, meaning that most terms have either gained or lost their robustness after 100 bootstrap runs.

## Slide 53
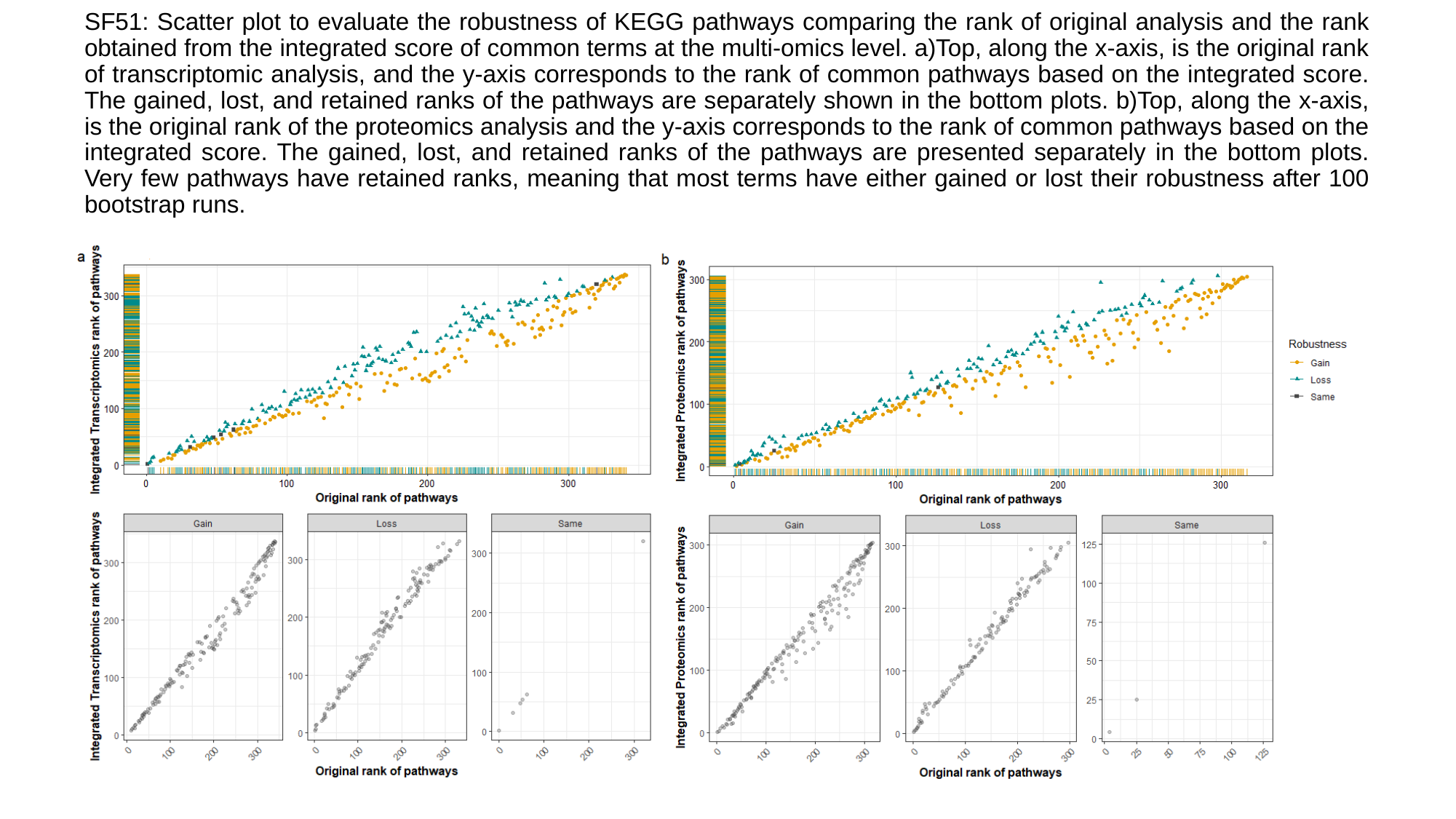

# SF51: Scatter plot to evaluate the robustness of KEGG pathways comparing the rank of original analysis and the rank obtained from the integrated score of common terms at the multi-omics level. a)Top, along the x-axis, is the original rank of transcriptomic analysis, and the y-axis corresponds to the rank of common pathways based on the integrated score. The gained, lost, and retained ranks of the pathways are separately shown in the bottom plots. b)Top, along the x-axis, is the original rank of the proteomics analysis and the y-axis corresponds to the rank of common pathways based on the integrated score. The gained, lost, and retained ranks of the pathways are presented separately in the bottom plots. Very few pathways have retained ranks, meaning that most terms have either gained or lost their robustness after 100 bootstrap runs.

## Slide 54
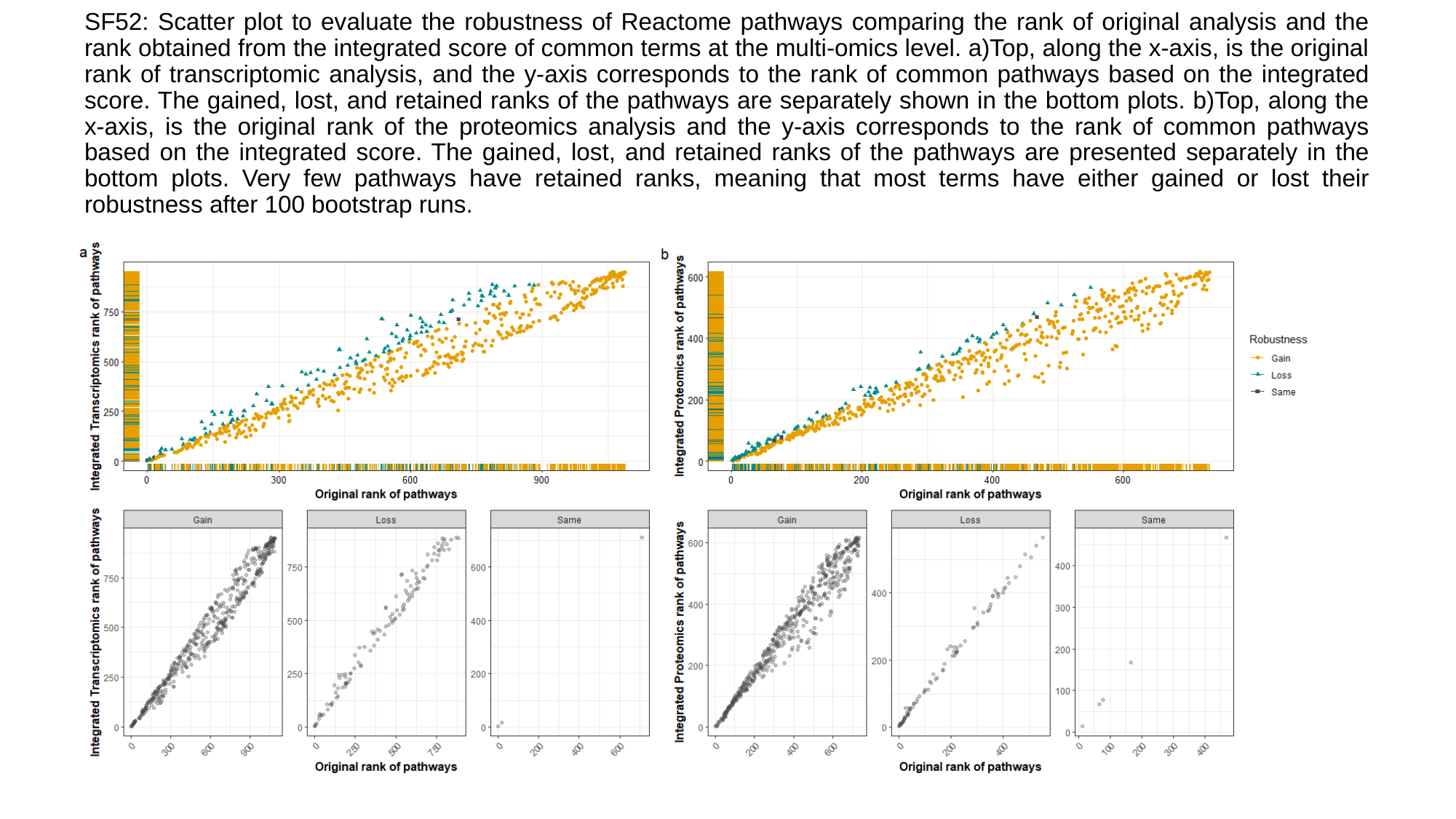

# SF52: Scatter plot to evaluate the robustness of Reactome pathways comparing the rank of original analysis and the rank obtained from the integrated score of common terms at the multi-omics level. a)Top, along the x-axis, is the original rank of transcriptomic analysis, and the y-axis corresponds to the rank of common pathways based on the integrated score. The gained, lost, and retained ranks of the pathways are separately shown in the bottom plots. b)Top, along the x-axis, is the original rank of the proteomics analysis and the y-axis corresponds to the rank of common pathways based on the integrated score. The gained, lost, and retained ranks of the pathways are presented separately in the bottom plots. Very few pathways have retained ranks, meaning that most terms have either gained or lost their robustness after 100 bootstrap runs.

## Slide 55
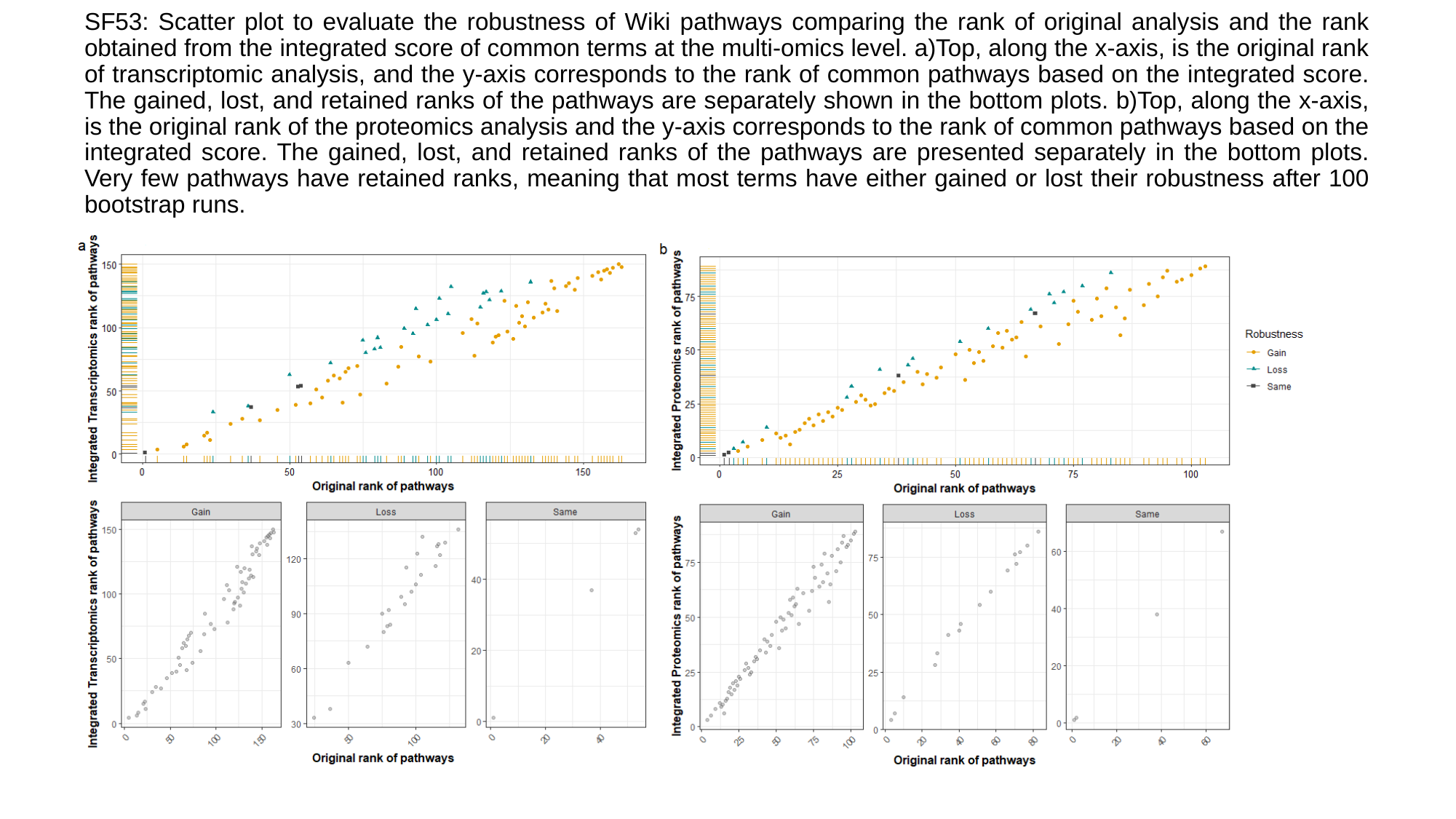

# SF53: Scatter plot to evaluate the robustness of Wiki pathways comparing the rank of original analysis and the rank obtained from the integrated score of common terms at the multi-omics level. a)Top, along the x-axis, is the original rank of transcriptomic analysis, and the y-axis corresponds to the rank of common pathways based on the integrated score. The gained, lost, and retained ranks of the pathways are separately shown in the bottom plots. b)Top, along the x-axis, is the original rank of the proteomics analysis and the y-axis corresponds to the rank of common pathways based on the integrated score. The gained, lost, and retained ranks of the pathways are presented separately in the bottom plots. Very few pathways have retained ranks, meaning that most terms have either gained or lost their robustness after 100 bootstrap runs.

## Slide 56
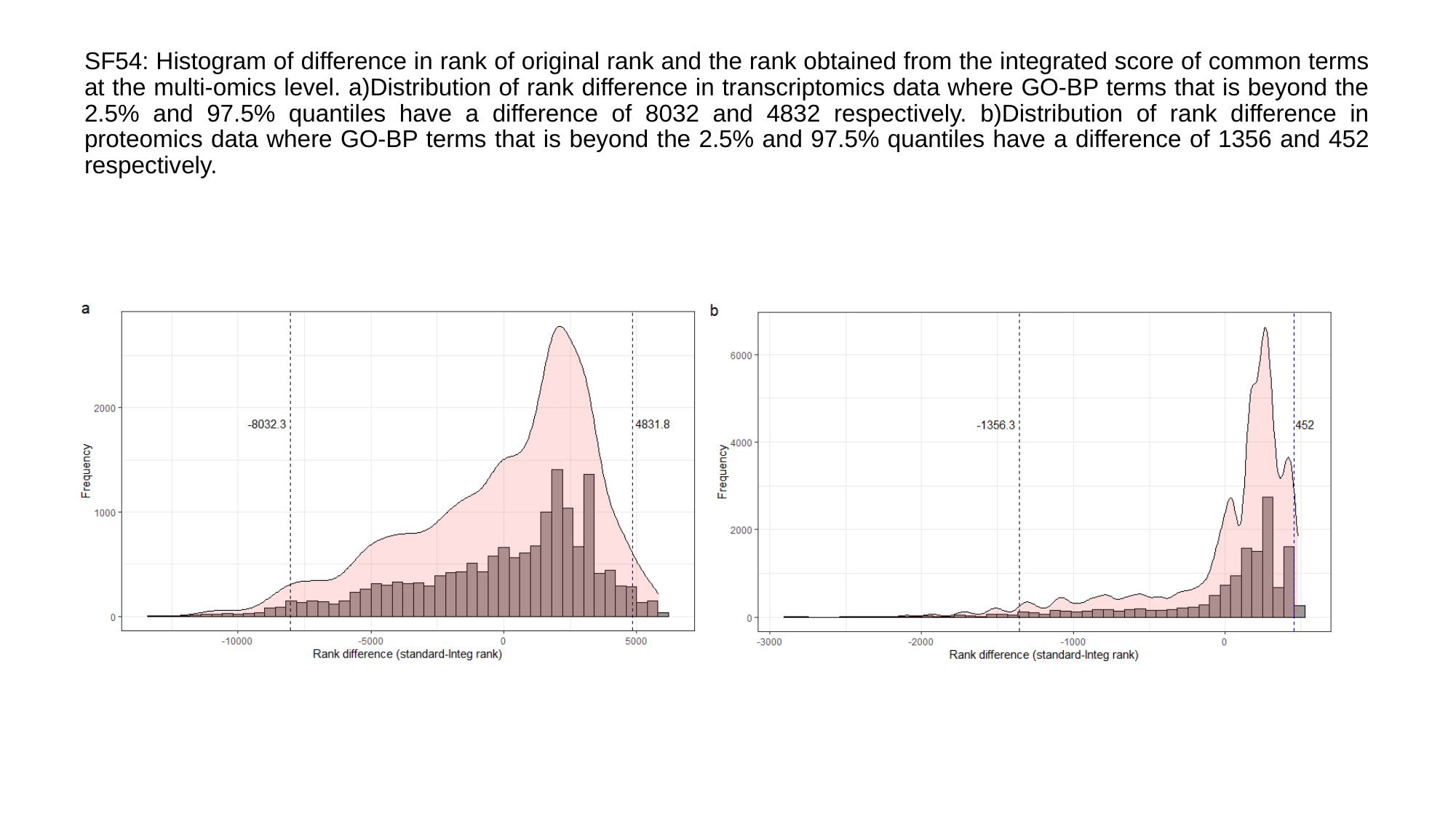

# SF54: Histogram of difference in rank of original rank and the rank obtained from the integrated score of common terms at the multi-omics level. a)Distribution of rank difference in transcriptomics data where GO-BP terms that is beyond the 2.5% and 97.5% quantiles have a difference of 8032 and 4832 respectively. b)Distribution of rank difference in proteomics data where GO-BP terms that is beyond the 2.5% and 97.5% quantiles have a difference of 1356 and 452 respectively.

## Slide 57
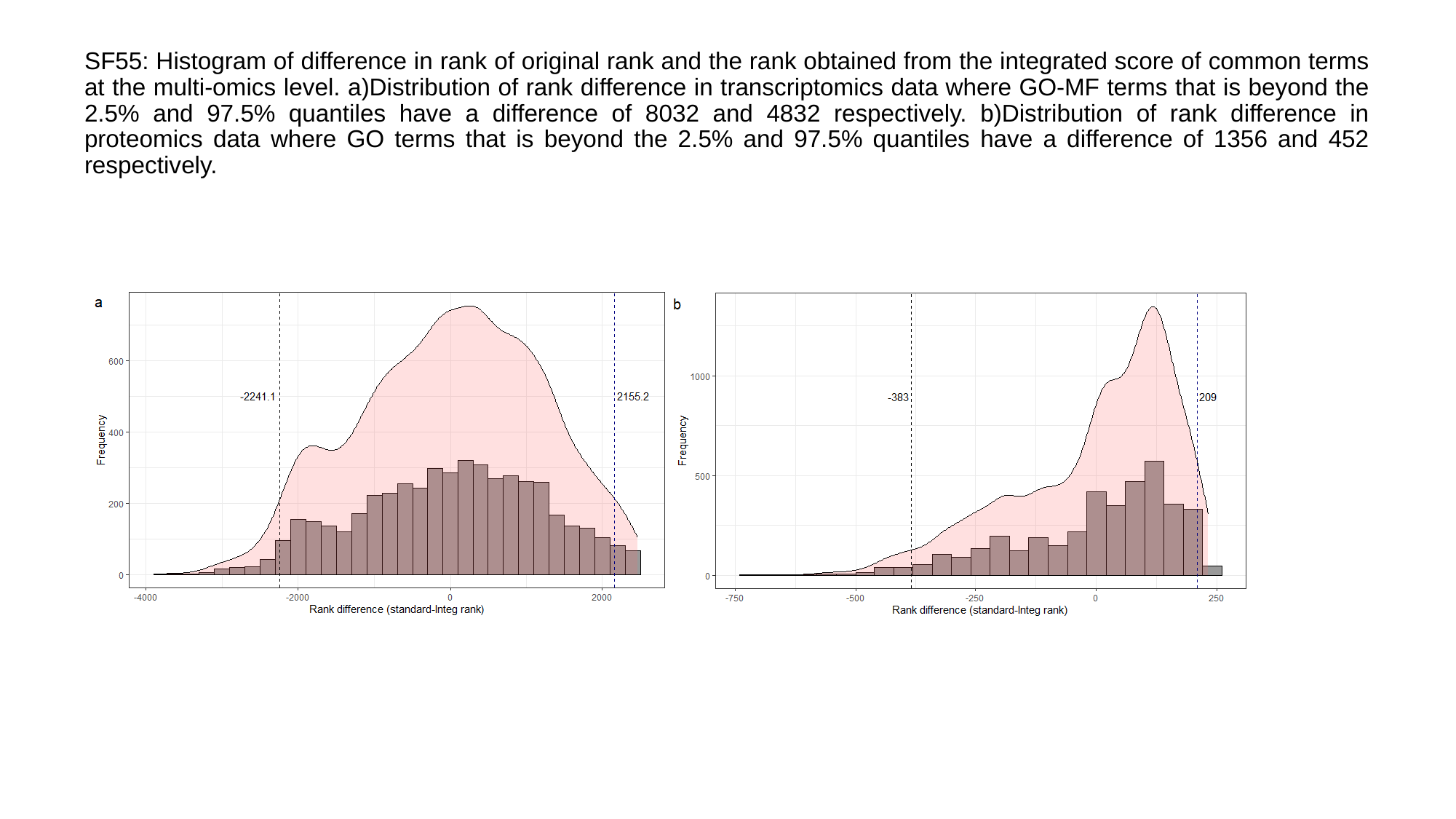

# SF55: Histogram of difference in rank of original rank and the rank obtained from the integrated score of common terms at the multi-omics level. a)Distribution of rank difference in transcriptomics data where GO-MF terms that is beyond the 2.5% and 97.5% quantiles have a difference of 8032 and 4832 respectively. b)Distribution of rank difference in proteomics data where GO terms that is beyond the 2.5% and 97.5% quantiles have a difference of 1356 and 452 respectively.

## Slide 58
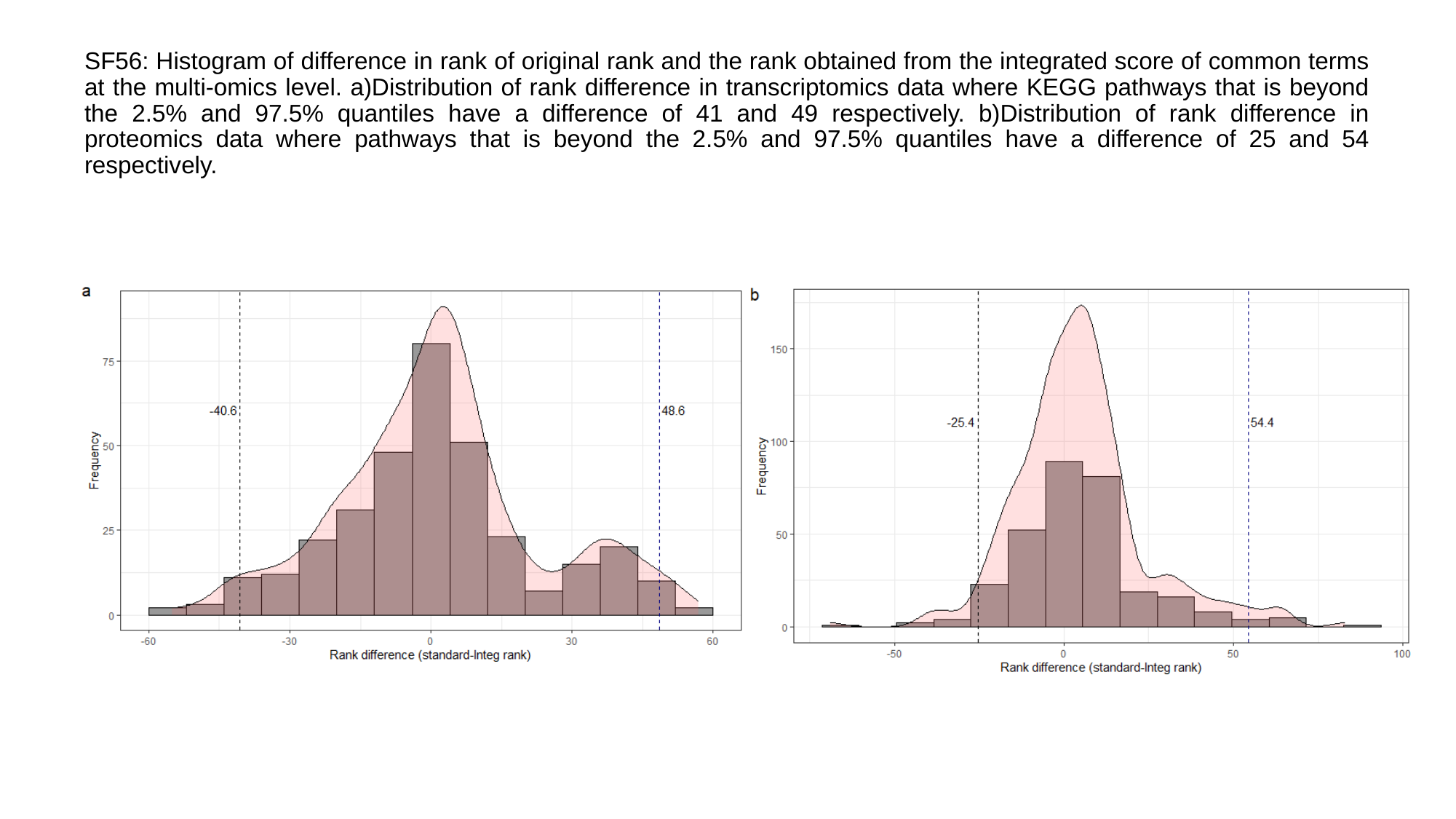

# SF56: Histogram of difference in rank of original rank and the rank obtained from the integrated score of common terms at the multi-omics level. a)Distribution of rank difference in transcriptomics data where KEGG pathways that is beyond the 2.5% and 97.5% quantiles have a difference of 41 and 49 respectively. b)Distribution of rank difference in proteomics data where pathways that is beyond the 2.5% and 97.5% quantiles have a difference of 25 and 54 respectively.

## Slide 59
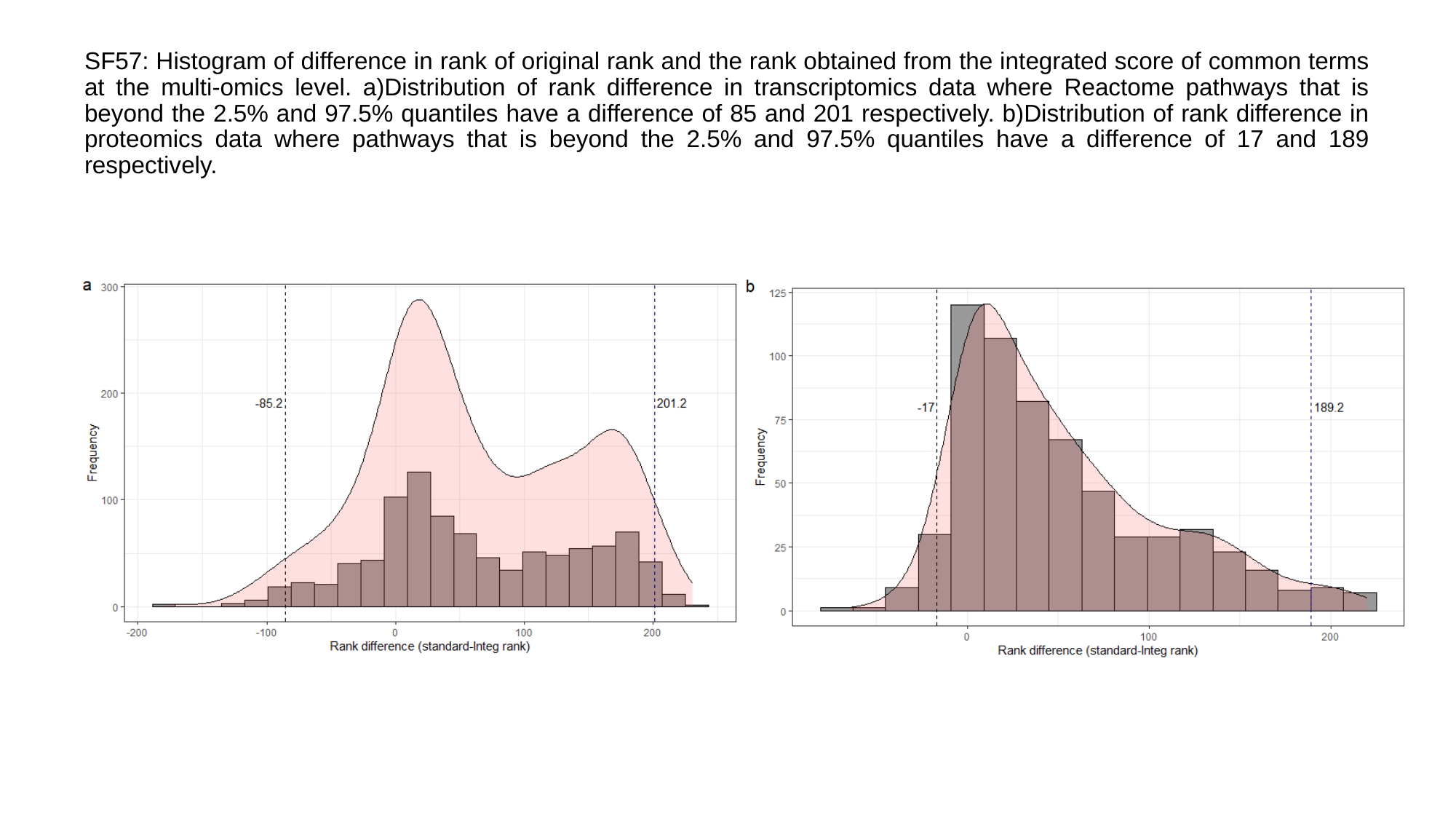

# SF57: Histogram of difference in rank of original rank and the rank obtained from the integrated score of common terms at the multi-omics level. a)Distribution of rank difference in transcriptomics data where Reactome pathways that is beyond the 2.5% and 97.5% quantiles have a difference of 85 and 201 respectively. b)Distribution of rank difference in proteomics data where pathways that is beyond the 2.5% and 97.5% quantiles have a difference of 17 and 189 respectively.

## Slide 60
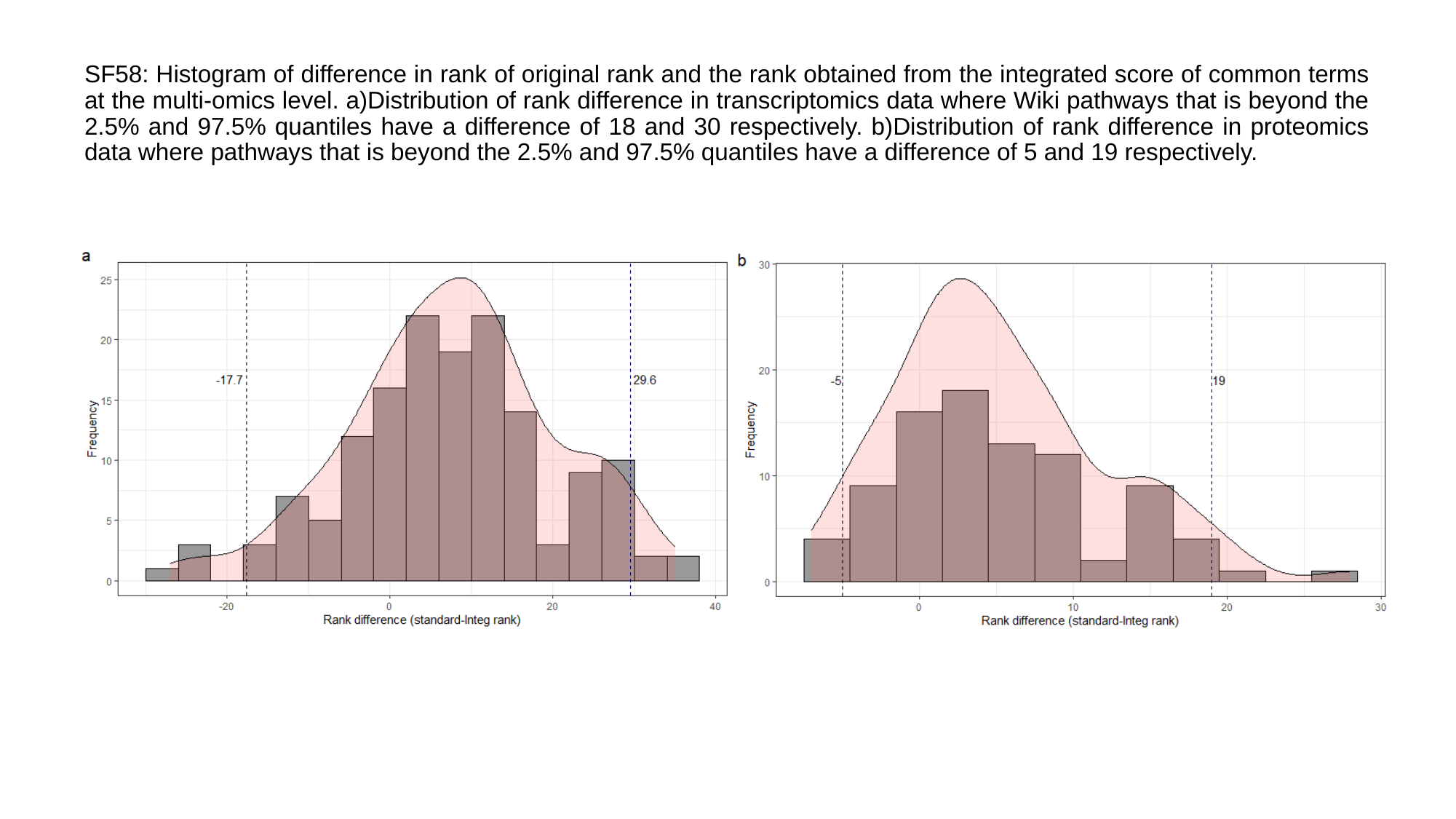

# SF58: Histogram of difference in rank of original rank and the rank obtained from the integrated score of common terms at the multi-omics level. a)Distribution of rank difference in transcriptomics data where Wiki pathways that is beyond the 2.5% and 97.5% quantiles have a difference of 18 and 30 respectively. b)Distribution of rank difference in proteomics data where pathways that is beyond the 2.5% and 97.5% quantiles have a difference of 5 and 19 respectively.
